# Determining the composition of gold nanoparticles: a compilation of shapes, sizes, and calculations using geometric considerations

**DOI:** 10.1007/s11051-016-3587-7

**Published:** 2016-10-03

**Authors:** Taizo Mori, Torsten Hegmann

**Affiliations:** 1 Chemical Physics Interdisciplinary Program, Liquid Crystal Institute, Kent State University, Kent, OH 44242-0001 USA; 2 World Premier International (WPI) Research Center for Materials Nanoarchitectonics (MANA), National Institute for Materials Science (NIMS), 1-1 Namiki, Tsukuba, 305-0044 Japan

**Keywords:** Gold nanoparticle, Nanocluster, Nanoparticle size, Nanoparticle shape, Nanoparticle composition, Modeling and simulation

## Abstract

**Abstract:**

Size, shape, overall composition, and surface functionality largely determine the properties and applications of metal nanoparticles. Aside from well-defined metal clusters, their composition is often estimated assuming a quasi-spherical shape of the nanoparticle core. With decreasing diameter of the assumed circumscribed sphere, particularly in the range of only a few nanometers, the estimated nanoparticle composition increasingly deviates from the real composition, leading to significant discrepancies between anticipated and experimentally observed composition, properties, and characteristics. We here assembled a compendium of tables, models, and equations for thiol-protected gold nanoparticles that will allow experimental scientists to more accurately estimate the composition of their gold nanoparticles using TEM image analysis data. The estimates obtained from following the routines described here will then serve as a guide for further analytical characterization of as-synthesized gold nanoparticles by other bulk (thermal, structural, chemical, and compositional) and surface characterization techniques. While the tables, models, and equations are dedicated to gold nanoparticles, the composition of other metal nanoparticle cores with face-centered cubic lattices can easily be estimated simply by substituting the value for the radius of the metal atom of interest.

**Graphical abstract:**

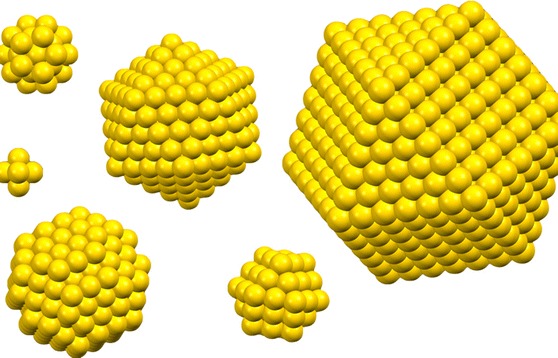

**Electronic supplementary material:**

The online version of this article (doi:10.1007/s11051-016-3587-7) contains supplementary material, which is available to authorized users.

## Introduction

Gold nanoparticles are everywhere! Aside from the curious and beautiful historic uses as colloidal additives to stain Roman glass in the fourth century and the discovery of the wondrous and different properties of colloidal gold by Michael Faraday in the mid nineteenth century (Tweney et al. 2004), gold nanoparticles and nanoclusters have penetrated almost every facet of science. Each year there are numerous educational and critical reviews on the use and study of gold nanoparticles for topics including in vitro diagnostics (Aillon et al. 2009; Almeida et al. 2011; Azzazy et al. 2006; Johnston et al. 2010; Khlebtsov and Dykman 2011; Mulder et al. 2009; Rosi and Mirkin 2005; Wolinsky and Grinstaff 2008), cancer diagnostics and therapy (Bhattacharyya et al. 2011; Chikkaveeraiah et al. 2012; Dreaden et al. 2011; Gindy and Prud’homme 2009; Jain et al. 2012; Kennedy et al. 2011; Lal et al. 2008; Perfezou et al. 2012; Wang and Thanou 2010; Yong et al. 2009; Zhang et al. 2013), biological and chemical sensors (Askim et al. 2013; Howes et al. 2014; Kim et al. 2012; Perfezou et al. 2012; Pingarron et al. 2008; Sepulveda et al. 2009; Stewart et al. 2008), catalysis (Crooks et al. 2001; Hou and Cronin 2013; Panigrahi et al. 2007; Sarina et al. 2013), gold *meta*-atoms for metamaterials (Ross et al. 2016), self-assembly (Bishop et al. 2009; Boeker et al. 2007; Grzelczak et al. 2010; Lin et al. 2006; Ofir et al. 2008), intrinsic chirality (Ben-Moshe et al. 2013; Gautier and Bürgi 2009; Guerrero-Martínez et al. 2011; Wang et al. 2013; Xia et al. 2011), and this list could go on. Mind you, 20 years after the beautiful and simple Brust-Schiffrin synthesis methods (Brust et al. 1994, 1995) were published we are finding it increasingly difficult to summarize the contents of review articles, not even individual papers, on gold nanoparticles. Our group, working on understanding interactions between functionalized gold nanoparticles and soft condensed matter, even added a few to this list of reviews, summarizing studies on these magnificent nanomaterials as versatile additives in liquid crystal phases (Hegmann et al. 2007; Qi and Hegmann 2008; Shivakumar et al. 2011; Stamatoiu et al. 2012). The search for “gold nanoparticle” in Thomson Reuters Web of Science shows an ever-increasing number of papers (several thousand), and just looking at the last couple of years, a Google search leads to a mind-boggling number of over 1.8 million hits. Numerous established chemical suppliers as well as smaller startup companies now sell gold nanoparticles, but the majority of laboratories it seems still enjoy synthesizing their own, partially perhaps for educational reasons, mainly most likely for their need of specific surface functionalization toward specific application- or research-driven size or shape requirements. We are not going to summarize these synthetic efforts here, largely because, as you can easily imagine, multiple review articles heretofore did exactly that already (Alexandridis 2011; Crooks et al. 2001; Ganguli et al. 2010; Gopidas et al. 2003; Grzelczak et al. 2008; Lohse and Murphy 2013; Lu et al. 2009; Mourdikoudis and Liz-Marzan 2013; Shan and Tenhu 2007; Walther and Mueller 2013; Zhao et al. 2013; Zhou et al. 2009).

The goal of this compendium of gold nanoparticle tables, which list and compare models to more precisely calculate size and composition, is to be there for the experimentalist once the synthesis is done, and when the characterization of the just prepared precious gold nanoparticles begins. Several groups have recently shown that precise nanoclusters (magic-numbered or not) can be made exclusively, or isolated from batches with initially wider size and shape distribution, with great reproducibility (vide infra). These synthesis pathways become more and more refined, as indicated by the increasing number of articles describing new clusters. Aided by high-resolution X-ray diffraction, mass spectrometry, single-particle (a combination of low dose and aberration-corrected) transmission electron microscopy (TEM) (Azubel et al. 2014), electrophoretic mobility calculations and electromigration (Pyell 2010), thermogravimetric analysis (TGA), elemental analysis, NMR, small-angle X-ray scattering, as well as an array of surface characterization techniques (Auger, AFM, XPS, etc.) (Baer et al. 2010), these gold nanoclusters can now be fully characterized and their composition unambiguously determined. However, most laboratories and research endeavors do not require the rigor and use of well-defined gold nanoclusters. In these cases, average size and well-defined surface chemistries are more critical as is the determination of the overall, yet average composition for a gold nanoparticle sample with a given size and likely shape distribution. The functions these nanoparticles need to perform, for example as plasmonic additives, in drug delivery, in biosensing, as surface-enhanced Raman probes, among many others, do nevertheless require a precise knowledge of the nanoparticle composition. Reproducibility is a great concern for biological and medical applications as well as various other uses in device technologies, affecting performance, reliability, and last but not least intellectual property (IP). To assist in this process and create a practical go-to guide to more precisely determine the core and, in part, ligand shell composition of synthesized nanoparticles, we collected and calculated compositions and best approximations and assembled these datasets based on the overall nanocluster shape. With more and more refined and higher-resolution transmission electron microscopy (TEM) instrumentation available on the market, experimentalists should be in a position to more accurately determine their nanoparticle core composition using the nanoparticle shape revealed by TEM and using the datasets and calculations collected in the tables to come.

The ligand shell is slightly more complicated. Thiolate-protected gold nanoparticles and nanoclusters dominate the literature by a large margin, and the presence of (RS–Au(I)–SR)^−^ and [RS(Au(I)–SR)_2_]^−^ “staple” and bridge motifs (Pensa et al. 2012) (better described as Au(0)-thiyl surface bonding (Reimers et al. 2016)) largely governed by the synthesis method as well as the size and shape of the particle or cluster complicates a precise prediction or calculation of the full composition of a given thiolate-capped gold nanoparticle sample. With analytical methods such as NMR (before and after I_2_ decomposition; i.e., oxidation of thiolates to disulfides), TGA, X-ray photoelectron spectroscopy (XPS) to the rescue, this hurdle can be overcome once the nanoparticle core composition is determined with some degree of precision.

First, however, we will provide an overview of the various polyhedral shapes relevant for gold nanoparticles. Most gold nanoparticles assumed to be quasi-spherical are in fact Platonic, Archimedean, or Catalan solids. Polyhedral gold nanoclusters are classified as icosahedra and face-centered cubic (fcc) polyhedra. The stable Ino’s and Marks’ decahedra are non-spherical shapes and are best described as ellipsoids. The icosahedral or Ino’s as well as Marks’ decahedral-based gold nanoclusters (with icosahedral structure considering the triangular faces and fcc structure when considering the rectangular faces) represent more molecular-like structures. The fcc-based gold nanoparticles have more bulk (plasmonic) structures.

**Fig. 1:**
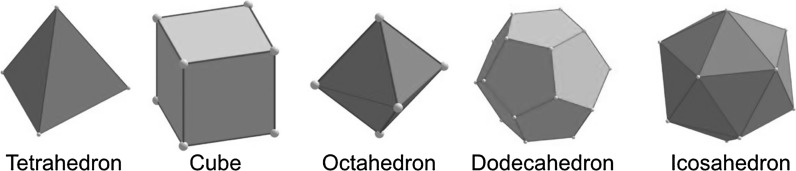
Five most relevant Platonic solids: Platonic solids are polyhedra whose faces are congruent regular polygons, where the same number of faces meets at every vertex

There are five platonic solids constructed by regular polygonal faces (Fig. 1), with tetrahedron, cube, and octahedron combined known as fcc unit cell substructures. Magic-numbered gold nanoclusters have regular icosahedral shapes.

**Fig. 2:**
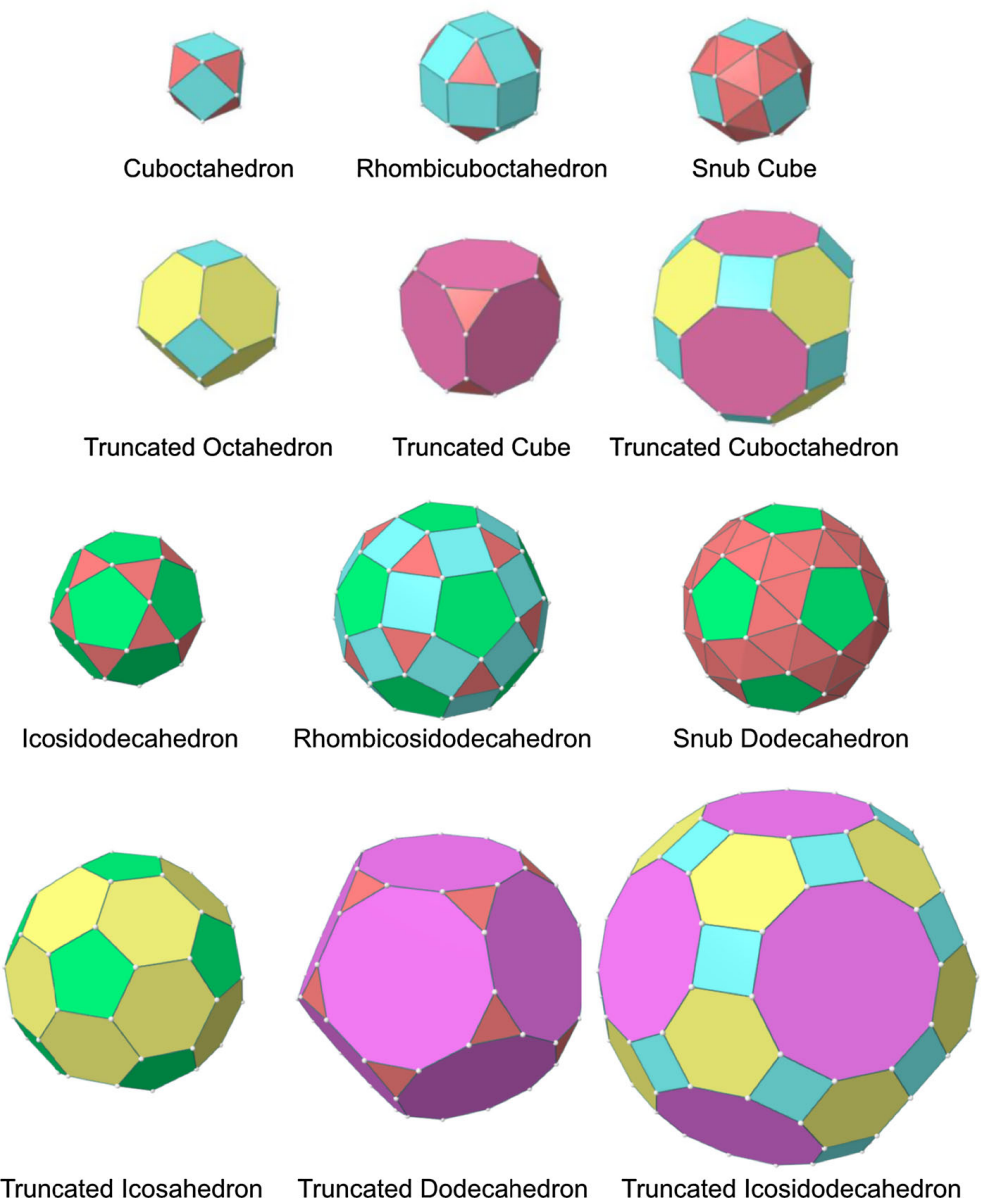
Depiction of several highly symmetric Archimedean solids with semi-regular color-coded regular polygonal faces

Two or three regular polygonal faces are needed to construct Archimedean solids, and truncating Platonic solids can compose them. As a result, Archimedean solids that are truncated from either tetrahedron, cube, or octahedron have fcc structures; other Archimedean solids have icosahedral structures as graphically shown in Fig. 2.

**Fig. 3:**
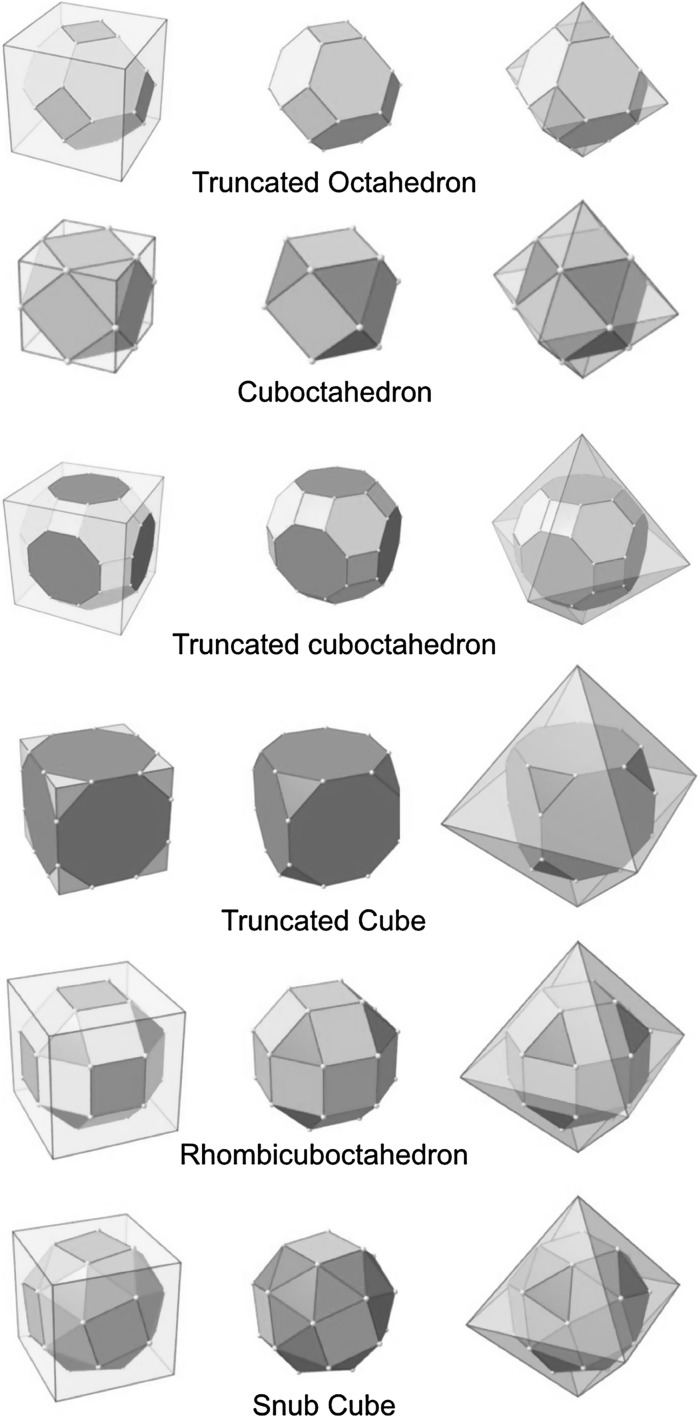

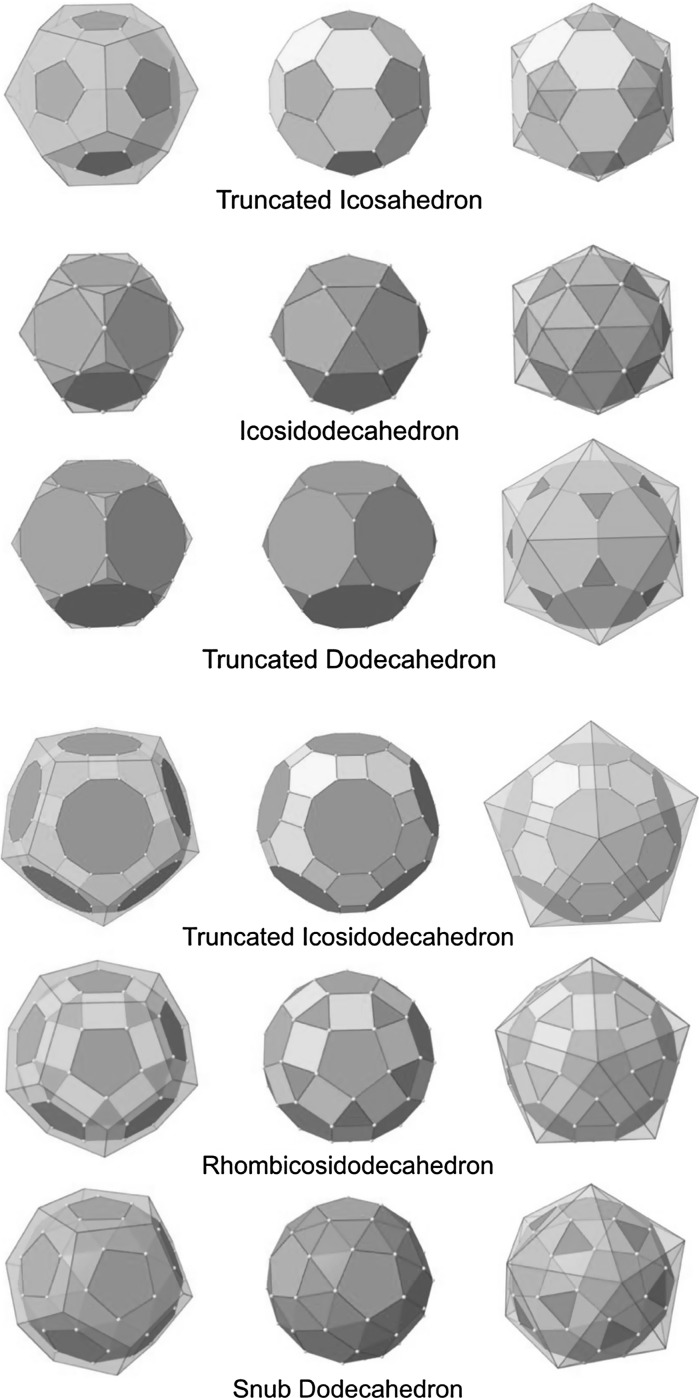
Truncation of Platonic solids with Platonic solids leads to Archimedean solids

**Fig. 4:**
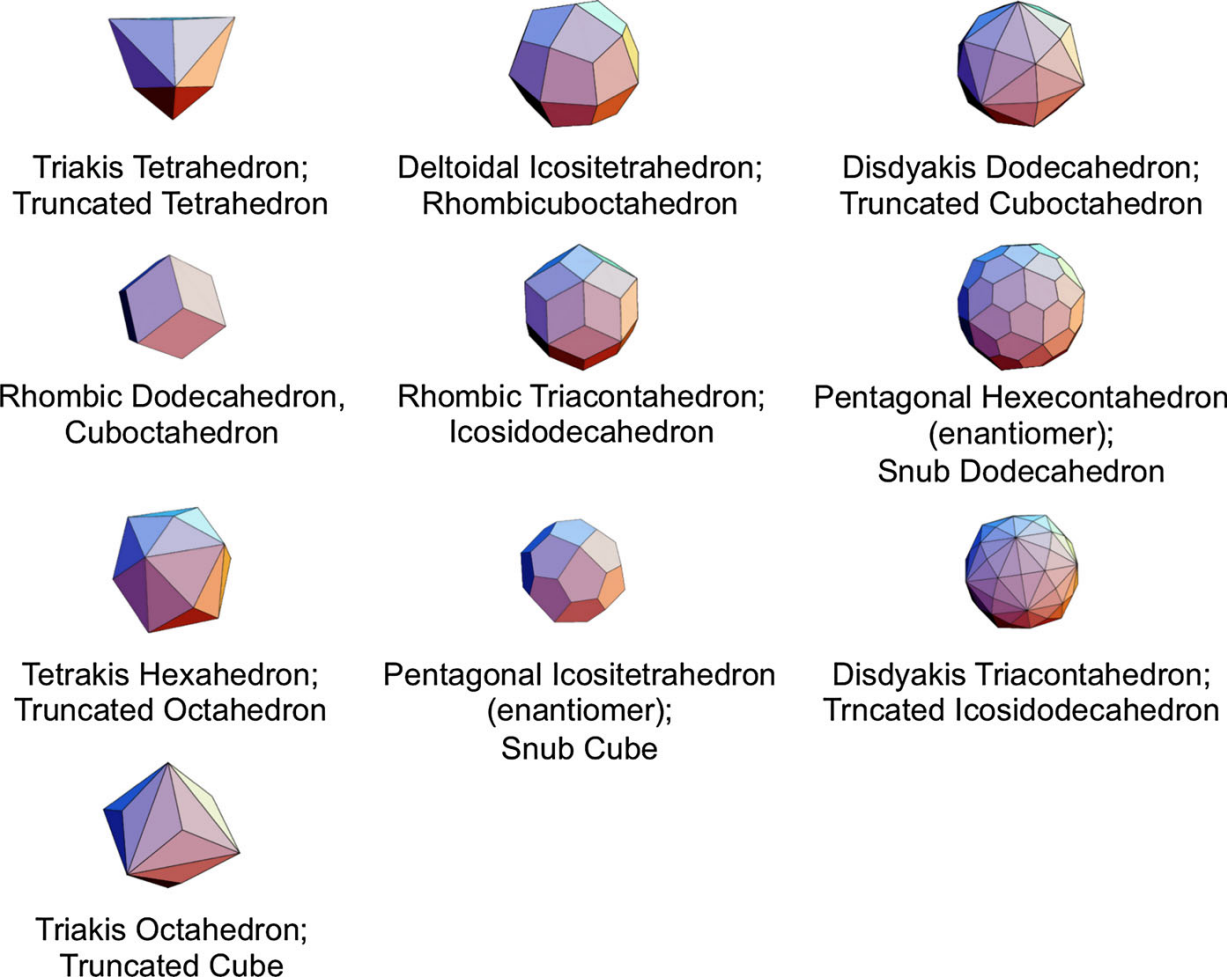
Depiction of Catalan solids defined as dual solids of Archimedean solids (the name of both Catalan solid and the dual solid is provided). Catalan solids are characterized by faces with non-equal edge lengths. Models taken from: Wolfram MathWorld; http://mathworld.wolfram.com

The process of obtaining these Archimedean solids by truncation is graphically shown in Fig. 3. The remaining Catalan solids are defined as dual solids of Archimedean solids. As their faces are not regular polygonal, it is expected that the cores of metal clusters could not be Catalan solid structures; however, ligand shells of Archimedean metal clusters could have Catalan solid structures (Fig. 4).

## Tables, models, and calculations

### Spherical versus icosahedral model

**Fig. 5:**
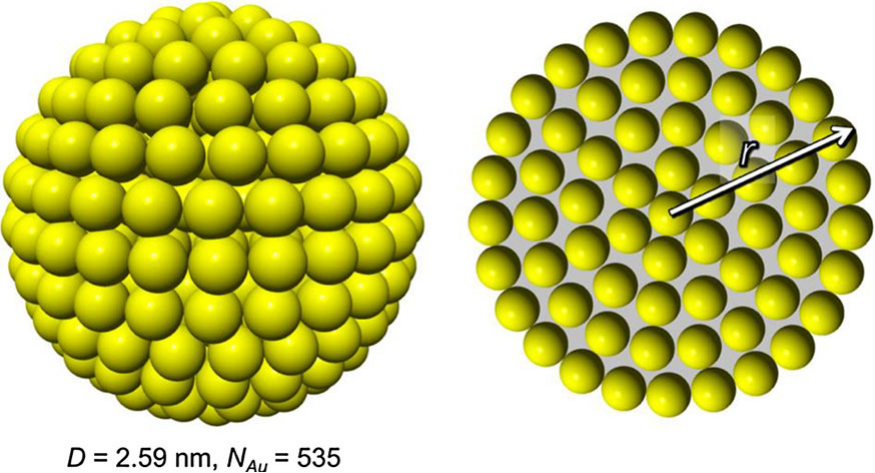
A quasi-spherical representation of a Au_535_ cluster used to demonstrate the estimation of the gold nanocluster size and composition via the frequently used quasi-spherical model

The number of gold atoms *N*_Au_ is commonly calculated assuming a quasi-spherical gold nanoparticle shape using Eq. 1 (Leff et al. 1995):

1 $$ N_{\text{Au}} = \frac{{4\pi r^{3} }}{{3V_{\text{Au}} }} = \frac{{\pi D^{3} }}{102} $$

where *V*_Au_ is the volume of the Au atom (*V*_Au_ = 17 Å^3^), *r* and *D* are the radius and the diameter of the gold nanoparticle, respectively, and *r*_Au_ is the radius of the gold atom (*r*_*Au*_ = 1.44 Å) with *r* = (2*n* + 1)*r*_Au_, where *n* is the number of full gold atoms along the radius of the nanoparticle as shown in Fig. 5.

**Fig. 6:**
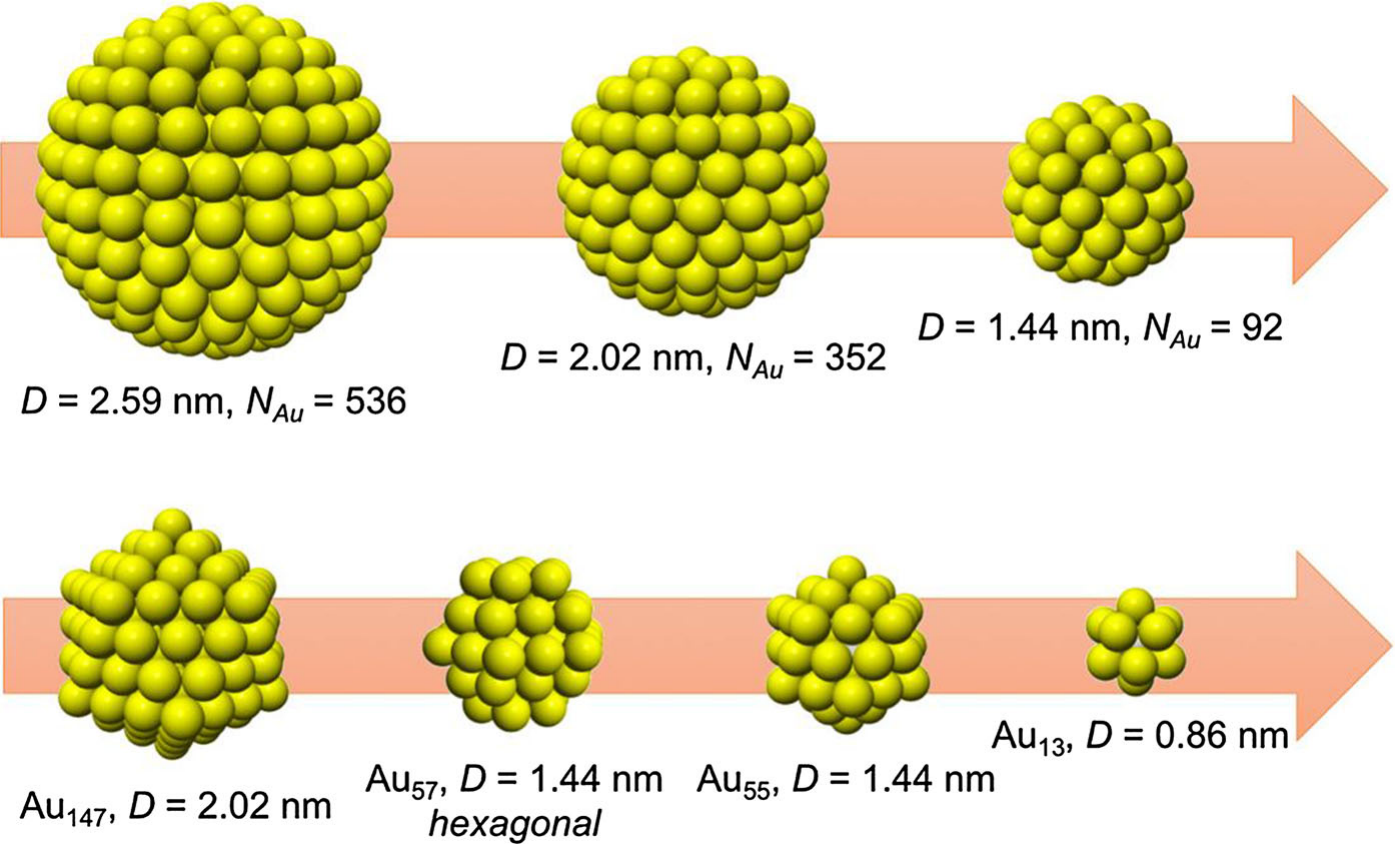
Shape determines composition: The* graphic* shows how as the size of the gold nanoparticle decreases and polyhedral shapes of well-defined clusters dominate, the spherical model to calculate the gold nanoparticle composition is less and less accurate

As the size of the gold nanoparticles change (decrease or increase), and polyhedral shapes of specific clusters are now increasingly synthetically accessible, the use of this simple model becomes, as we will see, more and more problematic. Figure 6 shows that with the progression from a larger to a smaller nanoparticle (or cluster) the assumption of a quasi-spherical nanoparticle leads to a larger and larger discrepancy in composition.

**Fig. 7:**
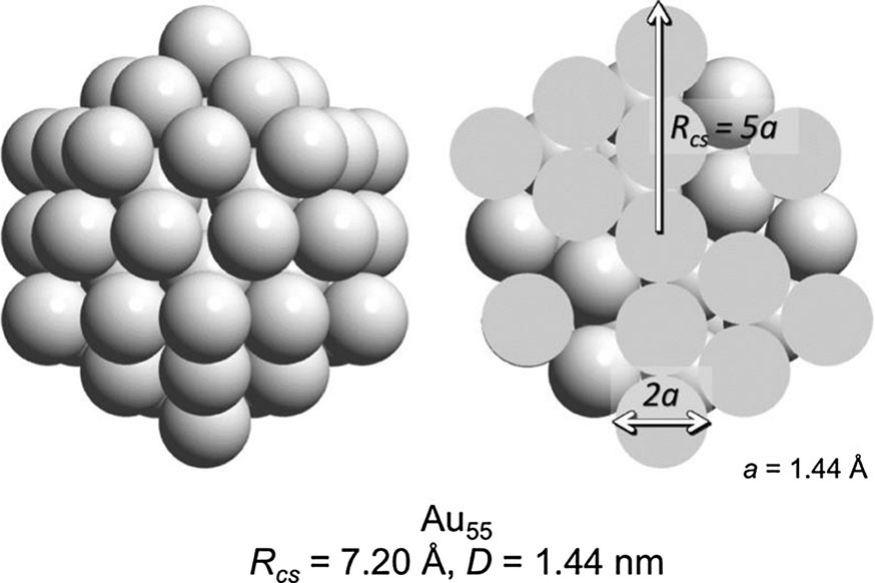
Full and cross-sectional view of a Au_55_ cluster highlighting the radius of the circumscribed sphere in the quasi-spherical model

**Fig. 8:**
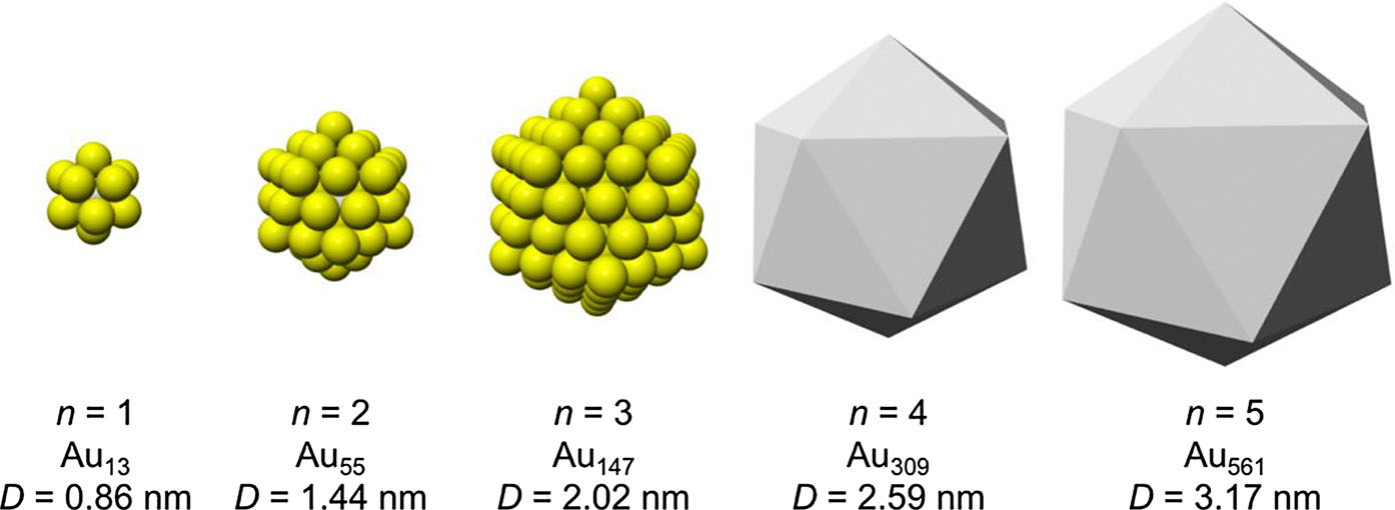
Magic-numbered gold clusters and their composition and diameter calculated assuming a regular icosahedral shape

For the calculation we first introduce the radius of the circumscribed sphere for an icosahedron *R*_cs_. For a Au_55_ cluster, this leads to *R*_cs_ = 7.2 Å and a diameter of *D* = 1.44 nm as shown in Fig. 7. For magic-numbered gold clusters, whose overall shape is best described as regular icosahedral, the following equations give the number of gold atoms in a regular icosahedron *N*_ico_ (Eq. 2), the radius of the circumscribed sphere for an icosahedron *R*_cs_ (Eq. 3), and the magic number *M*_*N*_ (Eq. 4):

2 $$ N_{\text{ico}} = \frac{{V_{\text{ico}} }}{{V_{\text{Au}} }} $$

3 $$ R_{\text{cs}} = \frac{{\sqrt {10 + 2\sqrt 5 } }}{4}L_{\text{ico}} $$

4 $$ M_{N} = \frac{1}{3}\left( {2n + 1} \right)\left( {5n^{2} + 5n + 3} \right) $$

where *V*_ico_ is the volume of the icosahedron, *V*_Au_ the volume of the gold atom, *L*_ico_ the edge length of the icosahedron, and where *R*_cs_ = (2*n* + 1)*r*_Au_ as mentioned earlier. For the magic-sized clusters with *n* = 1–5, this results in a composition of these clusters as shown in Fig. 8.

**Fig. 9:**
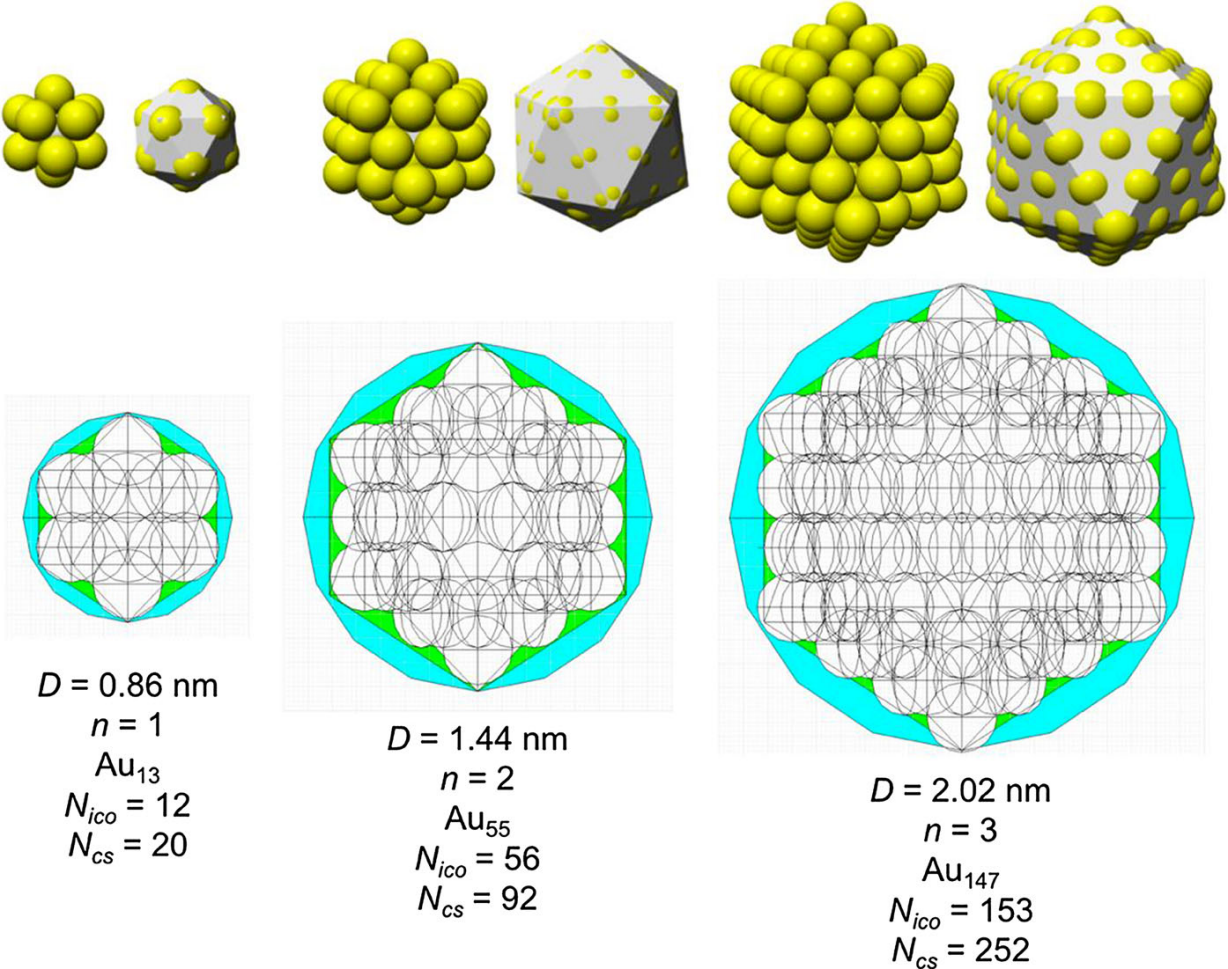
Magic-numbered gold clusters with *n* = 1–3 (Au_13_, Au_55_, and Au_147_) above and discrepancy in the number of gold atoms between quasi-sphere and regular icosahedron

Table 1 lists the values and Fig. 9 graphically shows the obvious discrepancies between a quasi-spherical model and the regular icosahedral shape and the comparison of the number of gold atoms obtained for both shapes, where the values of *N*_cs_ (the number of gold atoms obtained from the radius of the circumscribed sphere) and *N*_ico_ (the number of gold atoms in the regular icosahedron) divided by the magic number *M*_*N*_ should be close to 1 for a match between experiment and calculation. As one can see, this number quickly and rather drastically deviates when a quasi-spherical model is used. For example, for nanoparticles in the size range of 2–3 nm, which are frequently described in the literature, the quasi-spherical model overestimates the number of gold atoms by a factor of over 1.7.

**Table 1:** Comparison of the number of gold atoms using a quasi-spherical and a more precise icosahedral model

*n*	*M*_*N*_	*R*_cs_/Å	*D*/nm	*L*_ico_/Å	*V*_ico_ */*Å^3^	*N*_cs_	*N*_ico_	*N*_cs_/*M*_*N*_	*N*_ico_/*M*_*N*_
1	13	4.32	0.86	4.54	204.34	19.9	12.0	1.528	0.925
2	55	7.2	1.44	7.57	946.04	92.0	55.6	1.672	1.012
3	147	10.08	2.02	10.60	2595.95	252.4	152.7	1.717	1.039
4	309	12.96	2.59	13.63	5517.34	536.4	324.5	1.736	1.050
5	561	15.84	3.17	16.66	10,073.50	979.3	592.6	1.746	1.056

*V*_ico_ is the volume of the icosahedron, and *N*_cs_ and *N*_ico_ are the number of gold atoms in the circumscribed sphere and the regular icosahedron, respectively

The magnitude of deviation between the number of gold atoms in a nanoparticle or nanocluster varying with the use of either the quasi-spherical or more accurate polyhedral model largely depends on the shape of individual particles. High-resolution transmission electron microscopy (HR-TEM) is nowadays more than capable of revealing precise nanoparticle shapes and sizes, particularly when coupled with TEM tomography. Exact calculation of the nanoparticle composition should therefore be rather straightforward using the equations for the various polyhedral shapes provided in Section S1 of the Electronic Supplementary Material (ESM). Table S2A expands on the comparison between the quasi-spherical model and models of many other polyhedral shapes that are, or could be, formed by gold (or other coinage metal) nanoparticles or clusters for a specific radius of the circumscribed sphere of *R*_c_ = 10.08 Å (related to a Au_147_ cluster with regular icosahedral shape). We again provide a measure of the goodness of fit between the quasi-spherical and the given polyhedral model by the ratio between the number of gold atoms obtained from each model *N*_c_/*N*_*v*_, where *N*_c_ is again the number of gold atoms contained within the circumscribed sphere and *N*_*v*_ is the number of gold atoms calculated from the volume of the polyhedron. For Catalan solids *N*_ve_ is the number of gold atoms calculated from the volume of the sphere with vertex radius (*N*_ve_ = *V*_*Rv*_/*V*_Au_) calculated from the volume of the sphere with vertex radius in Catalan solids (*V*_*Rv*_). A related table showing the discrepancies between the quasi-spherical model and specific polyhedral shapes assuming a radius of the circumscribed sphere of *R*_c_ = 7.2 Å (related to a Au_55_ cluster with regular icosahedral shape) is given in the ESM (Section S2, Table S2B).

### Decahedral model

Jiang et al. (2003) Now that we have general sense of the influence of the nanoparticle or nanocluster shape, we will look at specific and commonly found polyhedral nanocluster shapes and calculate the composition of the clusters depending on the specific sub-type and size. Specifically, we will look at decahedra, Archimedean cubes, and Archimedean icosahedra. Table 2 provides a complete list of pentagonal decahedra, Ino’s decahedra, and Marks’ decahedra by generation (layers of gold atoms around the center atoms) giving the number of gold atoms at the surface, the total number of gold atoms of the cluster, the parent cluster that is covered with another layer of gold atoms as well as their calculated heights and widths. Pentagonal decahedra are composed of ten faces of icosahedra. Ino’s and Marks’ decahedra are created by truncating pentagonal decahedra. Thus, these decahedra have icosahedral structures on triangular faces and fcc structures on rectangular faces.

**Table 2:**
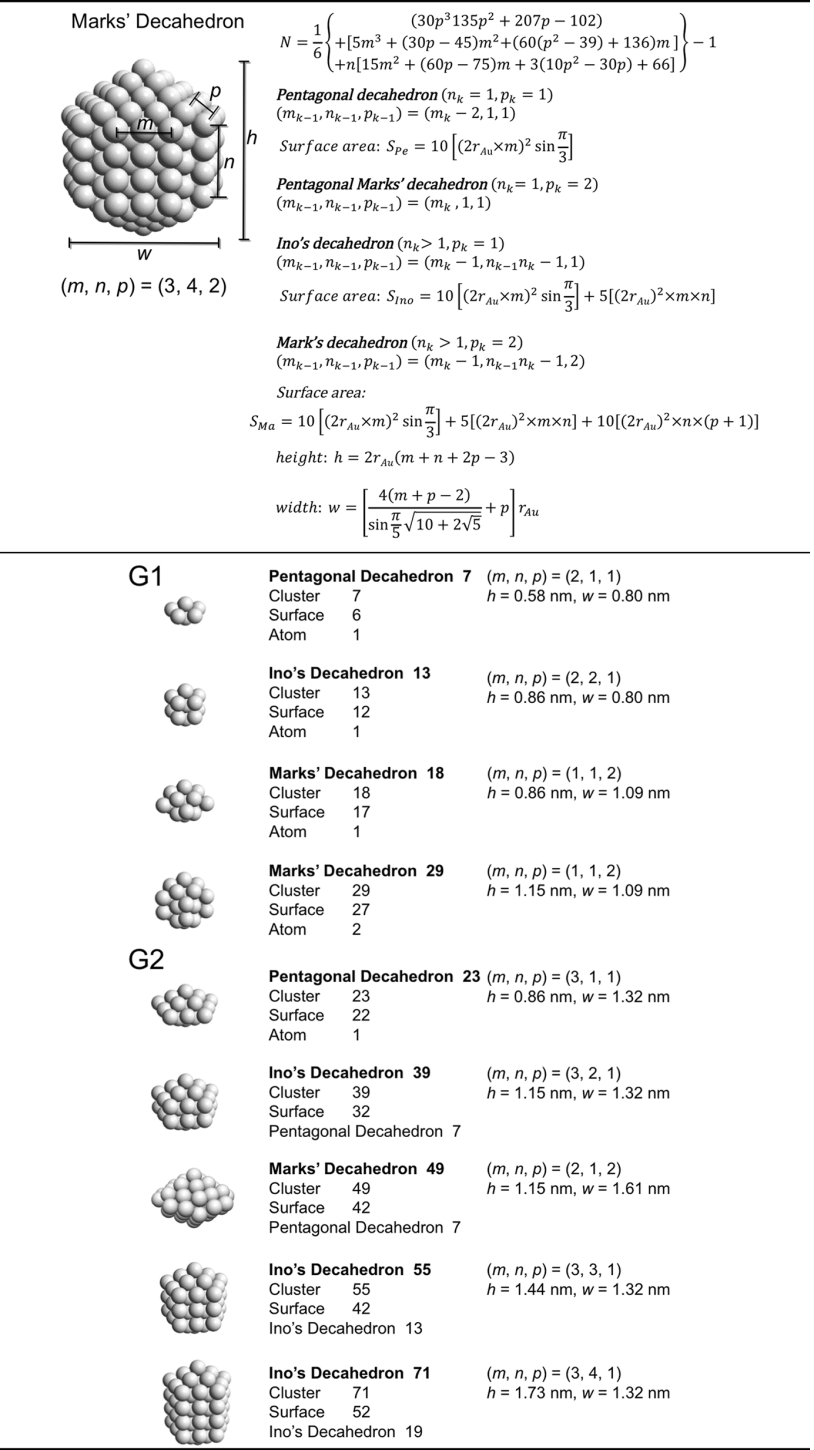

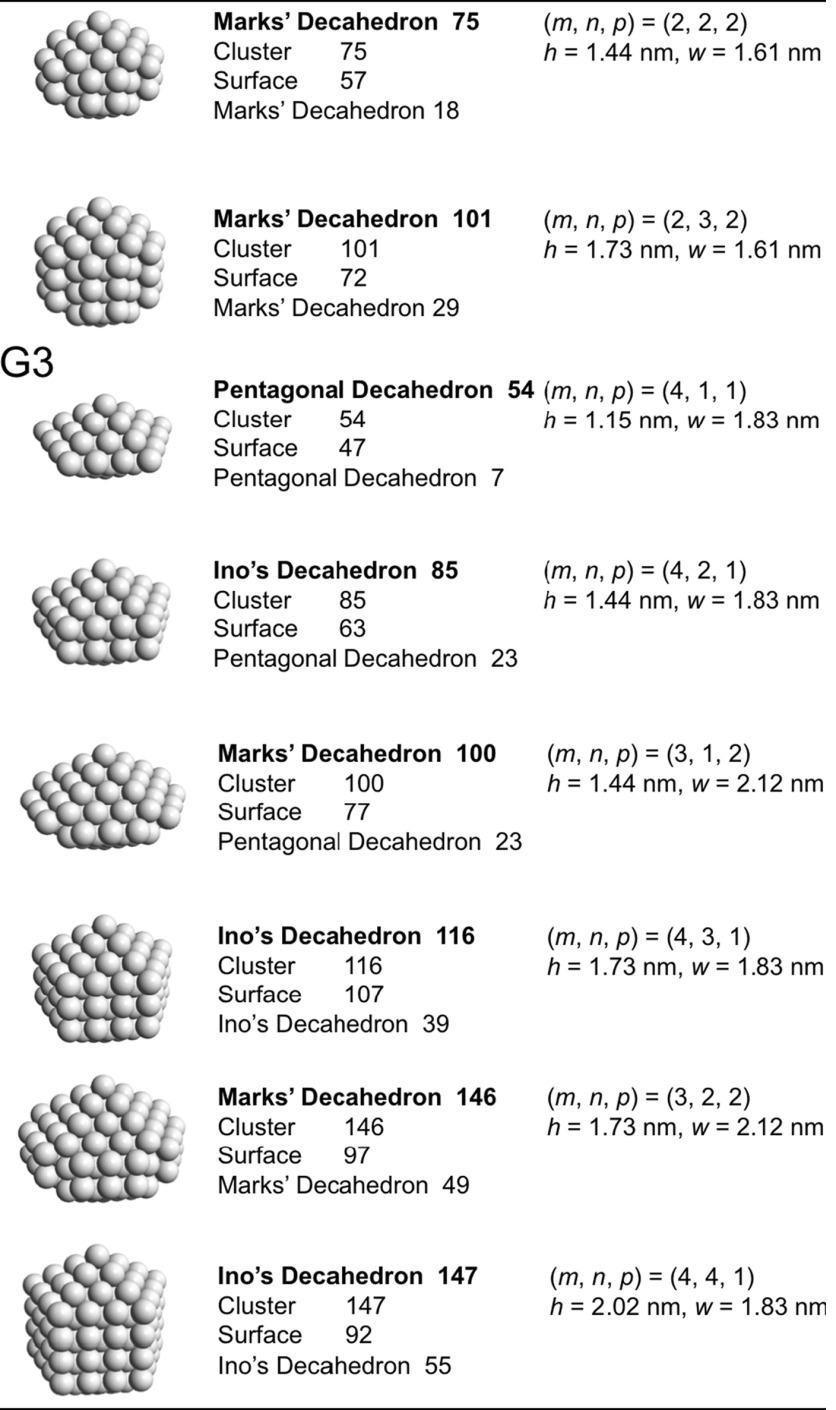

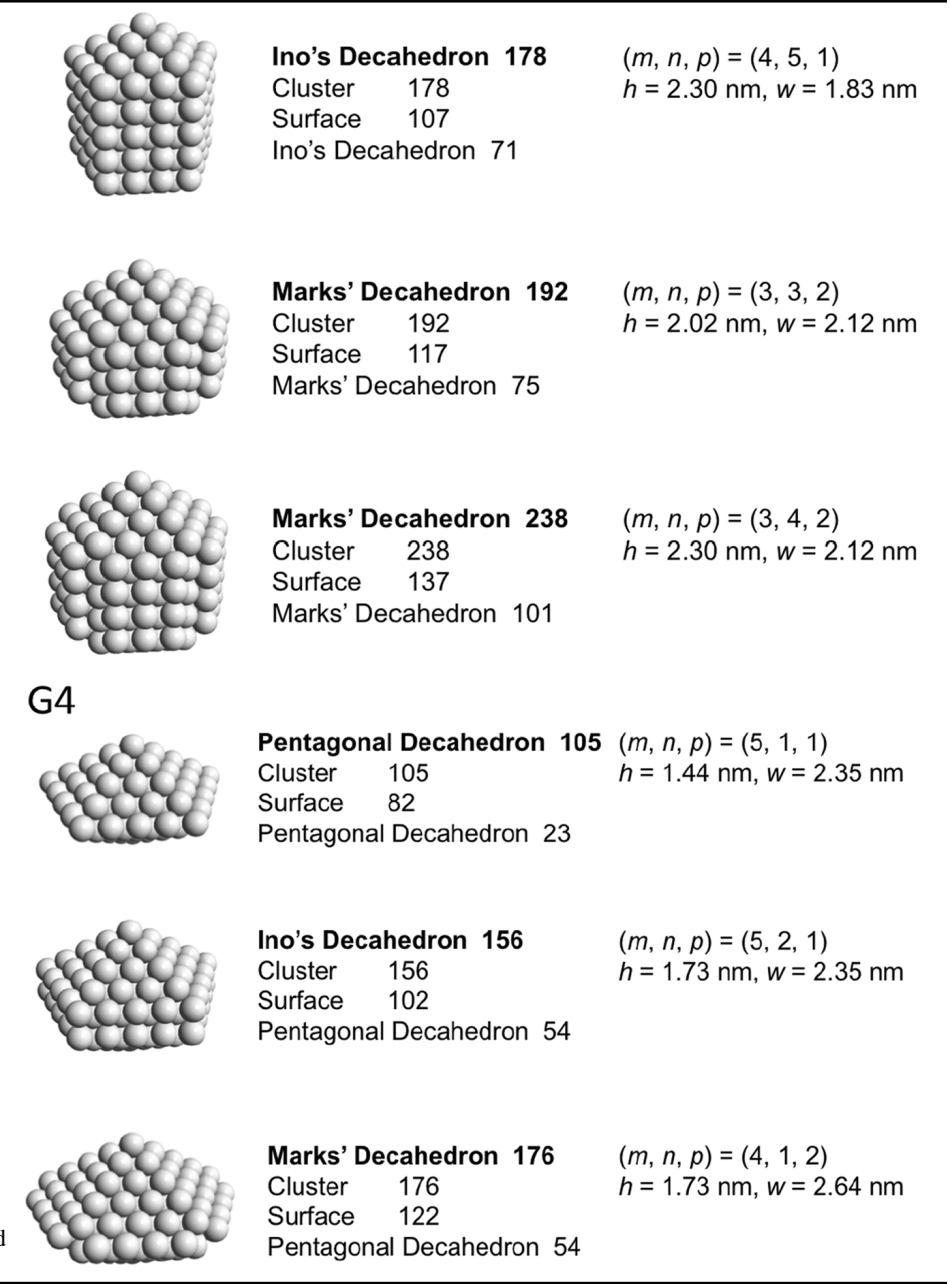
List of generations (G1 to G4) of pentagonal decahedra, Ino’s decahedra, and Marks’ decahedra (number of atoms in cluster, on the surface, parent cluster, height, and width)

The parameters and equations used are shown at the top of the table

A more condensed view of these values is given in Table 3, also providing additional generation 4 (G4) clusters. The number of gold atoms calculated assuming an ellipsoidal shape (*N*_*e*_) of the overall cluster is given in Eq. 5 (*h*, *r*_Au_, and *w* are defined in Table 2).

**Table 3:** List of clusters with decahedral shape, including regular, Ino’s, and Marks’ decahedra

Decahedron	G	*m*	*n*	*p*	Cluster	Surface	Inner	*h*/nm	*w*/nm	*N*_*e*_	*N*_*e*_/*N*
Pentagonal decahedron 7	1	2	1	1	7	6	1	0.58	0.80	3.5	0.50
Ino’s decahedron 13	1	2	2	1	13	12	1	0.86	0.80	5.9	0.45
Marks’ decahedron 18	1	1	1	2	18	17	1	0.86	1.09	14.3	0.79
Marks’ decahedron 29	1	1	2	2	29	27	2	1.15	1.09	20.0	0.69
Pentagonal decahedron 23	2	3	1	1	23	22	1	0.86	1.32	23.5	1.02
Ino’s decahedron 39	2	3	2	1	39	32	7	1.15	1.32	33.0	0.85
Marks’ decahedron 49	2	2	1	2	49	42	7	1.15	1.61	54.0	1.10
Ino’s decahedron 55	2	3	3	1	55	42	13	1.44	1.32	42.4	0.77
Ino’s decahedron 71	2	3	4	1	71	52	19	1.73	1.32	51.8	0.73
Marks’ decahedron 75	2	2	2	2	75	57	18	1.44	1.61	69.4	0.93
Marks’ decahedron 101	2	2	3	2	101	72	29	1.73	1.61	84.8	0.84
Pentagonal decahedron 54	3	4	1	1	54	47	7	1.15	1.83	74.2	1.37
Ino’s decahedron 85	3	4	2	1	85	62	23	1.44	1.83	95.4	1.12
Marks’ decahedron 100	3	3	1	2	100	77	23	1.44	2.12	134.2	1.34
Ino’s decahedron 116	3	4	3	1	116	77	39	1.73	1.83	116.5	1.00
Marks’ decahedron 146	3	3	2	2	146	97	49	1.73	2.12	164.0	1.12
Ino’s decahedron 147	3	4	4	1	147	92	55	2.02	1.83	137.7	0.94
Ino’s decahedron 178	3	4	5	1	178	107	71	2.30	1.83	158.9	0.89
Marks’ decahedron 192	3	3	3	2	192	117	75	2.02	2.12	193.8	1.01
Marks’ decahedron 238	3	3	4	2	238	137	101	2.30	2.12	223.7	0.94
Pentagonal decahedron 105	4	5	1	1	105	82	23	1.44	2.35	169.5	1.61
Ino’s decahedron 156	4	5	2	1	156	102	54	1.73	2.35	207.2	1.33
Marks’ decahedron 176	4	4	1	2	176	91	85	1.73	2.64	269.1	1.53
Ino’s decahedron 207	4	5	3	1	207	122	85	2.02	2.35	244.9	1.18
Ino’s decahedron 258	4	5	4	1	258	142	116	2.30	2.35	282.5	1.10
Ino’s decahedron 309	4	5	5	1	309	162	147	2.59	2.35	320.2	1.04
Marks’ decahedron 247	4	4	2	2	247	147	100	2.02	2.64	318.1	1.29
Marks’ decahedron 318	4	4	3	2	318	172	146	2.30	2.64	367.0	1.15
Marks’ decahedron 389	4	4	4	2	389	197	192	2.59	2.64	415.9	1.07

*G* generation, *m*, *n*, and *p* are defined in Table 2, and the inner cluster is the parent cluster

5 $$ N_{e} = \frac{4\pi }{{3V_{\text{Au}} }}\frac{{h - r_{\text{Au}} }}{2}\left( {\frac{{w - r_{\text{Au}} }}{2}} \right)^{2} $$

Divided by the precise number of gold atoms in the cluster, the ratio of *N*_*e*_/*N* shows how close an elliptical particle shape assumption would be as the size of the cluster increases, especially in the absence of high-resolution TEM images or X-ray diffraction data that would allow the experimentalist to deduce the exact particle shape and composition.

### Archimedean icosahedra model

Table 4 lists the same information for Archimedean icosahedra starting with the smallest, first-generation (G1) Au_13_ cluster. Among them, we also find several of the magic-sized gold nanoclusters with icosahedral shape such as Au_13_, Au_55_, Au_147_, among others, as shown in Fig. 9.

**Table 4:**
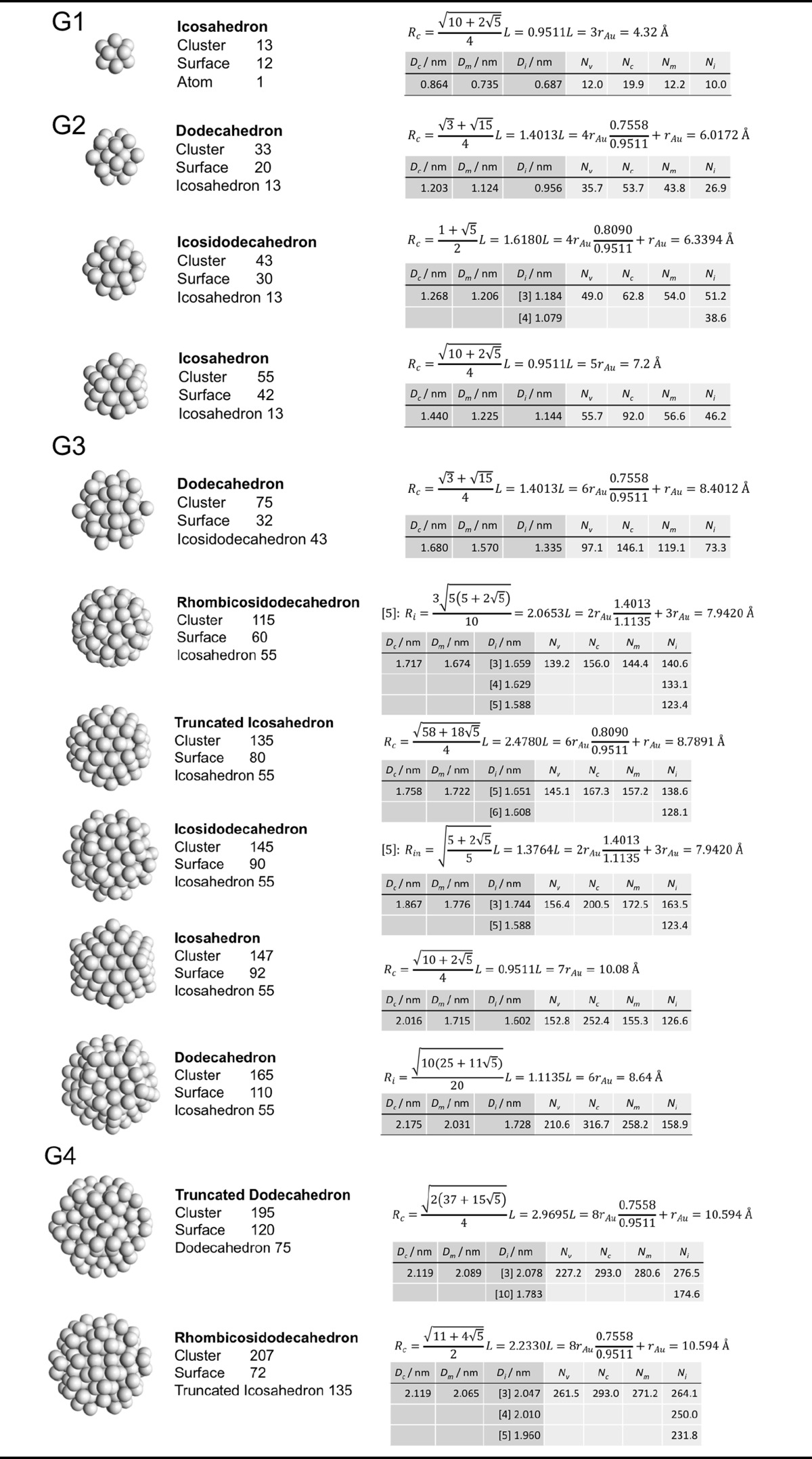

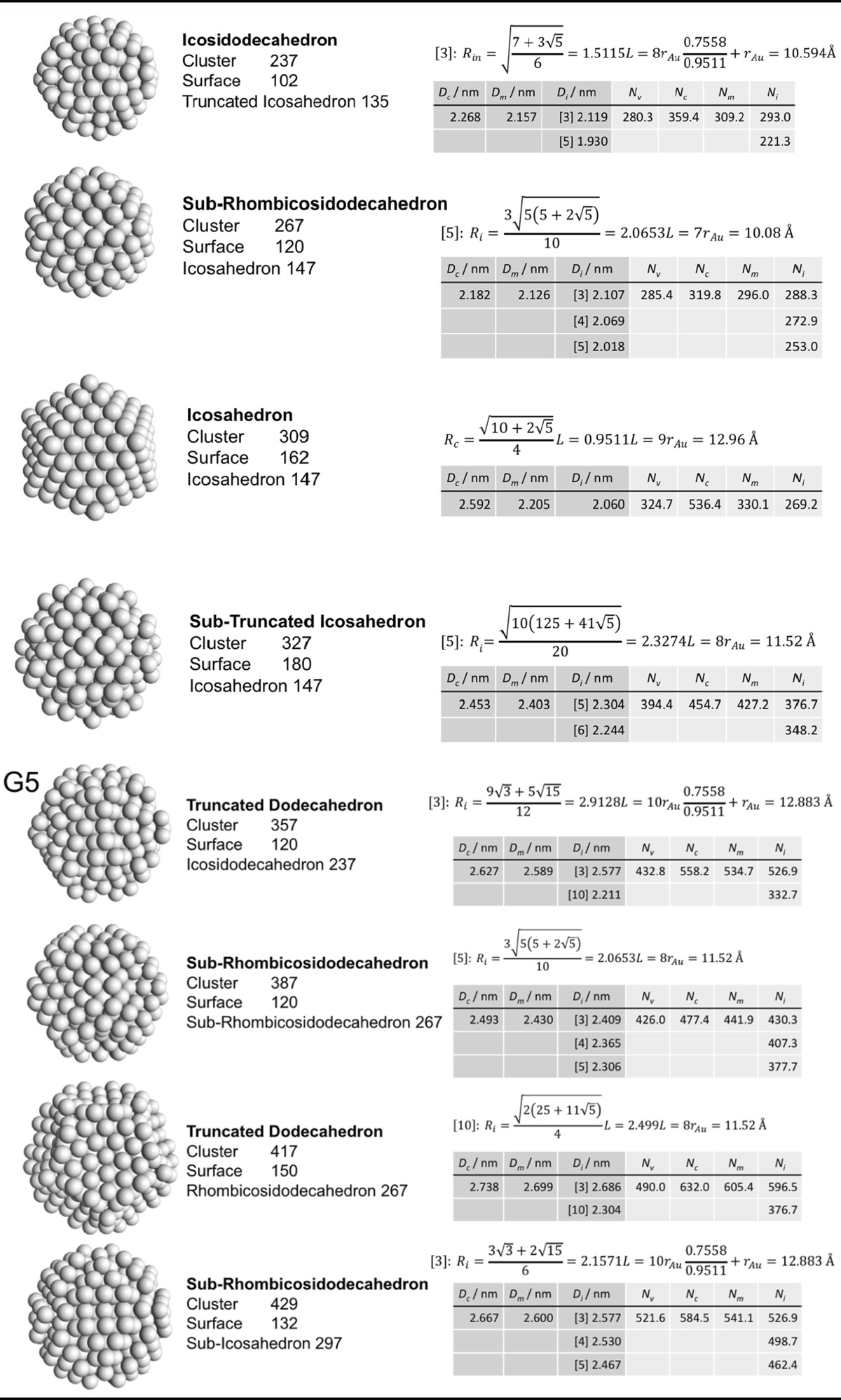

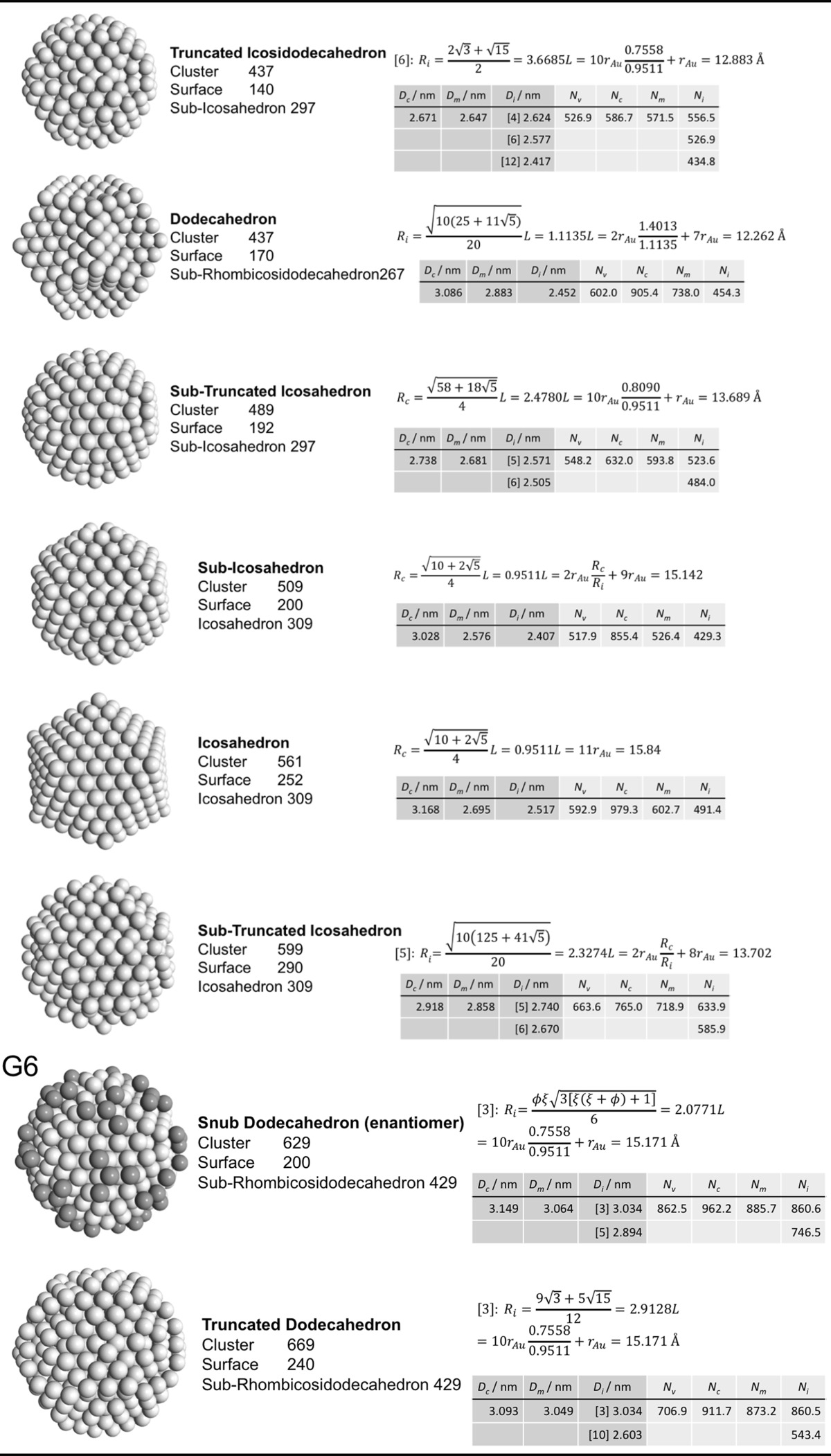

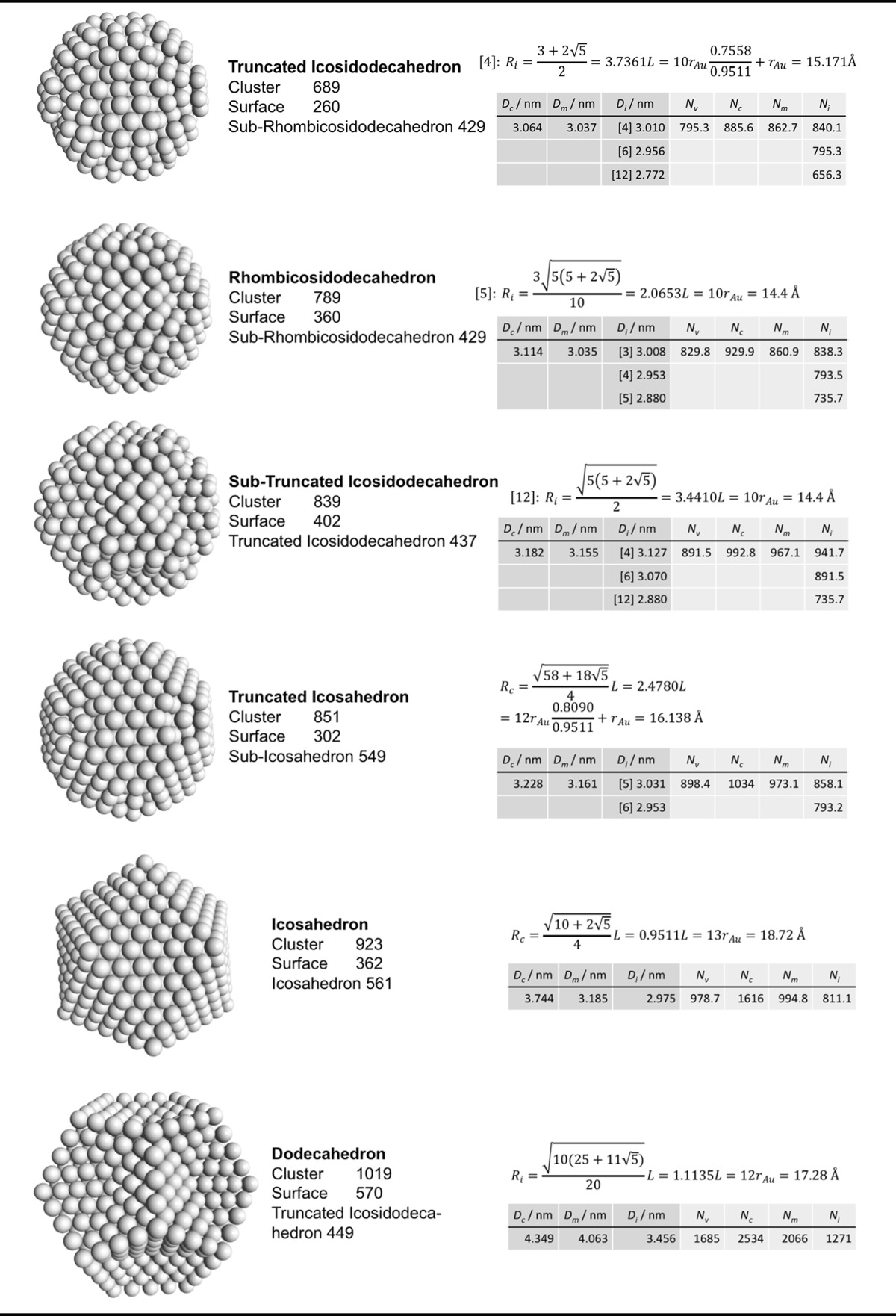
List of generations (G1 to G6) of Archimedean icosahedra (number of atoms in cluster, on the surface, parent (inner) cluster, height, and width

The parameters and equations used are shown for each cluster. *L* is the edge length of the polyhedron, *D*_c_, *D*_m_, and *D*_i_ are the circumscribed, midscribed, and inscribed diameter (*R*_c_ is the circumscribed radius), *N*_*v*_, *N*_c_, *N*_*m*_, and *N*_i_ are the number of gold atoms calculated from the volume of a polyhedron, the volume of a sphere with circumscribed diameter, the volume of a sphere with midscribed diameter, and the volume of a sphere with inscribed diameter, respectively. Numbers in square brackets for the inscribed diameter *D*_i_ or radius *R*_i_ denote faces of the Archimedean icosahedra, e.g., [3] for triangle, [5] for pentagon

The ratio of the number of gold atoms between calculated and ideal cluster in these Archimedean icosahedra (Table S3, Section S3) shows how the quasi-spherical model (using the diameter of the circumscribed (*N*_c_), midscribed (*N*_m_) or inscribed diameter (*N*_i_) sphere), or using the number of gold atoms deduced from a polyhedral model (*N*_*v*_) deviates from the correct number of gold atoms for these clusters.

### Archimedean cube model

Table 5 finally shows a list of Archimedean cubes from generation 1 to 6 (G1–G6). Again, the ratio of the number of gold atoms between calculated and ideal cluster in these Archimedean cubes (Table S4, Section S4) shows how quasi-spherical models (using the diameter of the circumscribed (*N*_c_), midscribed (*N*_m_) or inscribed diameter (*N*_i_) sphere), or using the number of gold atoms deduced from a polyhedral model (*N*_*v*_) deviate from the correct number of atoms.

**Table 5:**
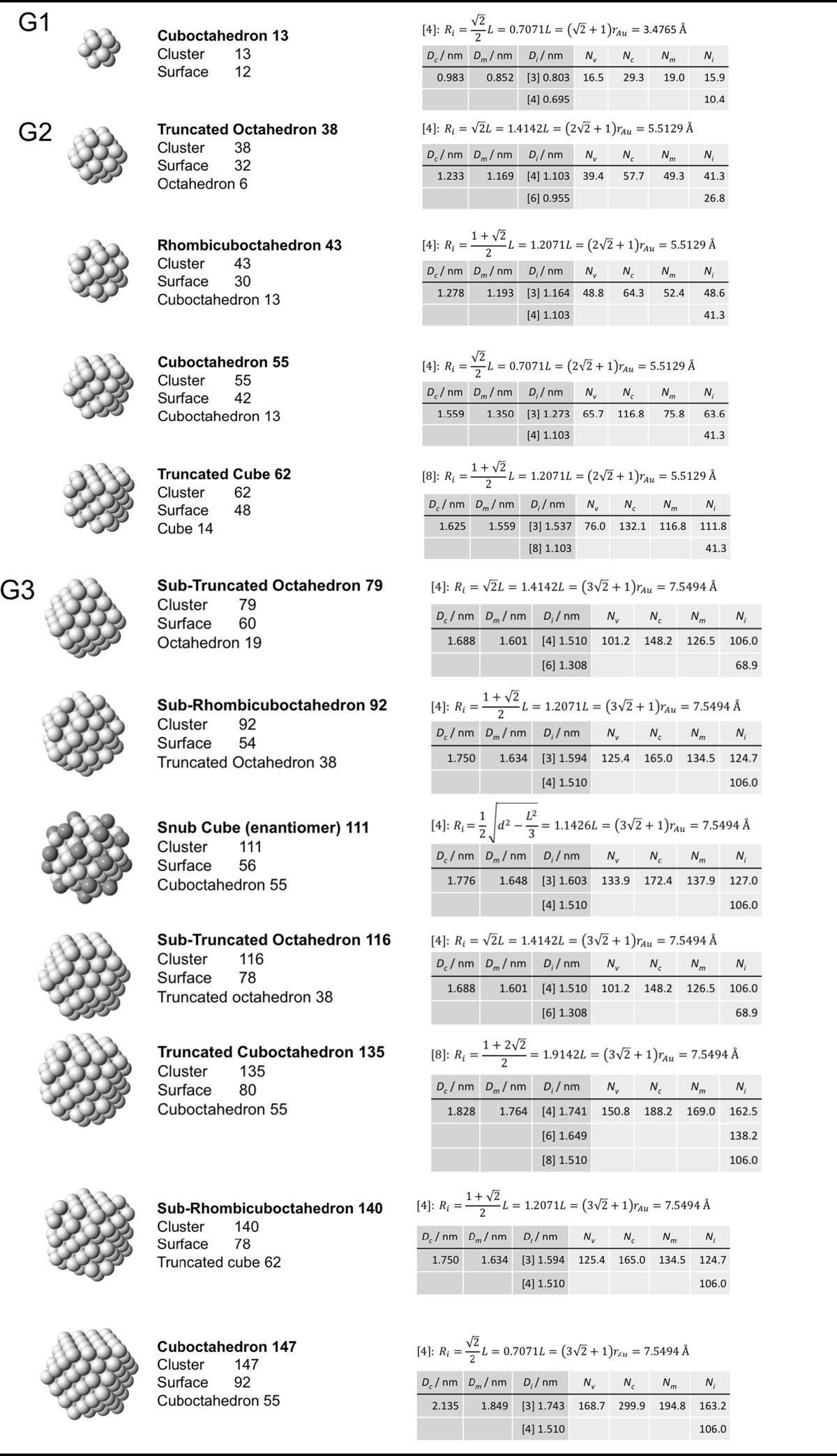

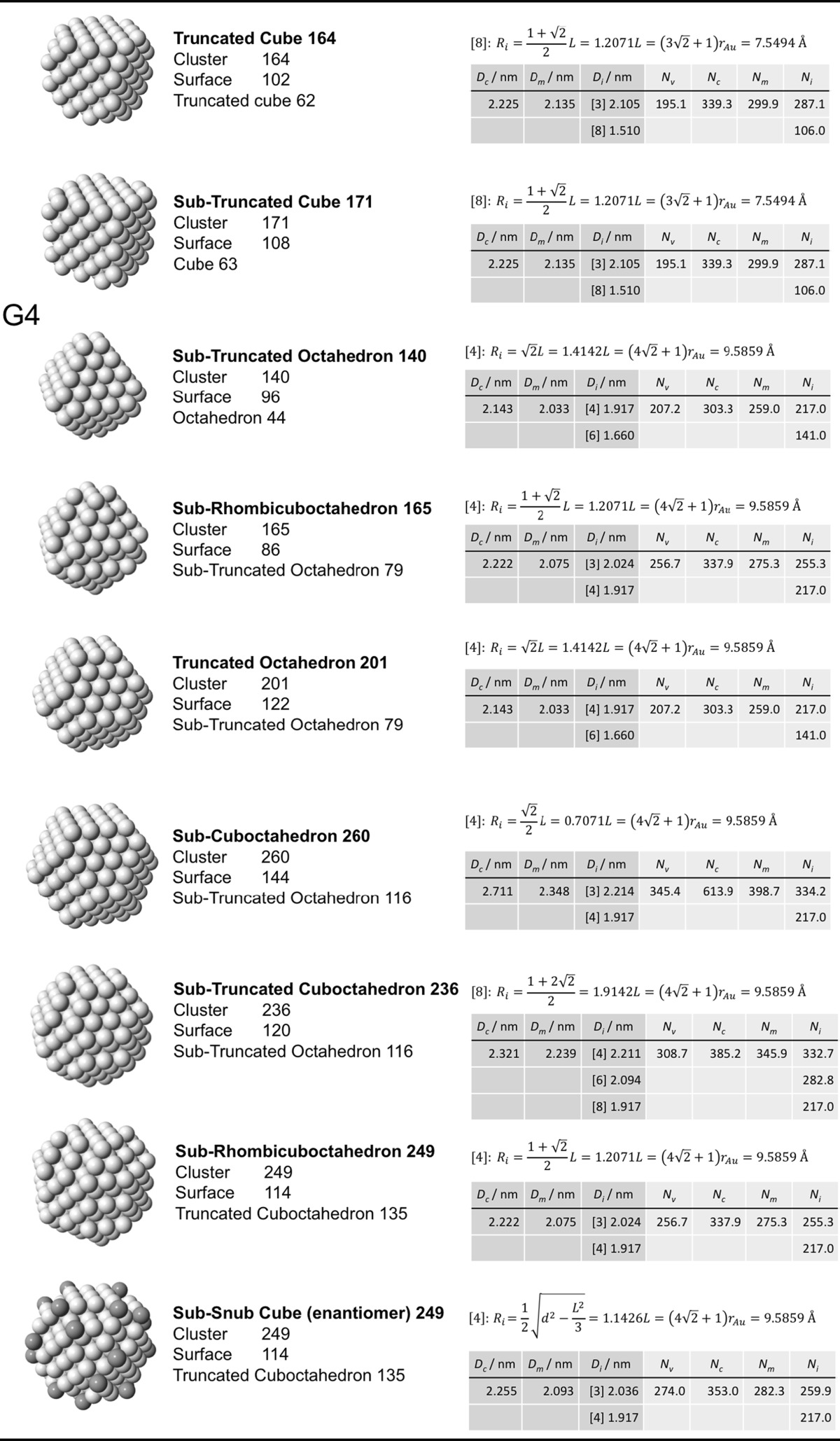

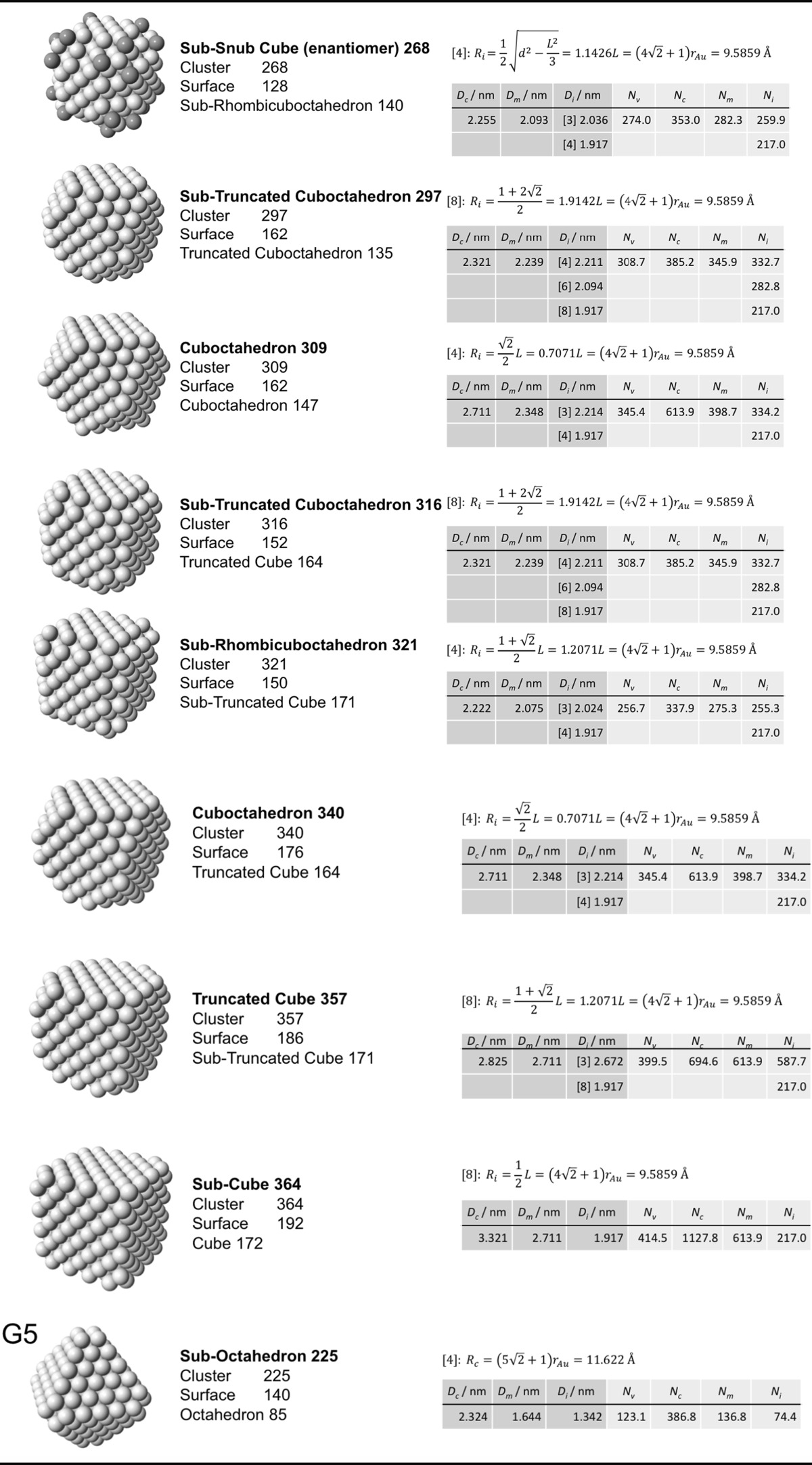

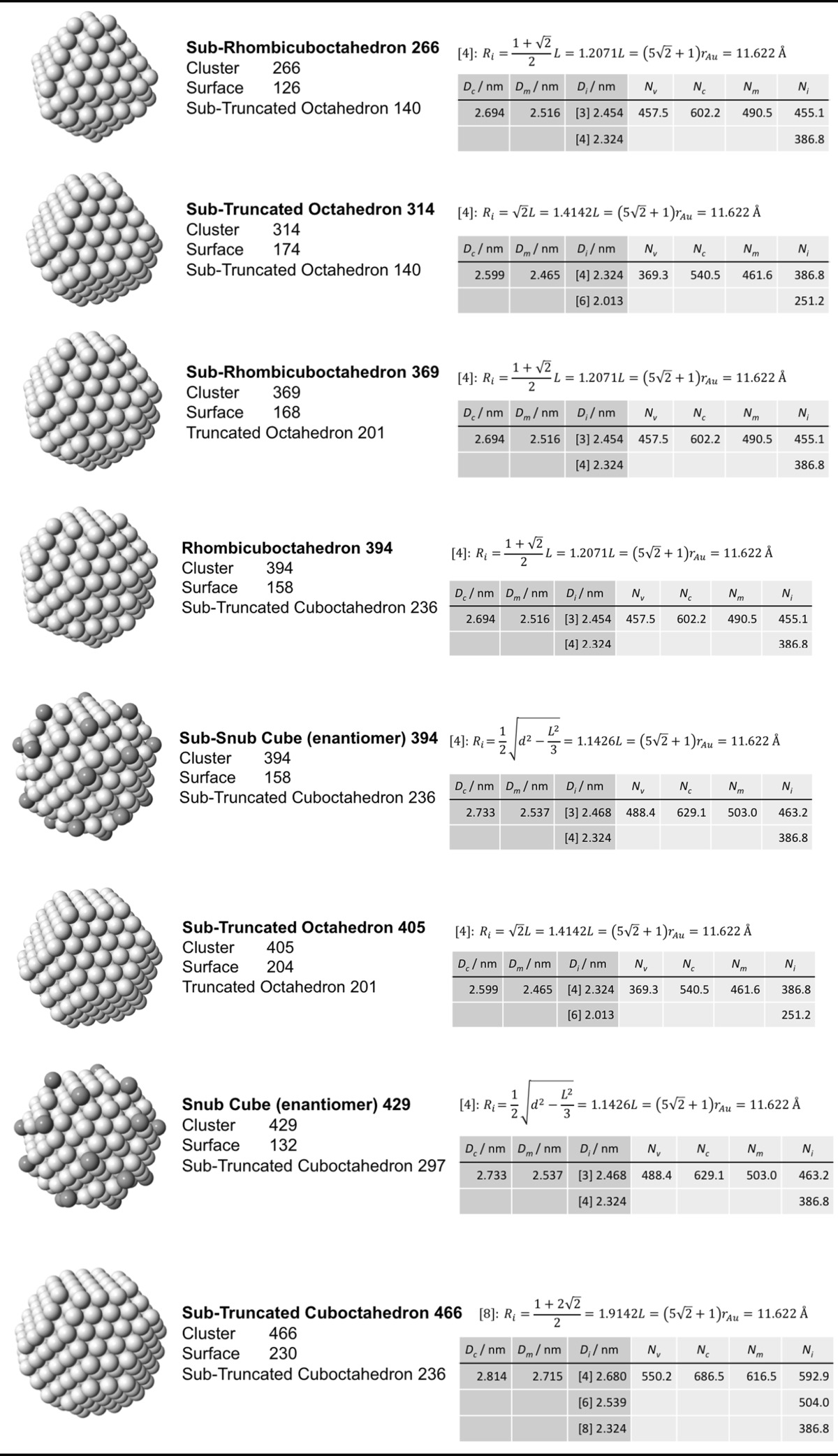

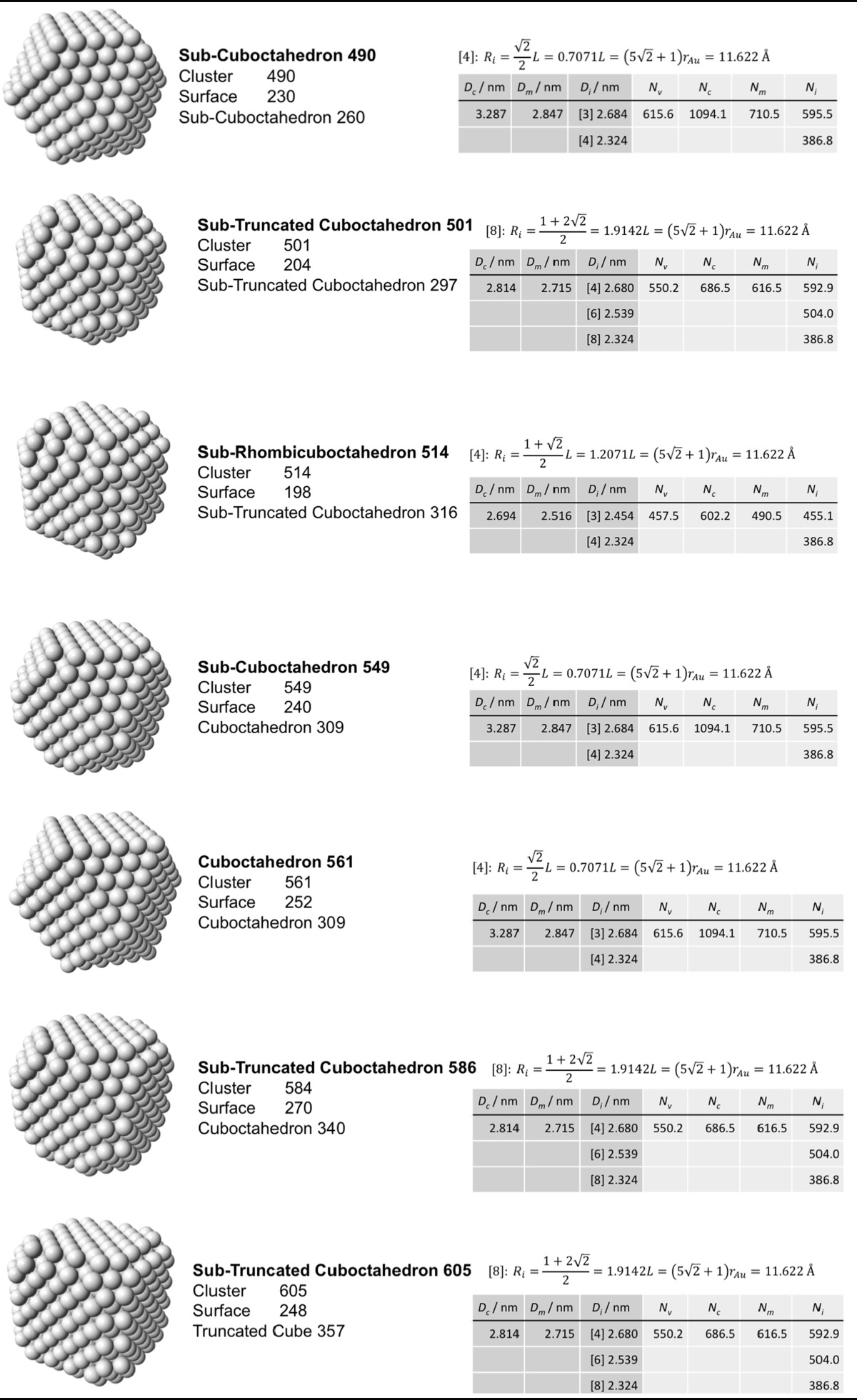

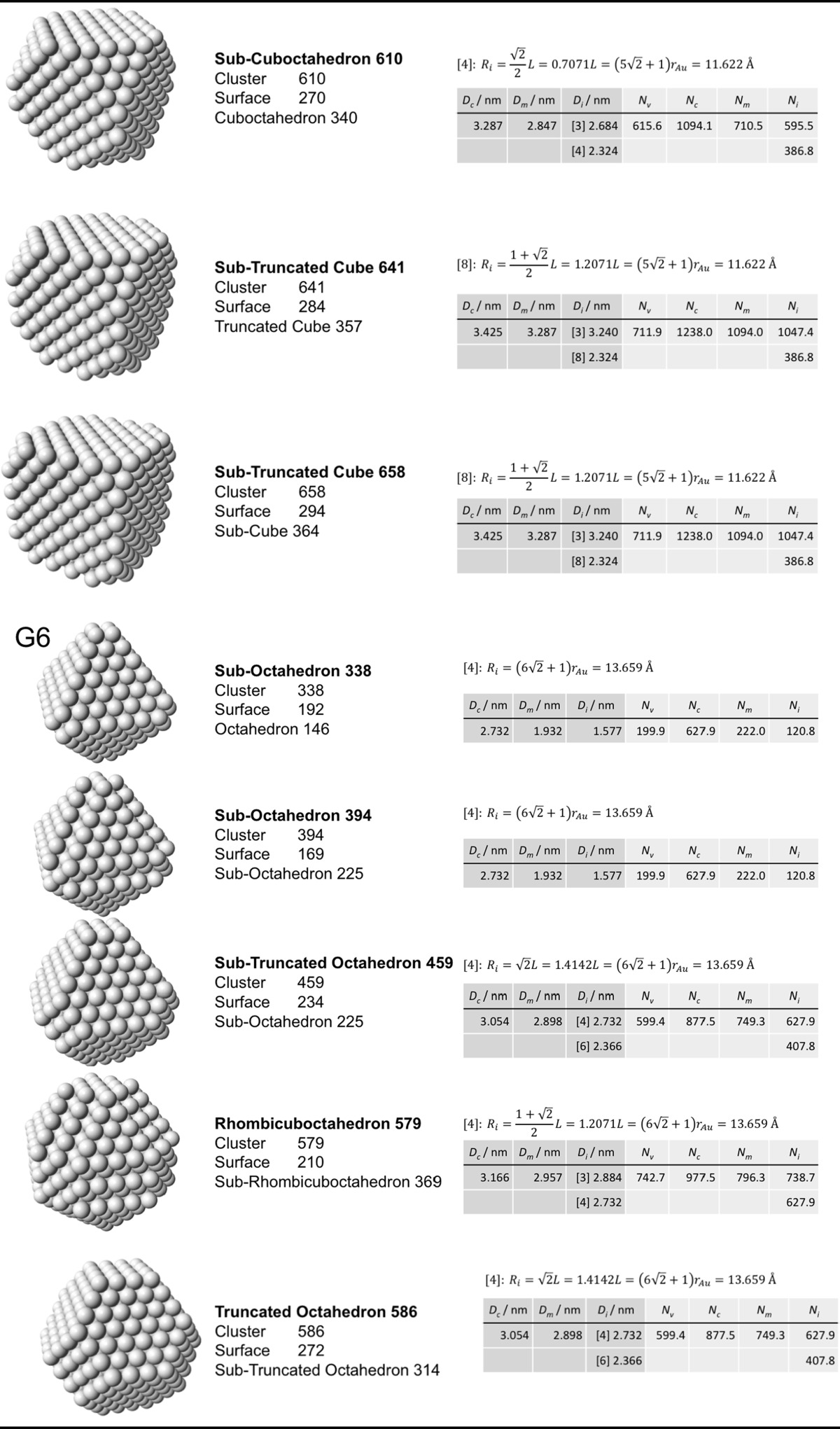

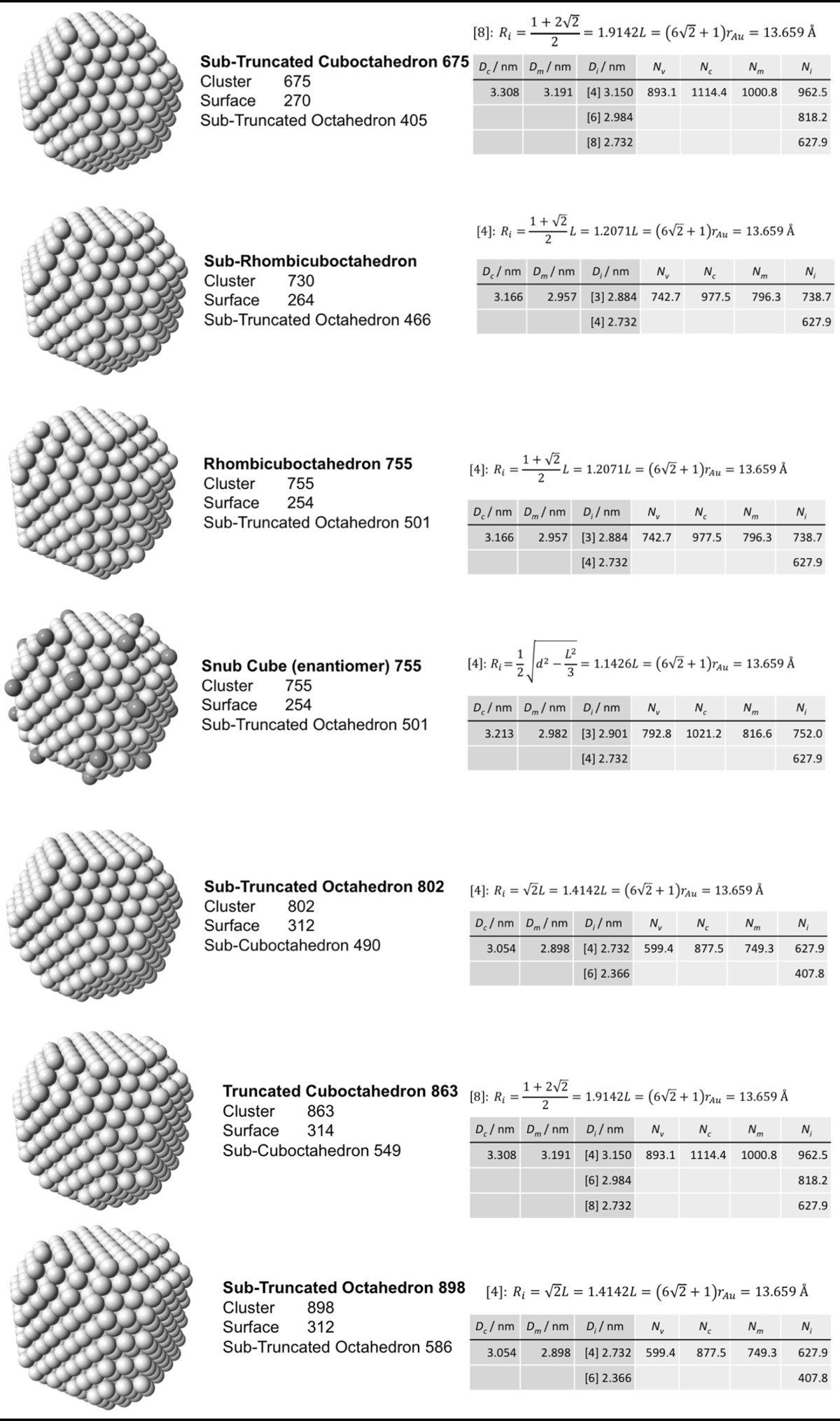

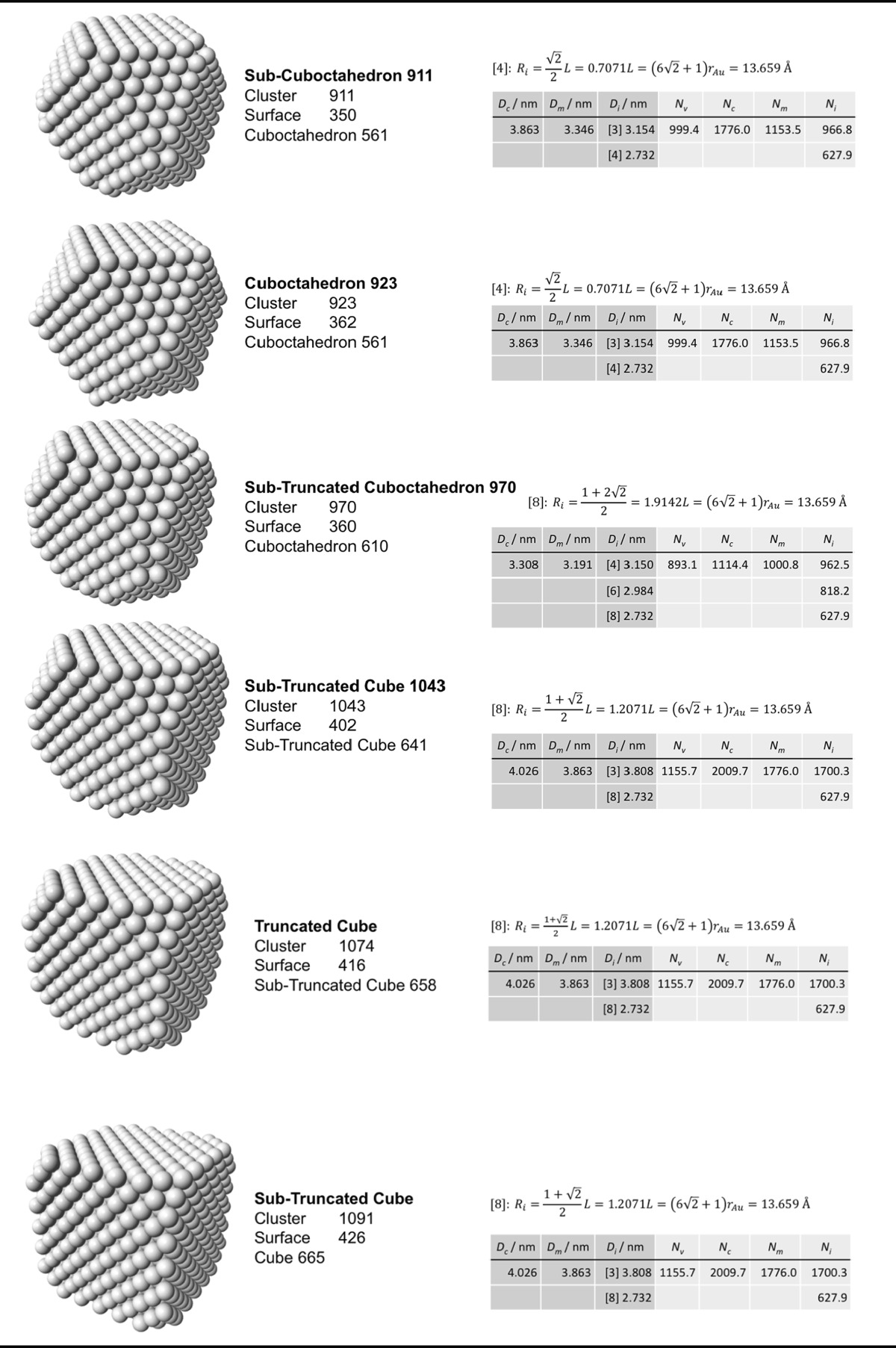
List of generations (G1 to G6) of Archimedean cubes (number of atoms in cluster, on the surface, parent (inner) cluster, height, and width

The parameters and equations used are shown for each cluster. *L* is the edge length of the polyhedron, *D*_c_, *D*_m_, and *D*_i_ are the circumscribed, midscribed, and inscribed diameter (*R*_c_ is the circumscribed radius), *N*_*v*_, *N*_c_, *N*_*m*_, and *N*_i_ are the number of gold atoms calculated from the volume of a polyhedron, the volume of a sphere with circumscribed diameter, the volume of a sphere with midscribed diameter, and the volume of a sphere with inscribed diameter, respectively. Numbers in square brackets for the inscribed diameter *D*_i_ or radius *R*_i_ denote faces of polygons, e.g., [3] for triangle, [5] for pentagon

### Other models: defects, shell structures, and “staples”

Thus far, we have provided calculations and models for regular polyhedral shapes of gold nanoparticles. However, these models do not include specifics of shell structures (the outer layer of gold atoms involved in thiolate ligand binding), but we can estimate the number of gold-thiolate “staple” and bridge motifs by calculating the ligand density at the gold nanoparticle surface.

Particularly, the cores of chiral gold nanoparticles consist of non-regular polyhedral structures and usually exhibit defects in their crystal structures (Chen et al. 2015; Dolamic et al. 2012; Kimura et al. 2009; Levi-Kalisman et al. 2011; Lopez-Acevedo et al. 2010; Pei et al. 2009; Pelayo et al. 2015; Qian and Jin 2009; Takagi et al. 2015; Tlahuice-Flores et al. 2013a, b; Weissker et al. 2014; Zeng et al. 2015). Such non-regular polyhedral as well as defect structures of nanoparticle cores are generally the origin for the observed nanoparticle chirality. The core structures of several prominent chiral gold nanoparticles are summarized in Table 6.

**Table 6:** Core structures of chiral gold nanoparticles

Chiral gold nanoparticle	Core		References
	Number of core atoms	Structure	
Au_15_(S–CH_3_)_13_	4	Regular tetrahedron^#^	Tlahuice-Flores et al. (2013b)
Au_18_(S–C_6_H_11_)_14_	8	Continuous two octahedron	Pelayo et al. (2015)
Au_20_(S–Ph-*t*-Bu)_16_	7	Coupled two tetrahedron	Pelayo et al. (2015)
Au_20_(E-R_1_)_16_^a^	8	Continuous three tetrahedron	Pei et al. (2009), Takagi et al. (2015)
Au_20_(PP_3_)_4_C_l4_	20	Icosahedron + 7 atoms	Pelayo et al. (2015)
[Au_20_(PPhpy_2_)_10_Cl_4_]Cl_2_	20	Snub cube like	Pelayo et al. (2015)
Au_23_(S–C_6_H_11_)_16_	13	Regular cuboctahedron^#^	Pelayo et al. (2015)
Au_24_(E–R_1_)_20_^a^	8	Continuous three tetrahedron	Pei et al. (2009); Takagi et al. (2015)
Au_24_(S–CH_2_Ph-*t*-Bu)_20_	8	Cube + two tetrahedron	Pelayo et al. (2015)
Au_24_(S-adamantane)_16_	13	Regular cuboctahedron^#^	Pelayo et al. (2015)
[Au_25_(S–CH_2_CH_2_Ph)_18_]^−^	13	Regular icosahedron^#^	Pelayo et al. (2015)
Au_28_(S-Ph-*t*-Bu)_20_	20	Continuous two icosahedron	Pelayo et al. (2015)
Au_30_S(S-*t*-Bu)_18_	20	Continuous two cuboctahedron	Pelayo et al. (2015)
Au_36_(S–Bu)_24_	28	Icosahedron + 15 atoms	Pelayo et al. (2015)
Au_36_(S–CH_2_Ph-*t*-Bu)_8_Cl_20_	14	Decahedron with defects	Pelayo et al. (2015)
Au_38_(PET)_24_	23	Continuous two icosahedron	Pelayo et al. (2015)
Au_38_(PET)_24_	23	Icosahedron + dodecahedron	Pelayo et al. (2015)
Au_38_(S–CH_2_CH_2_Ph)_24_	24	Coupled two icosahedrons	Dolamic et al. (2012)
Au_38_(S–R_2_)_24_^b^	24	Coupled two icosahedrons	Dolamic et al. (2012)
Au_40_(*o*-MBT)_24_	25	Rhombicuboctahedron + some atoms	Pelayo et al. (2015)
Au_40_(S–CH_3_)_24_	26	Coupled two icosahedrons	Pelayo et al. (2015)
Au_44_(SCH_3_)_28_	26	Decahedron + some atoms	Pelayo et al. (2015)
Au_52_(S–Ph-*t*-Bu)_32_	32	Marks’ decahedron with defects	Pelayo et al. (2015)
Au_68_(SH)_34_	15	Cuboctahedron + some atoms	Pelayo et al. (2015)
Au_68_(3-MBA)_50_	50	Icosidodecahedron with defect	Pelayo et al. (2015)
Au_102_(*p*-MBA)_44_	79	Rhombicosidodecahedron with defects or Ino’s, Marks’ decahedron with defects	Levi-Kalisman et al. (2011), Pelayo et al. (2015)
Au_130_(*p*-MBT)_50_	105	Marks’ decahedron with defects	Pelayo et al. (2015), Zeng et al. (2015)
Au_133_(S–Ph-*p*–*t*-Bu)_52_	107	Rhombicosidodecahedron with defects	Pelayo et al. (2015), Zeng et al. (2015)
Au_144_(S–R_3_)_60_^c^	114	Rhombicosidodecahedron with defects	Pelayo et al. (2015), Qian and Jin (2009), Weissker et al. (2014)
Au_144_Cl_60_	114	Rhombicosidodecahedron with defects	Pelayo et al. (2015), Tlahuice-Flores et al. (2013a)

^a^E = Se, S; R_1_ = Ph, CH_3_

^b^R_2_ = CH_3_, C_6_H_13_, C_12_H_25_

^c^R_3_ = CH_3_, CH_2_CH_2_Ph

^#^Gold nanoparticles with regular polyhedra cores

**Fig. 10:**
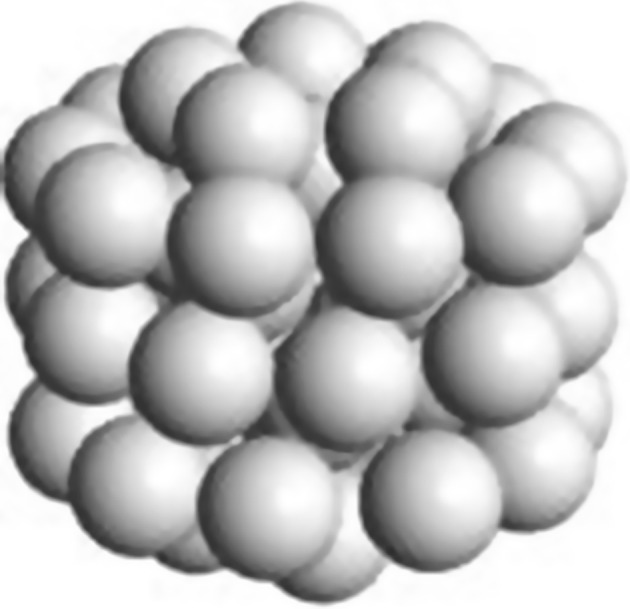
Model for the chiral Au_53_ cluster

Particularly, the cores of the smaller chiral gold nanoparticles are composed of connected regular polyhedra such as continuous tetrahedra and/or icosahedra. The core of Au_68_(3-MBA)_50_ features 50 gold atoms formed from an Archimedean icosahedral structure (icosidodecahedron) with defects (Pelayo et al. 2015). Similarly, the cores of both Au_102_(*p*-MBA)_32_ and Au_133_(S-Ph-*p*–*t*-Bu)_52_ were formed from rhombicosidodecahedron also with defects (Chen et al. 2015; Levi-Kalisman et al. 2011; Pelayo et al. 2015; Zeng et al. 2015). The core structure of Au_68_(3-MBA)_50_ appears to be most closely related either to an Ino’s decahedron with defects or to a non-regular polyhedral Au_53_ as shown in Fig. 10 (Pelayo et al. 2015). Several other chiral gold nanoparticles have regular polyhedra cores (entries highlighted by # in Table 6). For example, the core of Au_144_(S-R_3_)_60_ formed from Au_114_ (Qian and Jin 2009; Tlahuice-Flores et al. 2013a) finds its best match in the Archimedean icosahedra model as rhombicosidodecahedron Au_115_ in Table 4.

To estimate the number of gold atoms on the surface we have to calculate the ligand density (*ρ*_L_). Table 7 shows the surface area of the nanoparticle core (*S*_c_) as well as the ligand density for gold nanoparticles. The ligand density of [Au_25_(S–CH_2_CH_2_Ph)_18_]^−^ and Au_144_(S–CH_3_)_60_ with Archimedean icosahedra cores, calculated using simple Eq. 6 (*N*_L_ is the number of thiolate ligands), is close to 15 Å^2^, which is equal to the maximum packing density of thiolates on gold nanoparticle (Kimura et al. 2009).

6 $$ \rho_{\text{L}} = \frac{{S_{\text{c}} }}{{N_{\text{L}} }} $$

**Table 7:** Examples of calculated surface areas for selected nanoparticle cores and corresponding densities of thiolate ligands (from Eq. 6 and Table 2)

Gold nanoparticle	Core	*S*_c_/Å^2^	*N*_L_	*ρ*_L_/Å^2^
Au_24_(S-adamantane)_16_	Cuboctahedron 13 [12 + 1]	228.8	11^a^	20.8
[Au_25_(S–CH_2_CH_2_Ph)_18_]^−^	Icosahedron 13 [12 + 1]	178.7	12^a^	14.89
Au_54_(S–C_18_H_37_)_30_ Au_55_(S–C_18_H_37_)_31_	Ino’s decahedron 39 - 2 (*m*, *n*, *p* = 3, 2, 1)	327.9	18^a^	18.2
Au_144_(S–CH_3_)_60_	Rhombicosidodecahedron 114 [60 + 54]	877.0	60	14.6
Au_187_(S–C_12_H_25_)_68_	Truncated cuboctahedron 135 [80 + 55]	960.6	68	14.1
	Cuboctahedron 147 [92 + 55]	1079		15.9
	Marks’ decahedron 153 (*m*, *n*, *p* = 2, 5, 2)	1127		16.6
Au_333_(S-CH_2_CH_2_Ph)_79_	Sub-truncated octahedron 314 [198 + 116]	1809	79	22.9
	Cuboctahedron 309 [162 + 147]	1739		22.0
	Sub-truncated cuboctahedron 297 [162 + 135]	1549		19.6
	Ino’s decahedron 309 (*m*, *n*, *p* = 5, 5, 1)	1256		15.9
	Marks’ decahedron 318 (*m*, *n*, *p* = 4, 3, 2)	1161		14.1

^a^The number of ligands on the core surface is lower than the total number of ligands on the gold nanoparticle, because gold nanoparticles are formed by –S–Au–S–Au–S–“staple” motifs. In this case, the number of gold atom in the shell is substituted for the number of ligands on the core surface (*N*_L_)

The Au_144_(S-CH_3_)_60_ cluster reported by Jin et al. (Qian and Jin 2009) has 30–S–Au–S–“staple” motifs with 30 gold and 60 sulfur atoms within the shell structure (Weissker et al. 2014). Using the same approach, we calculated that the ligand density of the Au_24_(S-adamantane)_16_ cluster(Pelayo et al. 2015) with an Archimedean cube core was 19 Å^2^, similar to the surface area of thiols calculated for planar gold surfaces (i.e., self-assembled monolayers on gold, SAMs (Love et al. 2005)) at 21 Å^2^. This suggests that the surface of cores with Archimedean cube structure would act more like a bulk gold surface than cores with Archimedean icosahedra shape that are more faceted, which makes sense.

The cores of Au_54_(S–C_18_H_37_)_30_ and Au_55_(S–C_18_H_37_)_31_ were formed from Ino’s decahedron 39 (Negishi et al. 2012; Tsunoyama et al. 2010). The ligand density of Ino’s decahedron 39 (*ρ*_L_ = 18.2 Å^2^) is situated between the thiol ligand density of Archimedean icosahedra gold nanoparticles (*ρ*_L_ = 15 Å^2^) and flat gold surfaces (*ρ*_L_ = 21 Å^2^). The reason for this is that the decahedra surfaces are formed from a combination of icosahedral core (particle like) on triangular faces and cubic core (bulk like) on rectangular faces.

The core structure of the Au_187_(S–C_12_H_25_)_68_^15^ has been elucidated by density functional theory (DFT) calculations as a Marks’ decahedron Au_153_ (Tlahuice-Flores 2015). It is conceivable that Au_187_(S-C_12_H_25_)_68_ clusters have either a truncated cuboctahedron 135 or a cuboctahedron 147 core structure considering the models listed in Table 5. The ligand densities of the Au_187_(S–C_12_H_25_)_68_ cluster were calculated for each possible core (Table 7).

The ligand densities of a Marks’ decahedral Au_153_, a truncated cuboctahedron 135, and a cuboctahedron 147 amount to 16.6, 14.1, and 15.9 Å^2^, respectively. The values of the ligand densities obtained for the truncated cuboctahedron 135 and the cuboctahedron 147 suggest that thiolate ligands are more tightly packed on these clusters than thiolates on gold SAMs (21 Å^2^). Thus, the core of the Au_187_(S–C_12_H_25_)_68_ should be neither a truncated cuboctahedron 135 nor a cuboctahedron 147, because the ligand density of a particle core with Archimedean cube structure should be closer to the surface area of thiolates on a flat gold SAM. Thus, the core structure of the Au_187_(S–C_12_H_25_)_68_ should be based on a Marks’ decahedral Au_153_ as determined by the authors experimentally.

Qian et al. (2012) reported on the core of a Au_333_(S-CH_2_CH_2_Ph)_79_ cluster formed from fcc Au_293_. We can suggest other possible core structures from the models summarized in the tables. The ligand densities of cores with Archimedean cube structure such as sub-truncated cuboctahedron 297, cuboctahedron 309, sub-truncated octahedron 314 were calculated to be 22 Å^2^, closely matching with the surface area of thiolates on gold SAMs. The ligand densities of cores with decahedral structure such as Ino’s decahedron 309 and Marks’ decahedron 318 are 15 Å^2^, which is close to that of the Au_187_(S–C_12_H_25_)_68_ cluster formed from a Marks’ decahedral Au_153_. The authors considered sub-truncated cuboctahedron 297 or cuboctahedron 309 as core structure of the Au_333_(S–CH_2_CH_2_Ph)_79_ cluster, but a sub-truncated octahedron 314, an Ino’s decahedron 309, and a Marks’ decahedron 318 should be reconsidered as the most likely core structures based on ligand density values. These examples show how the tables, calculations, and consideration of ligand densities can be used to determine the core structure of gold nanoparticles. The following part will provide a quick how-to guide.

### Using the tables

**Fig. 11:**
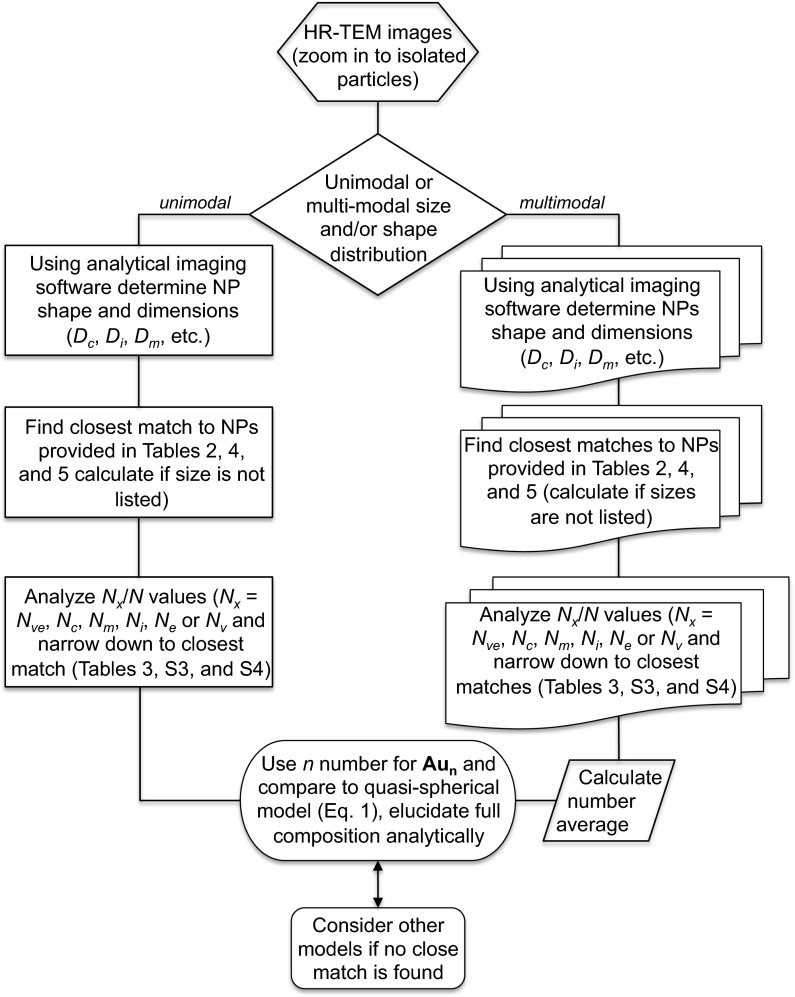
Flowchart diagram of the procedure to obtain the closest match in gold nanoparticle core composition from available experimental values obtained by TEM (ideally HR-TEM or TEM tomography) image analysis

Now that we have the tabulated data for the various models, it is time to put them to the test. First, we provide a point-by-point procedure how to use these tables for a given nanoparticle sample. Several examples can be found in the ESM (Section S5). Equipped with TEM images (even better high-resolution TEM images or TEM tomography data) that should allow the experimentalist to determine the shape(s) or closest match to one of the regular polyhedra, Archimedean icosahedra, Archimedean cubes, Ino’s or Marks’ decahedra, the following steps should lead to a close match between real and calculated core composition (a simplified flowchart of this procedure is shown in Fig. 11):

1. Zoom into the TEM image showing isolated, non-aggregated gold nanoparticles as much as possible without sacrificing (shape) resolution,
2. Using your imaging software (with analytical capabilities) measure the nanoparticle shape’s features such as the various circumscribed, midscribed, or inscribed diameters (or radii),
3. Find the closest match in all provided tables (if the particular size is not listed, use the equations provided in Tables 2, 4, and 5), focusing particularly on those matching most closely the specific shape(s) visible in your TEM images (if you see a specific polygonal faces, consider the values in the square brackets for the specific polygon),
4. Once this search is narrowed to the closest match, compare the *N*_*x*_/*N* values, with the lowest number giving the best match (*N*_*x*_ stands for: *N*_ve_, *N*_c_, *N*_*m*_, *N*_i_, or *N*_*e*_ or *N*_*v*_ which is the number of gold atoms calculated from the volume of a polyhedron with the radius of an edge-scribed sphere, from a volume of a sphere with circumscribed diameter, from a volume of a sphere with midscribed diameter, from a volume of a sphere with inscribed diameter, from the volume of an elliptically shaped particle, or the volume of a given regular polyhedron, respectively),
5. The closest match between shape and the lowest number of *N*_*x*_/*N* should give the nearest composition for the gold nanoparticle (nanocluster) core composition, and finally,
6. Use this number (*n*) for the Au_n_ particle (or numbers if multimodal size distribution is observed), compare to the quasi-spherical model (Eq. 1) to see discrepancy, and elucidate full composition including ligand shell (number of thiolates) using methods including, but not limited to NMR, elemental analysis and TGA. Consider arguments of ligand density as described in the previous section.

**Fig. 12:**
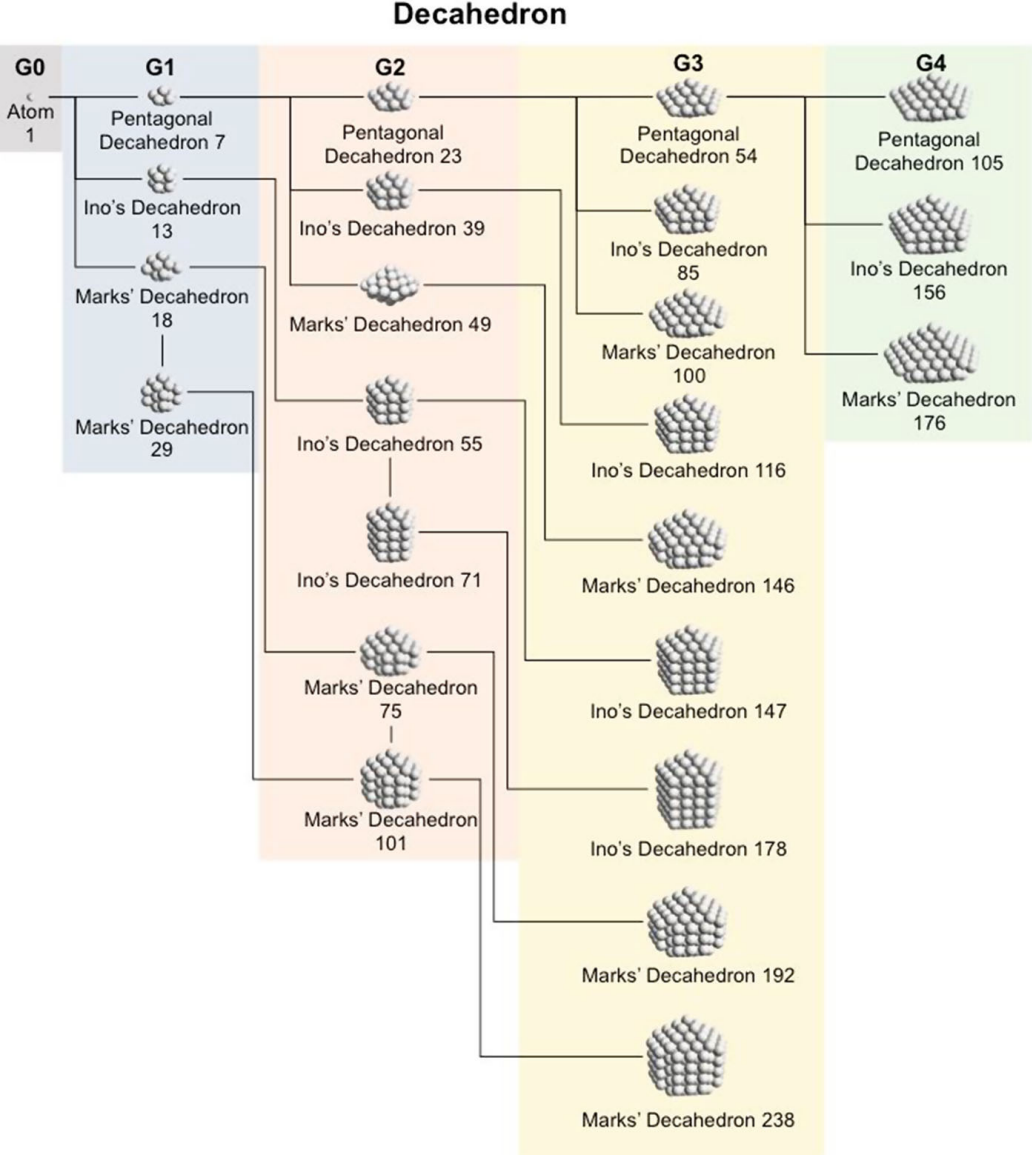
Generation tree for pentagonal decahedra as well as Ino’s and Marks’ decahedra

**Fig. 13:**
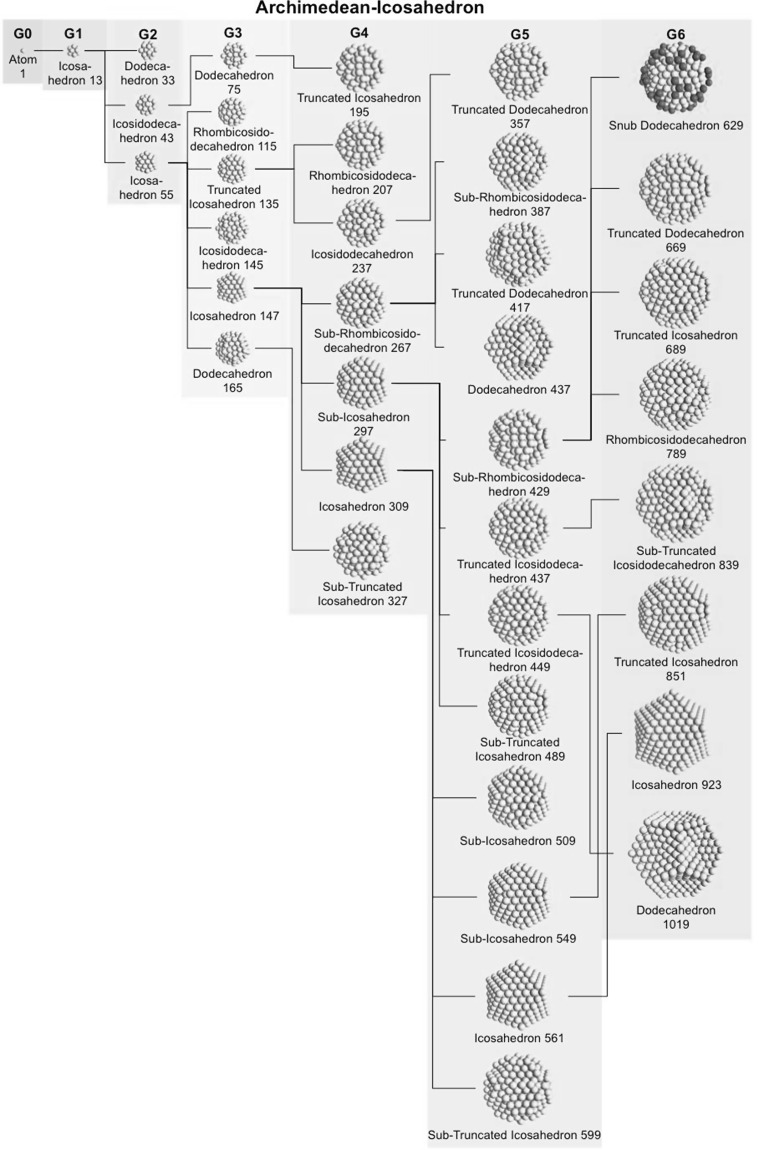
Generation tree for Archimedean icosahedra

**Fig. 14:**
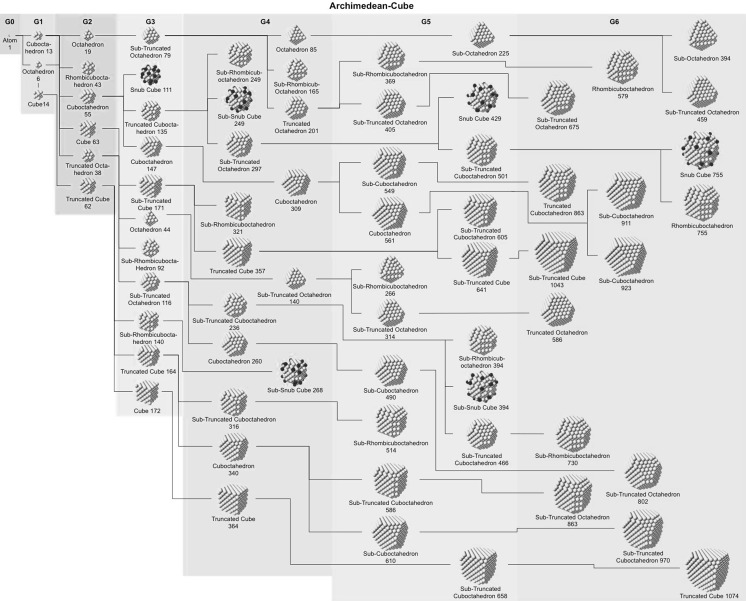
Generation tree for Archimedean cubes

In Figs. 12, 13, and 14, we also provide nanocluster generation trees for decahedra, Archimedean icosahedra, and Archimedean cubes, which should help understand connections between clusters and core structures as well as facilitate making the most suitable choices when analyzing TEM images.

## Discussion

As one can appreciate, the approach pursued here, culminating in the presented nanoparticle tables, equations, and models, solely relies on geometrical considerations not precise electronic and atomic structural information, which are only accessible for well-defined clusters whose structure was solved by X-ray diffraction (Chen et al. 2015; Jadzinsky et al. 2007; Zeng et al. 2014, 2015) or high-resolution single-particle TEM (Azubel et al. 2014) (aberration-corrected TEM, vide supra). Numerous groups have made significant and important progress in applying density functional theory and other numerical as well as computational approaches to determine gold nanoparticle sizes, structures, and energetics (Barnard 2010; Barnard and Chen 2011; Barnard and Curtiss 2006; Barnard et al. 2009; McKenna 2009; Negishi et al. 2015) and focus increasingly on the very challenging task of elucidating the structure of the thiolate ligand shell (Barnard 2013; Xu et al. 2015). A closer look at these modeling and simulation data on various gold nanoparticle sizes and shapes, however, reveals that the current geometrically derived data tables and models capture these and that the implementation of both approaches endows experimental scientists with a powerful tool for the elucidation of nanoparticle composition. In addition, the geometrical models can much faster survey a greater number of nanoparticles and nanoclusters (including nanoparticles with larger diameters and many more gold atoms in the core) much faster. Practically speaking, the presented equations and tables are easily adjustable (via the radius of the metal atom) for the determination of the composition of other metal nanoparticles with fcc lattices (Pd, Pt, Ni (Lin et al. 2011) as well as coinage metals Ag, Cu); perhaps even alloy-type metal nanoparticles if elemental composition is determined upfront. The predicted sequences of preferred shapes by Guisbiers et al. should here be tremendously helpful (Guisbiers et al. 2014). Of the 130 + clusters included in the current tables and models, several have not been experimentally observed for gold nanoparticles as of yet, and specific shapes observed for other transition metals are not included (e.g., tetrahedral for Pd nanoparticles (Barnard 2012)). Expansion to other shapes is a focus of future work, and numerical and theoretical methods recently presented by Barnard et al. will be used as guide for metal nanorods (Gonzalez et al. 2013). Finally, the section on shell structures is currently limited to the most frequently used thiolates and not considering other ligand motifs such as amines or phosphines among others. Thinking about the vast number of thiolate ligands reported in the literature, steric considerations are extremely difficult to include in any model system (Burgi 2015; Hakkinen 2012), especially since more and more sophisticated functions expected from gold nanoparticles require functional ligands with, for example, luminescent properties, binding capabilities to proteins, chirality, drug delivery, and many more.

## Conclusions

Centered specifically around geometrical considerations, this compendium of tables, models, and equations serves as an easy-to-use, straightforward guide for experimental scientists synthesizing thiol-protected gold nanoparticles in the laboratory to assist them in calculating the nanoparticle composition based on geometric information deduced from TEM imaging and image analysis. The majority of research thrusts and applications focusing on thiol-protected metal nanoparticles do not require the precision of well-defined metal clusters, although synthetic approaches to obtain such clusters are tirelessly pursued and refined. Nevertheless, predicting and analytically confirming the composition of all other metal nanoparticles as accurately as possible is critical for fundamental and applied research alike. A nanoparticle’s composition significantly affects its properties and defines its function, irrespective of its use in applications ranging from drug delivery and cancer diagnostics to metamaterials and chiral discriminators. With the anticipated transformation of these tables, models, and equations to a web-based tool (that would also permit viewing of model clusters from various perspectives), we trust that experimental scientists will be provided with an invaluable, helpful, and expandable tool for the elucidation of metal nanoparticle compositions.

## Electronic supplementary material

Below is the link to the electronic supplementary material.

Supplementary material 1 (PDF 28256 kb)

## Polyhedron

*L*: Edge length of polyhedron
*V*: Volume of polyhedron
*S*: Surface area of polyhedron

## Platonic and archimedean solids

*R*_c_: Circumscribed radius
*R*_m_: Midscribed radius
*R*_i_: Inscribed radius
*V*_*R*c_: Volume of sphere with circumscribed radius (*R*_c_)
*V*_*Rm*_: Volume of sphere with midscribed radius (*R*_m_)
*V*_*Ri*_: Volume of sphere with inscribed radius (*R*_*r*_)
*S*_*Rc*_: Surface area of sphere with circumscribed radius (*R*_c_)
*S*_*Rm*_: Surface area of sphere with midscribed radius (*R*_m_)
*S*_*Ri*_: Surface area of sphere with inscribed radius (*R*_*r*_)

## Catalan solids

*a*: Edge length of dual solid in Catalan solids
*R*_v_: Vertex radius
*R*_e_: Edge-scribed radius
*R*_i_: Inscribed radius
*V*_*Rv*_: Volume of sphere with vertex radius (*R*_v_)
*V*_*Re*_: Volume of sphere with edge-scribed radius (*R*_e_)
*V*_*Ri*_: Volume of sphere with inscribed radius (*R*_*r*_)
*S*_*Rv*_: Surface area of sphere with vertex radius (*R*_v_)
*S*_*Re*_: Surface area of sphere with edge-scribed radius (*R*_e_)
*S*_*Ri*_: Surface area of sphere with inscribed radius (*R*_*r*_)

## Parameter in cluster

*n*: Generation of gold nanoparticle
*D*: Diameter of gold nanoparticle
*L*: Edge length of polyhedron (cluster)
*L*_*ico*_: Edge length of icosahedron
*r*_Au_: Radius of gold atom = 1.44 Å
*R*_*cs*_: Circumscribed radius for an icosahedron
*R*_c_: Circumscribed radius
*R*_m_: Midscribed radius
*R*_i_: Inscribed radius
*D*_c_: Circumscribed diameter = 2*R*_c_
*D*_m_: Midscribed diameter = 2*R*_m_
*D*_i_: Inscribed diameter = 2*R*_i_
*V*_NP_: Volume of gold nanoparticle
*V*_Au_: Volume of gold atom = 17 Å^3^
*V*_ico_: Volume of icosahedron
*N*_cs_: Number of gold atoms in a circumscribed sphere for an icosahedron
*N*_ico_: Number of gold atoms in a regular icosahedron
*M*_*N*_: Magic number for gold cluster
*V*: Volume of polyhedron (cluster)
*a*: Edge length of dual solid in Catalan solids
*R*_v_: Vertex radius
*R*_e_: Edge-scribed radius
*N*_*Au*_: Number of gold atoms calculated assuming a quasi-spherical gold nanoparticle shape
*N*_ve_: Number of gold atoms calculated from volume of sphere with vertex radius (*N*_ve_) = *V*_*Rv*_/*V*_Au_
*N*_es_: Number of gold atoms calculated from volume of sphere with edge-scribed radius (*N*_es_) = *V*_*Re*_/*V*_Au_
*N*: Number of gold atoms in cluster
*N*_*v*_: Number of gold atoms calculated from volume of polyhedron = *V*/*V*_Au_ (*V*_Au_: volume of gold atom = 17 Å^3^)
*N*_c_: Number of gold atoms calculated from volume of sphere with circumscribed radius (*R*_c_) = *V*_*Rc*_/*V*_Au_
*N*_*m*_: Number of gold atoms calculated from volume of sphere with midscribed radius (*R*_m_) = *V*_*Rm*_/*V*_Au_
*N*_*i*_: Number of gold atoms calculated from volume of sphere with inscribed radius (*R*_i_) = *V*_*Ri*_/*V*_Au_
*S*_c_: Surface area of core
*N*_L_: Number of ligands on the core surface
*ρ*_*L*_: Ligand density

## Decahedron

*h*: Height of decahedron
*w*: Width of decahedron
*N*_*e*_: Number of gold atom calculated from volume of ellipsoid
*S*_Pe_: Surface area of pentagonal decahedron
*S*_Ino_: Surface area of Ino’s decahedron
*S*_Ma_: Surface area of Marks’ decahedron
