# Supplementary material for: Determining the composition of gold nanoparticles: a compilation of shapes, sizes, and calculations using geometric considerations
Source: J Nanopart Res. 2016 Oct 3;18(10):295. doi: 10.1007/s11051-016-3587-7 (PMC5047942; doi:10.1007/s11051-016-3587-7)
Supplement: Supplementary file 1 — Supplementary material 1 (PDF 28256 kb) [file 11051_2016_3587_MOESM1_ESM.pdf]

# A Practical Guide to Calculate the Number of Atoms and Overall Composition of Metal Nanoparticles using Geometric Considerations

Taizo Mori*[#] and Torsten Hegmann*

Chemical Physics Interdisciplinary Program, Liquid Crystal Institute, Kent State University, Kent, OH 44242-0001 U.S.A.

## Electronic Supplementary Material

| Section | Page |
|---|---|
| Section S1 – Equations and calculations for Platonic, Archimedean and Catalan solids | 2 |
| Section S2 – Data for polyhedra | 14 |
| Section S3 – Data for Archimedean Icosahedra | 27 |
| Section S4 – Data for Archimedean Cubes | 32 |
| Section S5 – Example calculations | 41 |
| Section S6 – *xyz* Coordinates | 46 |
| References | 46 |

## Section S1 – Equations and calculations for Platonic, Archimedean and Catalan solids[S1]

### Platonic Solids

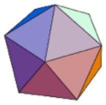

**Icosahedron**
Vertices: 12
Faces: 20 [3]
Edges: 30
Dual solid: Dodecahedron

$V = \frac{5(3+\sqrt{5})}{12}L^3 = 2.182L^3$ $\quad S = 5\sqrt{3}L^2 = 8.660L^2$

$R_c = \frac{\sqrt{10+2\sqrt{5}}}{4}L = 0.9511L$ $\quad R_m = \frac{1+\sqrt{5}}{4}L = 0.8090L$ $\quad R_i = \frac{3\sqrt{3}+\sqrt{15}}{12}L = 0.7558L$

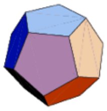

**Dodecahedron**
Vertices: 20
Faces: 12 [5]
Edges: 30
Dual solid: Icosahedron

$V = \frac{15+7\sqrt{5}}{4}L^3 = 7.663L^3$ $\quad S = 3\sqrt{25+10\sqrt{5}}L^2 = 20.65L^2$

$R_c = \frac{\sqrt{3}+\sqrt{15}}{4}L = 1.4013L$ $\quad R_m = \frac{3+\sqrt{5}}{4}L = 1.3090L$ $\quad R_i = \frac{\sqrt{10(25+11\sqrt{5})}}{20}L = 1.1135L$

### Archimedean Solids

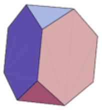

**Truncated Tetrahedron**
Vertices: 12
Faces: 8 = 4[3] + 4[6]
Edges: 18
Dual solid: Triakis Tetrahedron

$V = \frac{23}{12}\sqrt{2}L^3 = 2.711L^3$ $\quad S = 7\sqrt{3}L^2 = 12.12L^2$

$R_c = \frac{\sqrt{22}}{4}L = 1.1726L$ $\quad R_m = \frac{3\sqrt{2}}{4}L = 1.0606L$ $\quad$ [3]: $R_i = \frac{5\sqrt{6}}{12}L = 1.0206L$

[6]: $R_i = \frac{\sqrt{6}}{4}L = 0.6123L$

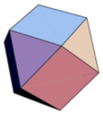

**Cuboctahedron**
Vertices: 12
Faces: 14 = 8[3] + 6[4]
Edges: 24
Dual solid: Rhombic Dodecahedron

$V = \frac{5}{3}\sqrt{2}L^3 = 2.357L^3$ $\quad S = (6+2\sqrt{3})L^2 = 9.464L^2$

$R_c = L$ $\quad R_m = \frac{\sqrt{3}}{2}L = 0.8660L$ $\quad$ [3]: $R_i = \frac{\sqrt{6}}{3}L = 0.8165L$

[4]: $R_i = \frac{\sqrt{2}}{2}L = 0.7071L$

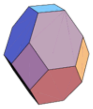

**Truncated Octahedron**
Vertices: 24
Faces: 14 = 6[4] + 8[6]
Edges: 36
Dual solid: Tetrakis Hexahedron

$V = 8\sqrt{2}L^3 = 11.31L^3$ $\quad S = 6(1+2\sqrt{3})L^2 = 26.78L^2$

$R_c = \frac{\sqrt{10}}{2}L = 1.5811L$ $\quad R_m = 1.5L$ $\quad$ [4]: $R_i = \sqrt{2}L = 1.4142L$

[6]: $R_i = \frac{\sqrt{6}}{2}L = 1.2247L$

*$V$: Volume, $S$: Surface area, $L$: Edge length, $R_c$: Circumscribed radius, $R_m$: Midscribed radius, $R_i$: Inscribed radius*

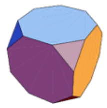

**Truncated Cube**
Vertices: 24
Faces: 14 = 8[3] + 6[8]
Edges: 36
Dual solid: Triakis Octahedron

$$V = 7\left(1 + \frac{2\sqrt{2}}{3}\right)L^3 = 13.56L^3 \qquad S = 2\left(6 + 6\sqrt{2} + \sqrt{3}\right)L^2 = 32.43L^2$$

$$R_c = \frac{\sqrt{7 + 4\sqrt{2}}}{2}L = 1.7789L \qquad R_m = \left(1 + \frac{\sqrt{2}}{2}\right)L = 1.7071L$$

$$[3]:\ R_i = \frac{3\sqrt{3} + 2\sqrt{6}}{6}L = 1.6825L$$

$$[8]:\ R_i = \frac{1 + \sqrt{2}}{2}L = 1.2071L$$

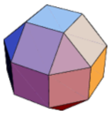

**Rhombicuboctahedron**
Vertices: 24
Faces: 26 = 8[3] + 18[4]
Edges: 48
Dual solid: Deltoidal Icositetrahedron

$$V = \frac{2}{3}\left(6 + 5\sqrt{2}\right)L^3 = 8.7140L^3 \qquad S = \left(18 + 2\sqrt{3}\right)L^2 = 21.46L^2$$

$$R_c = \frac{\sqrt{5 + 2\sqrt{2}}}{2}L = 1.3990L \qquad R_m = \frac{\sqrt{2\left(2 + \sqrt{2}\right)}}{2}L = 1.3066L$$

$$[3]:\ R_i = \frac{3\sqrt{3} + \sqrt{6}}{6}L = 1.2743L$$

$$[4]:\ R_i = \frac{1 + \sqrt{2}}{2}L = 1.2071L$$

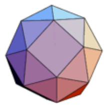

**Snub Cube (enantiomer)**
Vertices: 24
Faces: 38 = 32[3] + 6[4]
Edges: 60
Dual solid: Pentagonal Icositetrahedron

$$V = \frac{3\sqrt{\tau - 1} + 4\sqrt{\tau + 1}}{3\sqrt{2 - \tau}}L^3 = 7.8895L^3 \qquad S = \left(6 + 8\sqrt{3}\right)L^2 = 18.86L^2$$

$$R_c = \frac{\sqrt{d^2 + L^2}}{2} = 1.3437L \qquad R_m = \frac{1}{\sqrt{4(2 - \tau)}}L = 1.2472L$$

$$[3]:\ R_i = \frac{\sqrt{d^2 - L^2}}{2} = 1.2134L$$

$$\tau = (X^3 - X^2 - X - 1)_1 = \frac{1}{3}\left(1 + \sqrt[3]{19 - 2\sqrt{33}} + \sqrt[3]{19 + 2\sqrt{33}}\right) = 1.83929$$

$$d = \frac{L}{\sqrt{2 - \tau}}$$

$$[4]:\ R_i = \frac{1}{2}\sqrt{d^2 - \frac{L^2}{3}} = 1.1426L$$

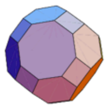

**Truncated Cuboctahedron**
Vertices: 48 (48[3])
Faces: 26 = 12[4] + 8[6] +6[8]
Edges: 72
Dual solid: Disdyakis Dodecahedron

$$V = \left(22 + 14\sqrt{2}\right)L^3 = 41.79L^3 \qquad S = 12\left(2 + \sqrt{2} + \sqrt{3}\right)L^2 = 61.76L^2$$

$$R_c = \frac{\sqrt{13 + 6\sqrt{2}}}{2}L = 2.3176L \qquad R_m = \frac{\sqrt{12 + 6\sqrt{2}}}{2}L = 2.2630L$$

$$[4]:\ R_i = \frac{3 + \sqrt{2}}{2} = 2.2071L$$

$$[6]:\ R_i = \frac{\sqrt{3} + \sqrt{6}}{2} = 2.0908L$$

$$[8]:\ R_i = \frac{1 + 2\sqrt{2}}{2} = 1.9142L$$

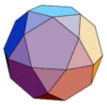

**Icosidodecahedron**
Vertices: 30
Faces: 32 = 20[3] + 12[5]
Edges: 60
Dual solid: Rhombic Triacontahedron

$$V = \frac{45 + 17\sqrt{5}}{6}L^3 = 13.84L^3 \qquad S = \left(5\sqrt{3} + 3\sqrt{5}\sqrt{5 + 2\sqrt{5}}\right)L^2 = 29.31L^2$$

$$R_c = \frac{1 + \sqrt{5}}{2}L = 1.6180L \qquad R_m = \frac{\sqrt{5 + 2\sqrt{5}}}{2}L = 1.5388L$$

$$[3]\text{: } R_{in} = \sqrt{\frac{7 + 3\sqrt{5}}{6}} = 1.5115L$$

$$[5]\text{: } R_{in} = \sqrt{\frac{5 + 2\sqrt{5}}{5}}L = 1.3764L$$

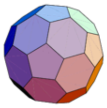

**Truncated Icosahedron**
Vertices: 60
Faces: 32 = 12[5] + 20[6]
Edges: 90
Dual solid: Pentakis Dodecahedron

$$V = \frac{125 + 43\sqrt{5}}{4}L^3 = 55.29L^3 \qquad S = 3\left(10\sqrt{3} + \sqrt{5}\sqrt{5 + 2\sqrt{5}}\right)L^2 = 72.61L^2$$

$$R_c = \frac{\sqrt{58 + 18\sqrt{5}}}{4}L = 2.4780L \qquad R_m = \frac{3(1 + \sqrt{5})}{4}L = 2.4271L$$

$$[5]\text{: } R_i = \frac{\sqrt{10(125 + 41\sqrt{5})}}{20} = 2.3274L$$

$$[6]\text{: } R_i = \frac{3\sqrt{5} + \sqrt{15}}{4} = 2.2672L$$

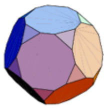

**Truncated Dodecahedron**
Vertices: 60
Faces: 32 = 20[3] + 12[10]
Edges: 90
Dual solid: Triakis Icosahedron

$$V = \frac{5}{12}(99 + 47\sqrt{5})L^3 = 85.04L^3 \qquad S = 5\left(\sqrt{3} + 6\sqrt{5 + 2\sqrt{5}}\right)L^2 = 100.99L^2$$

$$R_c = \frac{\sqrt{2(37 + 15\sqrt{5})}}{4}L = 2.9695L \qquad R_m = \frac{5 + 3\sqrt{5}}{4}L = 2.9271L$$

$$[3]\text{: } R_i = \frac{9\sqrt{3} + 5\sqrt{15}}{12} = 2.9128L$$

$$[10]\text{: } R_i = \frac{\sqrt{2(25 + 11\sqrt{5})}}{4}L = 2.499L$$

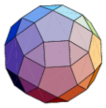

**Rhombicosidodecahedron**
Vertices: 60
Faces: 62 = 20[3] + 30[4] + 12[5]
Edges: 120
Dual solid: Deltoidal Hexecontahedron

$$V = \left(20 + \frac{29}{3}\sqrt{5}\right)L^3 = 41.62L^3 \qquad S = \left\{30 + \sqrt{30\left[10 + 3\sqrt{5} + \sqrt{15(5 + 2\sqrt{5})}\right]}\right\}L^2 = 59.31L^2$$

$$R_c = \frac{\sqrt{11 + 4\sqrt{5}}}{2}L = 2.2330L \qquad R_m = \frac{\sqrt{2(5 + 2\sqrt{5})}}{2}L = 2.1763L$$

$$[3]\text{: } R_i = \frac{3\sqrt{3} + 2\sqrt{15}}{6} = 2.1571L$$

$$[4]\text{: } R_i = \frac{2 + \sqrt{5}}{2} = 2.1180L$$

$$[5]\text{: } R_i = \frac{3\sqrt{5(5 + 2\sqrt{5})}}{10} = 2.0653L$$

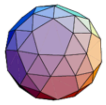

**Snub Dodecahedron (enantiomer)**
Vertices: 60
Faces: 92 = 80[3] + 12[5]
Edges: 150
Dual solid: Pentagonal Hexecontahedron

$$\phi = \frac{1 + \sqrt{5}}{2} \qquad \xi = \sqrt[3]{\frac{\phi + \sqrt{\phi - {}^{5}/_{27}}}{2}} + \sqrt[3]{\frac{\phi - \sqrt{\phi - {}^{5}/_{27}}}{2}}$$

$$V = \frac{12\xi^2(3\phi + 1) - \xi(36\phi + 7) - (53\phi + 6)}{6\sqrt{6 - \xi^2}^3} = 37.62L^3 \qquad S = 20\sqrt{3} + 3\sqrt{25 + 10\sqrt{5}} = 55.289$$

$$R_c = \phi\sqrt{\xi(\xi + \phi)} + \frac{3 - \phi}{2} = 2.1558L$$

$$R_m = \frac{\phi\sqrt{\xi(\xi + \phi) + 1}}{2}L = 2.0971L$$

$$[3]\text{: } R_i = \frac{\phi\xi\sqrt{3[\xi(\xi + \phi) + 1]}}{6} = 2.0771L$$

$$[5]\text{: } R_i = \sqrt{20\left[5\left(\xi + \frac{1}{\xi}\right)(2\phi + 1)\right] + \frac{11\phi + 12}{20}} = 1.9809L$$

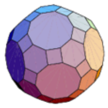

**Truncated Icosidodecahedron**
Vertices: 120
Faces: 62 = 30[4] + 20[6] + 12[12]]
Edges: 180
Dual solid: Disdyakis Triacontahedron

$$V = (95 + 50\sqrt{5})L^3 = 206.80L^3 \qquad S = 30\left(1 + \sqrt{2\left(4 + \sqrt{5} + \sqrt{15 + 6\sqrt{6}}\right)}\right)L^2 = 175.03L^2$$

$$R_c = \frac{\sqrt{31 + 12\sqrt{5}}}{2}L = 3.8024L$$

$$R_m = \frac{\sqrt{6(5 + 2\sqrt{2})}}{2}L = 3.7694L$$

$$[4]\text{: } R_i = \frac{3 + 2\sqrt{5}}{2} = 3.7361L$$

$$[6]\text{: } R_i = \frac{2\sqrt{3} + \sqrt{15}}{2} = 3.6685L$$

$$[12]\text{: } R_i = \frac{\sqrt{5(5 + 2\sqrt{5})}}{2} = 3.4410L$$

## Catalan Solids

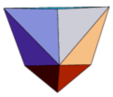

**Triakis Tetrahedron**

]ertices: 8
Faces: 12 (isosceles triangles)
Edges: 18 = $12L_s + 6L_l$
Dual solid: Truncated Tetrahedron

$V = \frac{8\sqrt{2}}{20}a^3 = 5.7276a^3$

[3]: $R_v = \frac{9\sqrt{6}}{20}a = 1.1023a$

[6]: $R_v = \frac{3\sqrt{6}}{4}a = 1.8371a$

$S = \frac{27}{5}\sqrt{11}a^2 = 17.91$

$R_e = \frac{3\sqrt{2}}{3}a = 1.0607a$

$L_s = \frac{9}{5}a = 1.8a$
$L_l = 3a$

$R_i = \frac{9\sqrt{22}}{44}a = 0.9594a$

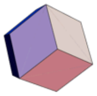

**Rhombic Dodecahedron**

Vertices: 14
Faces: 12 (rhombi)
Edges: 24
Dual solid: Cuboctahedron

$V = \frac{27\sqrt{2}}{16}a^3 = 2.3865a^3$

[3]: $R_v = \frac{3\sqrt{6}}{8}a = 0.9186a$

[4]: $R_v = \frac{3\sqrt{2}}{4}a = 1.0607a$

$S = \frac{4\sqrt{2}}{27}a^2 = 9.5460a^2$

$R_e = \frac{2\sqrt{2}}{3}a = 0.9428a$

$L = \frac{3\sqrt{6}}{8}a = 0.9186a$

$R_i = \frac{3}{4}a = 0.75a$

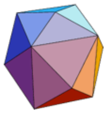

**Tetrakis Hexahedron**

Vertices: 14
Faces: 24 (isosceles triangles)
Edges: 36 = $24L_s + 12L_l$
Dual solid: Truncated Octahedron

$V = \frac{81\sqrt{2}}{8}a^3 = 14.32a^3$

[4]: $R_v = \frac{9\sqrt{2}}{8}a = 1.5910a$

[6]: $R_v = \frac{3\sqrt{6}}{4}a = 1.8371a$

$S = \frac{27\sqrt{5}}{2}a^2 = 30.19a^2$

$R_e = \frac{3}{2}a = 1.5a$

$L_s = \frac{9\sqrt{2}}{8}a = 1.5910a$
$L_l = \frac{3\sqrt{2}}{2}a = 2.1213a$

$R_i = \frac{9\sqrt{10}}{20}a = 1.423a$

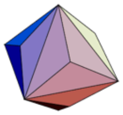

**Triakis Octahedron**

Vertices: 14
Faces: 24 (isosceles triangles)
Edges: 36 = $24L_s + 12L_l$
Dual solid: Truncated Cube

$V = 4(3+\sqrt{2})a^3 = 23.31a^3$

[3]: $R_v = \sqrt{3}a = 1.7321a$
[8]: $R_v = (1+\sqrt{2})a = 2.4142a$

$S = 12\sqrt{7+4\sqrt{2}}a^2 = 42.69a^2$

$R_e = 1 + \frac{\sqrt{2}}{2}a = 1.7072a$

$L_s = 2a$
$L_l = (2+\sqrt{2})a = 3.4142a$

$R_i = \frac{\sqrt{17(23+16\sqrt{2})}}{17}a = 1.6383a$

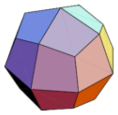

**Deltoidal Icositetrahedron**

Vertices: 26
Faces: 24 (tri-equiangular kites)
Edges: 48 = $24L_s + 24L_l$
Dual solid: Rhombicuboctahedron

$V = \sqrt{122+71\sqrt{2}}L_s^3 = \frac{16(1+2\sqrt{2})}{7}a^3$
$= 8.7507a^3$

$S = 6\sqrt{29-2\sqrt{2}}L_s^2 = 21.51a^2$

$L_s = \frac{2\sqrt{10-\sqrt{2}}}{7}a = 0.8372a$
$L_l = \sqrt{2(2-\sqrt{2})}a = 1.0824a$

[3]: $R_v = \frac{4\sqrt{3}+\sqrt{6}}{7}a = 1.3397a$
[4]: $R_v = \sqrt{2}a = 1.4142a$

$R_e = \frac{\sqrt{2(2+\sqrt{2})}}{2}a = 1.3066a$

$R_i = \frac{\sqrt{34(7+4\sqrt{2})}}{17}a = 1.2203a$

$a$: *Edge length of dual solid*, $R_v$: *Vertex radius*, $R_e$: *Edge − scribed radius*

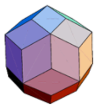

**Rhombic Triacontahedron**

Vertices: 32
Faces: 30 (rhombi)
Edges: 60
Dual solid: Icosidodecahedron

$$V = 4\sqrt{5+2\sqrt{5}}L^3 = 12.31L^3 = \frac{25\sqrt{5+2\sqrt{5}}}{16}a^3 = 14.80a^3$$

$$S = 12\sqrt{5}L^2 = 26.84L^2 = 30.34a^2$$

$$L = \frac{\sqrt{10(5+\sqrt{5})}}{8}a = 1.0633\text{a}$$

$$[3]\colon\ R_{cs} = \frac{5\sqrt{3}+\sqrt{15}}{8}a = 1.5667a$$

$$[5]\colon\ R_{cs} = \frac{\sqrt{5(5+2\sqrt{5})}}{4}a = 1.7204a$$

$$R_e = \frac{\sqrt{5+2\sqrt{5}}}{2}a = 1.5388a$$

$$R_i = \frac{5+3\sqrt{5}}{8}a = 1.4635a$$

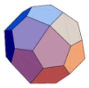

**Pentagonal Icositetrahedron (enantiomer)**

Vertices: 38
Faces: 24 (mirror-symmetric pentagons)
Edges: 60 $= 36L_s + 24L_l$
Dual solid: Snub Cube

$$V = \sqrt{\frac{11(\tau-4)}{2(20\tau-37)}}a^3 = 7.4474a^3$$

$$S = 3\sqrt{\frac{22(5\tau-1)}{4\tau-3}}a^2 = 19.2999a^2$$

$$L_s = \frac{1}{\sqrt{\tau+1}}a = 0.593465a$$

$$L_l = \frac{\sqrt{\tau+1}}{2}a = 1.0824a$$

$$[3]\colon\ R_v = \frac{\sqrt{2\left[6+\sqrt[3]{6(9+\sqrt{33})}+\sqrt[3]{6(9-\sqrt{33})}\right]}}{4}a = 1.2820L$$

$$[4]\colon\ R_v = \frac{\sqrt{6\left[14+\sqrt[3]{2(1777+33\sqrt{33})}+\sqrt[3]{2(1777-33\sqrt{33})}\right]}}{12}a = 1.3614a$$

$$R_e = \frac{\sqrt{3\left[7+\sqrt[3]{199+3\sqrt{33}}+\sqrt[3]{199-3\sqrt{33}}\right]}}{6}a = 1.2472a$$

$$R_i = \frac{\sqrt{42\left[78+\sqrt[3]{66(6039+49\sqrt{33})}+\sqrt[3]{66(6039-49\sqrt{33})}\right]}}{84}a = 1.1577a$$

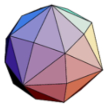

**Disdyakis Dodecahedron**

Vertices: 26
Faces: 48 (acute triangles)
Edges: 72 $= 24L_s + 24L_{lm} + 24L_l$
Dual solid: Truncated Cuboctahedron

$$V = \frac{144(1+\sqrt{2})}{7}a^3 = 49.6638a^3$$

$$S = \frac{6\sqrt{783+436\sqrt{2}}}{7}L_s^2 = 67.42a^2$$

$$L_s = \frac{2\sqrt{3(10-\sqrt{2})}}{7}a = 1.4500a$$

$$L_m = \frac{3\sqrt{6(2+\sqrt{2})}}{7}a = 1.9397a$$

$$L_l = \frac{2\sqrt{6(10+\sqrt{2})}}{7}a = 2.3644a$$

$$[4]\colon\ R_v = \frac{3(4+\sqrt{7})}{7}a = 2.3204a$$

$$[6]\colon\ R_v = \sqrt{6}a = 2.4495a$$

$$[8]\colon\ R_v = \frac{3(2+3\sqrt{2})}{7}a = 2.6754a$$

$$R_e = \frac{\sqrt{6(2+\sqrt{3})}}{2}a = 2.2630a$$

$$R_i = \frac{\sqrt{1746(15+8\sqrt{2})}}{97}a = 2.2097a$$

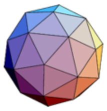

**Pentakis Dodecahedron**
Vertices: 32
Faces: 60 (isosceles triangles)
Edges: 90 = $60L_s + 30L_l$
Dual solid: Truncated Icosahedron

$$V = \frac{405(9+\sqrt{5})}{76}a^3 = \frac{5(41+25\sqrt{5})}{36}L_s^3 = 59.88a^3$$

$$S = \frac{5}{3}\sqrt{\frac{421+63\sqrt{5}}{2}}L_s^2 = 75.56a^2$$

$$L_s = \frac{9(2\sqrt{5}-1)}{19}a = 1.6447a$$

$$L_l = \frac{3(\sqrt{5}-1)}{2}a = 1.8541a$$

$$[5]:\ R_v = \frac{9\sqrt{65+22\sqrt{5}}}{38}a = 2.5309a$$

$$[6]:\ R_v = \frac{3\sqrt{3}}{2}a = 2.5981a$$

$$R_e = \frac{3\sqrt{1+\sqrt{5}}}{4}a = 2.4271a$$

$$R_i = \frac{9\sqrt{109(17+6\sqrt{5})}}{218}a = 2.3771a$$

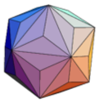

**Triakis Icosahedron**
Vertices:
Faces: 60 (isosceles triangles)
Edges: 90 = $60L_s + 30L_l$
Dual solid: Truncated Dodecahedron

$$V = \frac{125(19+9\sqrt{5})}{44}a^3 = 111.15a^3$$

$$S = \frac{75}{11}\sqrt{\frac{313+117\sqrt{5}}{2}}a^2 = 115.57a^2$$

$$L_s = \frac{5(7+\sqrt{5})}{22}a = 2.0991a$$

$$L_l = \frac{5+\sqrt{5}}{2}a = 3.6180a$$

$$[3]:\ R_v = \frac{5(3\sqrt{3}+2\sqrt{15})}{22}a = 2.9414a$$

$$[10]:\ R_v = \frac{\sqrt{5(5+2\sqrt{5})}}{2}a = 3.4410a$$

$$R_e = \frac{5+3\sqrt{5}}{4}a = 2.9271a$$

$$R_i = \frac{5\sqrt{61(41+18\sqrt{5})}}{122}a = 2.8853a$$

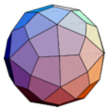

**Deltoidal Hexecontahedron**
Vertices: 62
Faces: 60 (kites)
Edges: 120 = $60L_s + 60L_l$
Dual solid: Rhombicosidodecahedron

$$V = \frac{100(5+4\sqrt{5})}{33}a^3 = 42.26a^3$$

$$S = \frac{100}{11}\sqrt{79-16\sqrt{5}}a^2 = 59.77a^2$$

$$L_s = \frac{\sqrt{5(85-31\sqrt{5})}}{11}a = 0.8050a$$

$$L_l = \frac{\sqrt{5(5-\sqrt{5})}}{3}a = 1.2392a$$

$$[3]:\ R_v = \frac{5\sqrt{3}+4\sqrt{15}}{11}a = 2.1957a$$

$$[4]:\ R_v = \sqrt{5}a = 2.2361a$$

$$[5]:\ R_v = \frac{\sqrt{5(5+2\sqrt{5})}}{3}a = 2.2940a$$

$$R_e = \frac{\sqrt{2(5+2\sqrt{5})}}{2}a = 2.1763a$$

$$R_i = \frac{\sqrt{205(19+8\sqrt{5})}}{41}a = 2.1210a$$

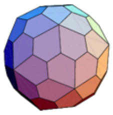

**Pentagonal Hexecontahedron (enantiomer)**

Vertices: 92 (80[3] + 12[5])
Faces: 60 (mirror-symmetric pentagons)
Edges: 150 = 90$L_s$ + 60$L_l$
Dual solid: Snub Dodecahedron

$$L_s = \frac{1}{\xi}a = 0.5829a$$

$$L_l = \frac{\xi(7\phi + 2) + (5\phi - 3) + \frac{2}{\xi}(8 - 3\phi)}{31}a = 1.0200a$$

$$R_e = \frac{\phi\sqrt{\xi(\xi + \phi) + 1}}{2}a = 2.0971a$$

$$\phi = \frac{1 + \sqrt{5}}{2} \qquad \xi = \sqrt[3]{\frac{\phi + \sqrt{\phi - 5/27}}{2}} + \sqrt[3]{\frac{\phi - \sqrt{\phi - 5/27}}{2}}$$

$$V = \frac{5\sqrt{\xi[\xi^2(11405\phi + 287) + \xi(14528\phi + 8265) + (2363\phi + 131416)]}}{62}a^3 = 37.59a^3$$

$$S = 55.28a^2$$

$$[3]:\ R_v = \frac{\sqrt{3\left(\xi\phi + \phi + 1 + \frac{1}{\xi}\right)}}{2}a = 2.1172a$$

$$[5]:\ R_v = \frac{\sqrt{\xi^2[(1067\phi + 1009) + \xi(2259\phi + 1168) + (941\phi + 1097)]}}{62}a = 2.2000a$$

$$R_i = \frac{\xi\sqrt{209[\xi^2(104\phi - 7) + \xi(153\phi + 52) + (195 - \phi)]}}{418}a = 2.0399a$$

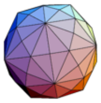

**Disdyakis Triacontahedron**

Vertices: 62 (30[4] + 20[6] + 12[10])
Faces: 120 (acute triangles)
Edges: 180 = 60$L_s$ + 60$L_{lm}$ + 60$L_l$
Dual solid: Truncated Icosidodecahedron

$$V = \frac{180(5 + 4\sqrt{5})}{11}a^3 = 228.18a^3 \qquad S = \frac{180}{11}\sqrt{179 - 24\sqrt{5}}a^2 = 183.20a^2$$

$$L_s = \frac{\sqrt{15(85 - 31\sqrt{5})}}{11}a = 1.3943a$$

$$L_m = \frac{3\sqrt{15(65 + 19\sqrt{5})}}{55}a = 2.1902a$$

$$L_l = \frac{2\sqrt{15(5 - \sqrt{5})}}{5}a = 2.5755a$$

$$[4]:\ R_v = \frac{3(5 + 4\sqrt{5})}{11}a = 3.8030a$$

$$[6]:\ R_v = \sqrt{15}a = 3.8730a$$

$$[10]:\ R_v = \frac{3\sqrt{5(5 + 2\sqrt{5})}}{5}a = 4.1291a$$

$$R_e = \frac{\sqrt{6(5 + 2\sqrt{5})}}{2}a = 3.7694a \qquad R_i = \frac{\sqrt{10845(39 + 16\sqrt{5})}}{241}a = 3.7366a$$

**Table S1.** Overview and summary of parameters from equations for polyhedra in Section 1 above.[S1]

| **Polyhedron** | $V$ | $S$ | $R_c$ | $V_{Rc}/V$ | $S_{Rc}/S$ | $R_m$ | $V_{Rm}/V$ | $S_{Rm}/S$ | $R_i$ | $V_{Ri}/V$ | $S_{Ri}/S$ |
|---|---|---|---|---|---|---|---|---|---|---|---|
| **⇣ Platonic and Archimedean Solids ⇣** | | | | | | | | | | | |
| 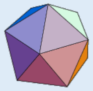 Icosahedron | 2.182 | 8.66 | 0.9511 | 1.65 | 1.31 | 0.8090 | 1.02 | 0.95 | 0.7558 | 0.83 | 0.83 |
| 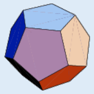 Dodecahedron | 7.663 | 20.65 | 1.4010 | 1.50 | 1.19 | 1.3090 | 1.23 | 1.04 | 1.1140 | 0.76 | 0.76 |
| 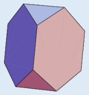 Truncated Tetrahedron | 2.711 | 12.12 | 1.1726 | 2.49 | 1.43 | 1.0606 | 1.84 | 1.17 | [3] 1.0206 | 1.64 | 1.08 |
| | | | | | | | | | [6] 0.6123 | 0.35 | 0.39 |
| 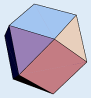 Cuboctahedron | 2.357 | 9.464 | 1.0000 | 1.78 | 1.33 | 0.8660 | 1.15 | 1.00 | [3] 0.8165 | 0.97 | 0.89 |
| | | | | | | | | | [4] 0.7071 | 0.63 | 0.66 |
| 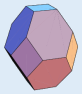 Truncated Octahedron | 11.31 | 26.78 | 1.5811 | 1.46 | 1.17 | 1.5000 | 1.25 | 1.06 | [4] 1.4142 | 1.05 | 0.94 |
| | | | | | | | | | [6] 1.2247 | 0.68 | 0.70 |

| | | | | | | | | | | | |
|---|---|---|---|---|---|---|---|---|---|---|---|
| 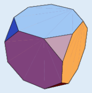 Truncated Cube | 13.56 | 32.43 | 1.7789 | 1.74 | 1.23 | 1.7071 | 1.54 | 1.13 | [3] 1.6825 | 1.47 | 1.10 |
| | | | | | | | | | [8] 1.2071 | 0.54 | 0.56 |
| 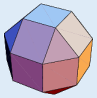 Rhombicubocta-hedron | 8.714 | 21.46 | 1.3990 | 1.32 | 1.15 | 1.3066 | 1.07 | 1.00 | [3] 1.2743 | 0.99 | 0.95 |
| | | | | | | | | | [4] 1.2071 | 0.85 | 0.85 |
| 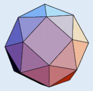 Snub Cube | 7.890 | 18.86 | 1.3437 | 1.29 | 1.20 | 1.2472 | 1.03 | 1.04 | [3] 1.2134 | 0.95 | 0.98 |
| | | | | | | | | | [4] 1.1426 | 0.79 | 0.87 |
| 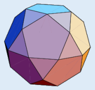 Icosidodecahedron | 13.84 | 29.31 | 1.6180 | 1.28 | 1.12 | 1.5388 | 1.10 | 1.02 | [3] 1.5115 | 1.05 | 0.98 |
| | | | | | | | | | [5] 1.3764 | 0.79 | 0.81 |
| 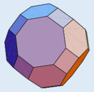 Truncated Cuboctahedron | 41.79 | 61.76 | 2.3176 | 1.25 | 1.09 | 2.2630 | 1.16 | 1.04 | [4] 2.2071 | 1.08 | 0.99 |
| | | | | | | | | | [6] 2.0908 | 0.92 | 0.89 |
| | | | | | | | | | [8] 1.9142 | 0.70 | 0.75 |

| | | | | | | | | | | |
|---|---|---|---|---|---|---|---|---|---|---|
| 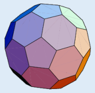 Truncated Icosahedron | 55.29 | 72.61 | 2.4780 | 1.15 | 1.06 | 2.4271 | 1.08 | 1.02 | [5] 2.3274 | 0.96 | 0.94 |
| | | | | | | | | | [6] 2.2672 | 0.88 | 0.89 |
| 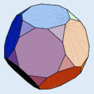 Truncated Dodecahedron | 85.04 | 100.99 | 2.9695 | 1.29 | 1.10 | 2.9271 | 1.24 | 1.07 | [3] 2.9128 | 1.22 | 1.06 |
| | | | | | | | | | [10] 2.4990 | 0.77 | 0.78 |
| 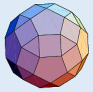 Rhombicosido-decahedron | 41.62 | 59.31 | 2.2330 | 1.12 | 1.06 | 2.1763 | 1.04 | 1.00 | [3] 2.1571 | 1.01 | 0.99 |
| | | | | | | | | | [4] 2.1180 | 0.96 | 0.95 |
| | | | | | | | | | [5] 2.0653 | 0.89 | 0.90 |
| 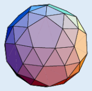 Snub Dodecahedron | 37.62 | 55.289 | 2.1558 | 1.12 | 1.06 | 2.0971 | 1.03 | 1.00 | [3] 2.0771 | 1.00 | 0.98 |
| | | | | | | | | | [5] 1.9809 | 0.87 | 0.89 |
| 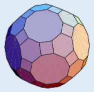 Truncated Icosidodecahedron | 206.8 | 175.03 | 3.8024 | 1.11 | 1.04 | 3.7694 | 1.08 | 1.02 | [4] 3.7361 | 1.06 | 1.00 |
| | | | | | | | | | [6] 3.6685 | 1.00 | 0.97 |

**⇣ Catalan Solids ⇣**

| | | | | | | | | | | | | |
|---|---|---|---|---|---|---|---|---|---|---|---|---|
| 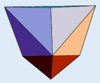 | Triakis Tetrahedron | 5.7276 | 17.91 | [3] 1.1023 | 0.98 | 0.85 | 1.0607 | 0.87 | 0.79 | 0.9594 | 0.65 | 0.65 |
| | | | | [6] 1.8371 | 4.53 | 2.37 | | | | | | |
| 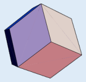 | Rhombic Dodecahedron | 2.3865 | 9.546 | [3] 0.9186 | 1.36 | 1.11 | 0.9428 | 1.47 | 1.17 | 0.7500 | 0.74 | 0.74 |
| | | | | [4] 1.0607 | 2.09 | 1.48 | | | | | | |
| 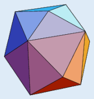 | Tetrakis Hexahedron | 14.32 | 30.91 | [4] 1.5910 | 1.18 | 1.03 | 1.5000 | 0.99 | 0.91 | 1.4230 | 0.84 | 0.82 |
| | | | | [6] 1.8371 | 1.81 | 1.37 | | | | | | |
| 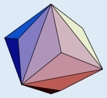 | Triakis Octahedron | 23.31 | 42.69 | [3] 1.7321 | 0.93 | 0.88 | 1.7072 | 0.89 | 0.86 | 1.6383 | 0.79 | 0.79 |
| | | | | [8] 2.2414 | 2.02 | 1.48 | | | | | | |
| 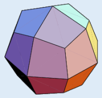 | Deltoidal Icositetrahedron | 8.7507 | 21.51 | [3] 1.3397 | 1.15 | 1.05 | 1.3066 | 1.07 | 1.00 | 1.2203 | 0.87 | 0.87 |
| | | | | [4] 1.4142 | 1.35 | 1.17 | | | | | | |

| | | | | | | | | | | | |
|---|---|---|---|---|---|---|---|---|---|---|---|
| 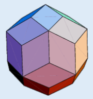 Rhombic Triacontahedron | 14.80 | 30.34 | [3] 1.5667 | 1.09 | 1.02 | 1.5388 | 1.03 | 0.98 | 1.4635 | 0.89 | 0.89 |
| | | | [5] 1.7204 | 1.44 | 1.23 | | | | | | |
| 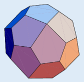 Pentagonal Icositetrahedron | 7.4474 | 19.30 | [3] 1.2820 | 1.19 | 1.07 | 1.2472 | 1.09 | 1.01 | 1.1577 | 0.87 | 0.87 |
| | | | [4] 1.3614 | 1.42 | 1.21 | | | | | | |
| 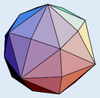 Disdyakis Dodecahedron | 46.66 | 67.42 | [4] 2.3204 | 1.12 | 1.00 | 2.2630 | 1.04 | 0.95 | 2.2097 | 0.97 | 0.91 |
| | | | [6] 2.4495 | 1.32 | 1.12 | | | | | | |
| | | | [8] 2.6754 | 1.72 | 1.33 | | | | | | |
| 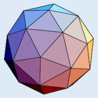 Pentakis Dodecahedron | 59.88 | 75.56 | [5] 2.5309 | 1.13 | 1.07 | 2.4271 | 1.00 | 0.98 | 2.3771 | 0.94 | 0.94 |
| | | | [6] 2.5981 | 1.23 | 1.12 | | | | | | |
| 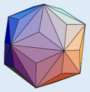 Triakis Icosahedron | 111.15 | 115.57 | [3] 2.9414 | 0.96 | 0.94 | 2.9271 | 0.95 | 0.93 | 2.8853 | 0.91 | 0.91 |
| | | | [10] 3.4410 | 1.54 | 1.29 | | | | | | |

| | | | | | | | | | | | |
|---|---|---|---|---|---|---|---|---|---|---|---|
| 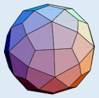 Deltoidal Hexecontahedron | 42.26 | 59.77 | [3] 2.1957 | 1.05 | 1.01 | 2.1763 | 1.02 | 1.00 | 2.1210 | 0.95 | 0.95 |
| | | | [4] 2.2361 | 1.11 | 1.05 | | | | | | |
| | | | [5] 2.2940 | 1.20 | 1.11 | | | | | | |
| 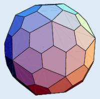 Pentagonal Hexecontahedron | 37.59 | 55.28 | [3] 2.1172 | 1.06 | 1.02 | 2.0971 | 1.03 | 1.00 | 2.0399 | 0.95 | 0.95 |
| | | | [5] 2.2000 | 1.19 | 1.10 | | | | | | |
| 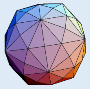 Disdyakis Triacontahedron | 7.20 | [4] 1.89 | 7.14 | 7.07 | 1548.4 | 91.1 | 92.0 | 89.6 | 87.2 | 1.010 | 0.983 |
| | | [6] 1.86 | 7.01 | 6.95 | 1466.0 | 86.2 | 92.0 | 84.8 | 82.6 | 1.066 | 0.983 |
| | | [10] 1.74 | 6.57 | 6.52 | 1209.8 | 71.2 | 92.0 | 70.0 | 68.2 | 1.292 | 0.983 |

**Section S2 – Data for polyhedra**

**Table S2A.** Calculated numbers of gold atoms in various polyhedra assuming a radius of the circumscribed sphere of **10.08 Å** (*i.e.* a $Au_{147}$ cluster with regular icosahedral shape) and ratios between the number of gold atoms in the volume described by the circumscribed sphere (midscribed sphere or edge-scribed sphere) and the number of gold atoms obtained for each polyhedron

assuming a sphere with vertex radius $N_c$ / $N_v$, $N_m$ / $N_v$ or $N_i$ / $N_v$. $N_c$ is the number of gold atoms contained within the circumscribed sphere, $N_v$ the number of gold atoms calculated from the volume of the polyhedron, and $N_{ve}$ is the number of gold atoms calculated from the volume of the sphere with vertex radius in Catalan solids ($N_{ve} = V_{Rv} / V_{Au}$), $N_m$ is the number of gold atoms calculated from the volume of the sphere with midscribed radius ($N_m = V_{Rm} / V_{Au}$), and $N_e$ is the number of gold atoms calculated from the volume of the sphere with edge-scribed radius ($N_{es} = V_{Re} / V_{Au}$). $R_i$ = radius of the inscribed sphere, $R_m$ = radius of the midscribed sphere, $R_e$ = radius of the edge-scribed sphere, $R_v$ = vertex radius (numbers in square brackets denote faces of polygons, *e.g.*, [3] for triangle, [5] for pentagon, *etc.*), $V$ is the calculated volume of the polyhedron, $L$ is the edge length of the polyhedron, a is the edge length of dual solid in Catalan solids, and $N_i$ is the number of gold atoms in the inscribed sphere. Models taken from: Polyèdres, http://wwwmathcurvecom/polyedres/polyedresshtml

**⇣ Platonic and Archimedean Solids ⇣**

| | Polyhedron | $R_c$ / Å | $L$ / Å | $R_m$ / Å | $R_i$ / Å | $V$ / Å$^3$ | $N_v$ | $N_c$ | $N_m$ | $N_i$ | $N_c$ / $N_v$ | $N_m$ / $N_v$ | $N_i$ / $N_v$ |
|---|---|---|---|---|---|---|---|---|---|---|---|---|---|
| 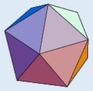 | Icosahedron | 10.08 | 10.60 | 8.57 | 8.01 | 2597.51 | 152.8 | 252.4 | 155.3 | 126.6 | 1.65 | 1.02 | 0.83 |
| 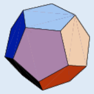 | Dodecahedron | 10.08 | 7.19 | 9.42 | 8.01 | 2852.25 | 167.8 | 252.4 | 205.7 | 126.6 | 1.50 | 1.23 | 0.75 |
| 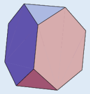 | Truncated Tetrahedron | 10.08 | 8.60 | 9.12 | [3] 8.77 | 1722.11 | 101.3 | 252.4 | 186.7 | 166.4 | 2.49 | 1.84 | 1.64 |
| | | | | | [6] 5.26 | | | | | 35.9 | | | 0.35 |
| 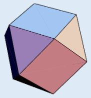 | Cuboctahedron | 10.08 | 10.08 | 8.73 | [3] 8.23 | 2414.02 | 142.0 | 252.4 | 163.9 | 137.4 | 1.78 | 1.15 | 0.97 |

| | | | | | | | | | | | | |
|---|---|---|---|---|---|---|---|---|---|---|---|---|
| | | | | [4] 7.13 | | | | | 89.2 | | | 0.63 |
| 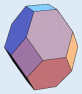 Truncated Octahedron | 10.08 | 6.38 | 9.56 | [4] 9.02 | 2930.67 | 172.4 | 252.4 | 215.5 | 180.6 | 1.46 | 1.25 | 1.05 |
| | | | | [6] 7.81 | | | | | 117.3 | | | 0.68 |
| 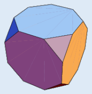 Truncated Cube | 10.08 | 5.67 | 9.67 | [3] 9.53 | 2467.10 | 145.1 | 252.4 | 223.0 | 213.5 | 1.74 | 1.54 | 1.47 |
| | | | | [8] 6.84 | | | | | 78.8 | | | 0.54 |
| 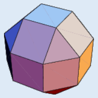 Rhombicuboctahedron | 10.08 | 7.21 | 9.41 | [3] 9.18 | 3259.46 | 191.7 | 252.4 | 205.6 | 190.7 | 1.32 | 1.07 | 0.99 |
| | | | | [4] 8.70 | | | | | 162.1 | | | 0.85 |
| 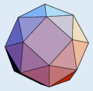 Snub Cube | 10.08 | 7.50 | 9.36 | [3] 9.10 | 3330.61 | 195.9 | 252.4 | 201.8 | 185.8 | 1.29 | 1.03 | 0.95 |
| | | | | [4] 8.57 | | | | | 155.2 | | | 0.79 |
| 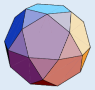 Icosidodecahedron | 10.08 | 6.23 | 9.59 | [3] 9.42 | 3346.43 | 196.8 | 252.4 | 217.1 | 205.7 | 1.28 | 1.10 | 1.05 |
| | | | | [5] 8.57 | | | | | 155.4 | | | 0.79 |

| | | | | | | | | | | | | |
|---|---|---|---|---|---|---|---|---|---|---|---|---|
| 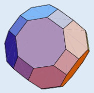 Truncated Cuboctahedron | 10.08 | 4.35 | 9.84 | [4] 9.60 | 3438.26 | 202.3 | 252.4 | 234.9 | 218.0 | 1.25 | 1.16 | 1.08 |
| | | | | [6] 9.09 | | | | | 185.3 | | | 0.92 |
| | | | | [8] 8.33 | | | | | 142.2 | | | 0.70 |
| 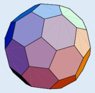 Truncated Icosahedron | 10.08 | 4.07 | 9.87 | [5] 9.47 | 3721.55 | 218.9 | 252.4 | 237.1 | 209.1 | 1.15 | 1.08 | 0.96 |
| | | | | [6] 9.22 | | | | | 193.3 | | | 0.88 |
| 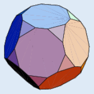 Truncated Dodecahedron | 10.08 | 3.39 | 9.94 | [3] 9.89 | 3326.25 | 195.7 | 252.4 | 241.7 | 238.2 | 1.29 | 1.24 | 1.22 |
| | | | | [10] 8.48 | | | | | 150.4 | | | 0.77 |
| 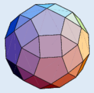 Rhombicosido-decahedron | 10.08 | 4.51 | 9.82 | [3] 9.74 | 3828.40 | 225.2 | 252.4 | 233.6 | 227.5 | 1.12 | 1.04 | 1.01 |
| | | | | [4] 9.56 | | | | | 215.3 | | | 0.96 |
| | | | | [5] 9.32 | | | | | 199.7 | | | 0.89 |
| 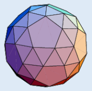 Snub Dodecahedron | 10.08 | 4.68 | 9.81 | [3] 9.71 | 3845.70 | 226.2 | 252.4 | 232.3 | 225.7 | 1.12 | 1.03 | 1.00 |

| | | | | | | | | | | | | | |
|---|---|---|---|---|---|---|---|---|---|---|---|---|---|
| | | | | | [5] 9.26 | | | | | 195.8 | | | 0.87 |
| 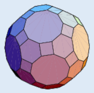 | Truncated Icosidodecahedron | 10.08 | 2.65 | 9.99 | [4] 9.90 | 3852.64 | 226.6 | 252.4 | 245.8 | 239.4 | 1.11 | 1.08 | 1.06 |
| | | | | | [6] 9.73 | | | | | 226.6 | | | 1.00 |

**⇣ Catalan Solids ⇣**

| | **Polyhedron** | $R_v$ / Å | $a$ / Å | $R_e$ / Å | $R_i$ / Å | $V$ / Å$^3$ | $N_v$ | $N_{ve}$ | $N_e$ | $N_i$ | $N_{ve}$ / $N_v$ | $N_{es}$ / $N_v$ | $N_i$ / $N_v$ |
|---|---|---|---|---|---|---|---|---|---|---|---|---|---|
| 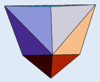 | Triakis Tetrahedron | 10.80 | [3] 9.14 | 9.70 | 8.77 | 4379.81 | 257.6 | 252.4 | 224.9 | 166.4 | 0.980 | 0.873 | 0.646 |
| | | | [6] 5.49 | 5.82 | 5.26 | 946.14 | 55.7 | 252.4 | 48.6 | 35.9 | 4.534 | 0.873 | 0.646 |
| 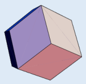 | Rhombic Dodecahedron | 10.80 | [3] 10.97 | 10.35 | 8.23 | 3153.29 | 185.5 | 252.4 | 272.8 | 137.3 | 1.361 | 1.471 | 0.740 |
| | | | [4] 9.50 | 8.96 | 7.13 | 2048.17 | 120.5 | 252.4 | 177.2 | 89.2 | 2.095 | 1.471 | 0.740 |
| 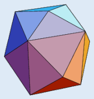 | Tetrakis Hexahedron | 10.80 | [4] 6.34 | 9.50 | 9.02 | 3641.78 | 214.2 | 252.4 | 211.5 | 180.6 | 1.178 | 0.987 | 0.843 |
| | | | [6] 5.49 | 8.23 | 7.81 | 2365.52 | 139.1 | 252.4 | 137.4 | 117.3 | 1.814 | 0.987 | 0.843 |

| | | | | | | | | | | | | |
|---|---|---|---|---|---|---|---|---|---|---|---|---|
| 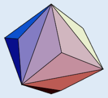 Triakis Octahedron | 10.80 | [3] 5.82 | 9.94 | 9.53 | 4594.15 | 270.2 | 252.4 | 241.6 | 213.5 | 0.934 | 0.894 | 0.790 |
| | | [8] 4.50 | 7.68 | 7.37 | 2120.09 | 124.7 | 252.4 | 111.5 | 98.5 | 2.024 | 0.894 | 0.790 |
| 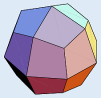 Deltoidal Icositetrahedron | 10.80 | [3] 7.52 | 9.83 | 9.18 | 3727.36 | 219.3 | 252.4 | 234.1 | 190.7 | 1.151 | 1.068 | 0.870 |
| | | [4] 7.13 | 9.31 | 8.70 | 3168.78 | 186.4 | 252.4 | 199.0 | 162.1 | 1.354 | 1.068 | 0.870 |
| 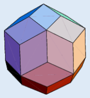 Rhombic Triacontahedron | 10.80 | [3] 6.43 | 9.90 | 9.42 | 3941.72 | 231.9 | 252.4 | 239.1 | 205.7 | 1.088 | 1.031 | 0.887 |
| | | [5] 5.86 | 9.02 | 8.57 | 2976.84 | 175.1 | 252.4 | 180.6 | 155.3 | 1.441 | 1.031 | 0.887 |
| 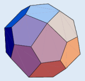 Pentagonal Icositetrahedron | 10.80 | [3] 7.86 | 9.81 | 9.10 | 3620.11 | 212.9 | 252.4 | 232.4 | 185.8 | 1.185 | 1.091 | 0.873 |
| | | [4] 7.40 | 9.23 | 8.57 | 3022.94 | 177.8 | 252.4 | 194.0 | 155.2 | 1.419 | 1.091 | 0.873 |
| 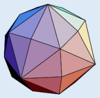 Disdyakis Dodecahedron | 10.80 | [4] 4.34 | 9.83 | 9.60 | 3825.37 | 225.0 | 252.4 | 234.1 | 217.9 | 1.121 | 1.040 | 0.969 |
| | | [6] 4.12 | 9.31 | 9.09 | 3251.84 | 191.3 | 252.4 | 199.0 | 185.3 | 1.319 | 1.040 | 0.969 |
| | | [8] 3.77 | 8.53 | 8.33 | 2495.72 | 146.8 | 252.4 | 152.7 | 142.2 | 1.719 | 1.040 | 0.969 |

| | | | | | | | | | | | | |
|---|---|---|---|---|---|---|---|---|---|---|---|---|
| 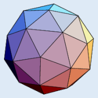 Pentakis Dodecahedron | 10.80 | [5] 3.98 | 9.67 | 9.47 | 3783.02 | 222.5 | 252.4 | 222.6 | 209.1 | 1.134 | 1.000 | 0.940 |
| | | [6] 3.88 | 9.42 | 9.22 | 3497.00 | 205.7 | 252.4 | 205.7 | 193.3 | 1.227 | 1.000 | 0.940 |
| 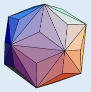 Triakis Icosahedron | 10.80 | [3] 3.43 | 10.03 | 9.89 | 4473.31 | 263.1 | 252.4 | 248.7 | 238.2 | 0.959 | 0.945 | 0.905 |
| | | [10] 2.93 | 8.57 | 8.45 | 2794.07 | 164.4 | 252.4 | 155.3 | 148.8 | 1.535 | 0.945 | 0.905 |
| 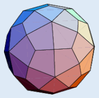 Deltoidal Hexecontahedron | 10.80 | [3] 4.59 | 9.99 | 9.74 | 4088.76 | 240.5 | 252.4 | 245.7 | 227.5 | 1.049 | 1.022 | 0.946 |
| | | [4] 4.51 | 9.81 | 9.56 | 3871.13 | 227.7 | 252.4 | 232.7 | 215.4 | 1.108 | 1.022 | 0.946 |
| | | [5] 4.39 | 9.56 | 9.32 | 3585.34 | 210.9 | 252.4 | 215.5 | 199.5 | 1.197 | 1.022 | 0.946 |
| 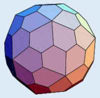 Pentagonal Hexecontahedron | 10.80 | [3] 4.76 | 9.98 | 9.71 | 4056.66 | 238.6 | 252.4 | 245.2 | 225.7 | 1.058 | 1.028 | 0.946 |
| | | [5] 4.58 | 9.61 | 9.35 | 3615.65 | 212.7 | 252.4 | 218.6 | 201.2 | 1.187 | 1.028 | 0.946 |
| 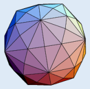 Disdyakis Triacontahedron | 10.80 | [4] 2.65 | 9.99 | 9.90 | 4248.94 | 249.9 | 252.4 | 245.7 | 239.4 | 1.010 | 0.983 | 0.958 |
| | | [6] 2.60 | 9.81 | 9.73 | 4022.69 | 236.6 | 252.4 | 232.6 | 226.6 | 1.066 | 0.983 | 0.958 |

| | | | | | | | | | | | | |
|---|---|---|---|---|---|---|---|---|---|---|---|---|
| | | [10] 2.44 | 9.20 | 9.12 | 3319.66 | 195.3 | 252.4 | 192.0 | 187.0 | 1.292 | 0.983 | 0.958 |

**Table S2B.** Calculated numbers of gold atoms in various polyhedra assuming a radius of the circumscribed sphere of **7.20 Å** (*i.e.* a $Au_{55}$ cluster with regular icosahedral shape) and ratios between the number of gold atoms in the volume described by the circumscribed sphere (midscribed sphere or edge-scribed sphere) and the number of gold atoms obtained for each polyhedron assuming a sphere with vertex radius $N_c / N_v$, $N_m / N_v$ or $N_i / N_v$. $N_c$ is the number of gold atoms contained within the circumscribed sphere, $N_v$ the number of gold atoms calculated from the volume of the polyhedron, and $N_{ve}$ is the number of gold atoms calculated from the volume of the sphere with vertex radius in Catalan solids ($N_{ve} = V_{Rv} / V_{Au}$), $N_m$ is the number of gold atoms calculated from the volume of the sphere with midscribed radius ($N_m = V_{Rm} / V_{Au}$), and $N_e$ is the number of gold atoms calculated from the volume of the sphere with edge-scribed radius ($N_e = V_{Re} / V_{Au}$). $R_i$ = radius of the inscribed sphere, $R_m$ = radius of the midscribed sphere, $R_e$ = radius of the edge-scribed sphere, $R_v$ = vertex radius (numbers in square brackets denote faces of polygons, *e.g.*, [3] for triangle, [5] for pentagon, etc.), $V$ is the calculated volume of the polyhedron, $L$ is the edge length of the polyhedron, a is the edge length of dual solid in Catalan solids, and $N_i$ is the number of gold atoms in the inscribed sphere.[S1]

**⇣ Platonic and Archimedean Solids ⇣**

| Polyhedron | $R_c$ / Å | $L$ / Å | $R_m$ / Å | $R_i$ / Å | $V$ / Å$^3$ | $N_v$ | $N_c$ | $N_m$ | $N_i$ | $N_c / N_v$ | $N_m / N_v$ | $N_i / N_v$ |
|---|---|---|---|---|---|---|---|---|---|---|---|---|
| 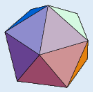 Icosahedron | 7.20 | 7.57 | 6.12 | 5.72 | 946.62 | 55.7 | 92.0 | 56.6 | 46.2 | 1.652 | 1.016 | 0.829 |
| 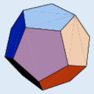 Dodecahedron | 7.20 | 5.14 | 6.73 | 5.72 | 1039.45 | 61.1 | 92.0 | 75.0 | 46.1 | 1.504 | 1.226 | 0.755 |

| | | | | | | | | | | | | |
|---|---|---|---|---|---|---|---|---|---|---|---|---|
| 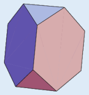 Truncated Tetrahedron | 7.20 | 6.14 | 6.51 | [3] 6.27 | 627.59 | 36.9 | 92.0 | 68.1 | 60.6 | 2.491 | 1.843 | 1.643 |
| | | | | [6] 3.76 | | | | | 13.1 | | | 0.355 |
| 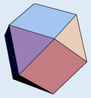 Cuboctahedron | 7.20 | 7.20 | 6.24 | [3] 5.88 | 879.75 | 51.7 | 92.0 | 59.7 | 50.1 | 1.777 | 1.154 | 0.967 |
| | | | | [4] 5.09 | | | | | 32.5 | | | 0.628 |
| 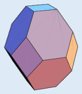 Truncated Octahedron | 7.20 | 4.55 | 6.83 | [4] 6.44 | 1068.03 | 62.8 | 92.0 | 78.5 | 65.8 | 1.464 | 1.250 | 1.048 |
| | | | | [6] 5.58 | | | | | 42.7 | | | 0.680 |
| 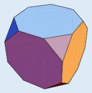 Truncated Cube | 7.20 | 4.05 | 6.91 | [3] 6.81 | 899.09 | 52.9 | 92.0 | 81.3 | 77.8 | 1.739 | 1.537 | 1.471 |
| | | | | [8] 4.89 | | | | | 28.7 | | | 0.543 |
| 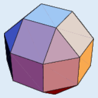 Rhombicuboctahedron | 7.20 | 5.15 | 6.72 | [3] 6.56 | 1187.85 | 69.9 | 92.0 | 74.9 | 69.5 | 1.316 | 1.072 | 0.995 |
| | | | | [4] 6.21 | | | | | 59.1 | | | 0.845 |
| 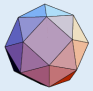 Snub Cube | 7.20 | 5.36 | 6.68 | [3] 6.50 | 1213.78 | 71.4 | 92.0 | 73.5 | 67.7 | 1.288 | 1.030 | 0.949 |

| | | | | | | | | | | | | |
|---|---|---|---|---|---|---|---|---|---|---|---|---|
| | | | | [4] 6.12 | | | | | 56.5 | | | 0.792 |
| 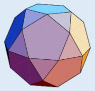 Icosidodecahedron | 7.20 | 4.45 | 6.85 | [3] 6.73 | 1219.55 | 71.7 | 92.0 | 79.1 | 75.0 | 1.282 | 1.103 | 1.045 |
| | | | | [5] 6.12 | | | | | 56.6 | | | 0.789 |
| 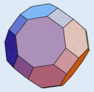 Truncated Cuboctahedron | 7.20 | 4.35 | 7.03 | [4] 6.86 | 1253.01 | 73.7 | 92.0 | 85.6 | 79.4 | 1.248 | 1.162 | 1.078 |
| | | | | [6] 6.50 | | | | | 67.5 | | | 0.916 |
| | | | | [8] 5.95 | | | | | 51.8 | | | 0.703 |
| 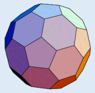 Truncated Icosahedron | 7.20 | 4.07 | 7.05 | [5] 6.76 | 1356.25 | 79.8 | 92.0 | 86.4 | 76.2 | 1.153 | 1.083 | 0.955 |
| | | | | [6] 6.59 | | | | | 70.4 | | | 0.883 |
| 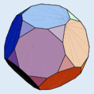 Truncated Dodecahedron | 7.20 | 3.39 | 7.10 | [3] 7.06 | 1212.19 | 71.3 | 92.0 | 88.1 | 86.8 | 1.290 | 1.235 | 1.217 |
| | | | | [10] 6.06 | | | | | 54.8 | | | 0.769 |
| 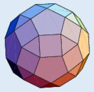 Rhombicosido-decahedron | 7.20 | 4.51 | 7.02 | [3] 6.96 | 1395.19 | 82.1 | 92.0 | 85.1 | 82.9 | 1.121 | 1.037 | 1.010 |

| | | | | | | | | | | | | |
|---|---|---|---|---|---|---|---|---|---|---|---|---|
| | | | | [4] 6.83 | | | | | 78.5 | | | 0.956 |
| | | | | [5] 6.66 | | | | | 72.8 | | | 0.887 |
| 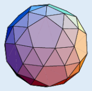 Snub Dodecahedron | 7.20 | 4.68 | 7.00 | [3] 6.94 | 1401.49 | 82.4 | 92.0 | 84.7 | 82.3 | 1.116 | 1.027 | 0.998 |
| | | | | [5] 6.62 | | | | | 71.4 | | | 0.865 |
| 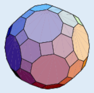 Truncated Icosidodecahedron | 7.20 | 2.65 | 7.14 | [4] 7.07 | 1404.02 | 82.6 | 92.0 | 89.6 | 87.2 | 1.114 | 1.085 | 1.056 |
| | | | | [6] 6.95 | | | | | 82.6 | | | 1.000 |

**⇣ Catalan Solids ⇣**

| **Polyhedron** | $R_v$ / Å | $a$ / Å | $R_e$ / Å | $R_i$ / Å | $V$ / Å$^3$ | $N_v$ | $N_{ve}$ | $N_e$ | $N_i$ | $N_{ve}$ / $N_v$ | $N_{es}$ / $N_v$ | $N_i$ / $N_v$ |
|---|---|---|---|---|---|---|---|---|---|---|---|---|
| 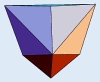 Triakis Tetrahedron | 7.20 | [3] 6.53 | 6.93 | 6.27 | 1596.1 | 93.9 | 92.0 | 81.9 | 60.6 | 0.980 | 0.873 | 0.646 |
| | | [6] 3.92 | 4.16 | 3.76 | 344.8 | 20.3 | 92.0 | 17.7 | 13.1 | 4.534 | 0.873 | 0.646 |
| 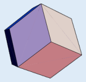 Rhombic Dodecahedron | 7.20 | [3] 7.84 | 7.39 | 5.88 | 1149.2 | 67.6 | 92.0 | 99.4 | 50.1 | 1.361 | 1.471 | 0.740 |

| | | | | | | | | | | | | |
|---|---|---|---|---|---|---|---|---|---|---|---|---|
| | | [4] 6.79 | 6.40 | 5.09 | 746.4 | 43.9 | 92.0 | 64.6 | 32.5 | 2.095 | 1.471 | 0.740 |
| 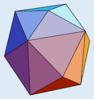 Tetrakis Hexahedron | 7.20 | [4] 4.53 | 6.79 | 6.44 | 1327.2 | 78.1 | 92.0 | 77.1 | 65.8 | 1.178 | 0.987 | 0.843 |
| | | [6] 3.92 | 5.88 | 5.58 | 862.1 | 50.7 | 92.0 | 50.1 | 42.7 | 1.814 | 0.987 | 0.843 |
| 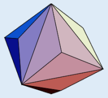 Triakis Octahedron | 7.20 | [3] 4.16 | 7.10 | 6.81 | 1674.3 | 98.5 | 92.0 | 88.1 | 77.8 | 0.934 | 0.894 | 0.790 |
| | | [8] 3.21 | 5.48 | 5.26 | 772.6 | 45.4 | 92.0 | 40.6 | 35.9 | 2.024 | 0.894 | 0.790 |
| 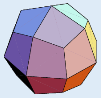 Deltoidal Icositetrahedron | 7.20 | [3] 5.37 | 7.02 | 6.56 | 1358.4 | 79.9 | 92.0 | 85.3 | 69.5 | 1.151 | 1.068 | 0.870 |
| | | [4] 5.09 | 6.65 | 6.21 | 1154.8 | 67.9 | 92.0 | 72.5 | 59.1 | 1.354 | 1.068 | 0.870 |
| 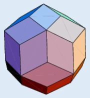 Rhombic Triacontahedron | 7.20 | [3] 4.60 | 7.07 | 6.73 | 1436.5 | 84.5 | 92.0 | 87.1 | 75.0 | 1.088 | 1.031 | 0.887 |
| | | [5] 4.19 | 6.44 | 6.12 | 1084.9 | 63.8 | 92.0 | 65.8 | 56.6 | 1.441 | 1.031 | 0.887 |
| 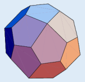 Pentagonal Icositetrahedron | 7.20 | [3] 5.62 | 7.00 | 6.50 | 1319.3 | 77.6 | 92.0 | 84.7 | 67.7 | 1.185 | 1.091 | 0.873 |
| | | [4] 5.29 | 6.60 | 6.12 | 1101.7 | 64.8 | 92.0 | 70.7 | 56.6 | 1.419 | 1.091 | 0.873 |

| | | | | | | | | | | | | | | |
|---|---|---|---|---|---|---|---|---|---|---|---|---|---|---|
| 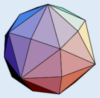 | Disdyakis Dodecahedron | 7.20 | [4] 3.10 | 7.02 | 6.86 | 1394.1 | 82.0 | 92.0 | 85.3 | 79.4 | 1.121 | 1.040 | 0.969 |
| | | | [6] 2.94 | 6.65 | 6.50 | 1185.1 | 69.7 | 92.0 | 72.5 | 67.5 | 1.319 | 1.040 | 0.969 |
| | | | [8] 2.69 | 6.09 | 5.95 | 909.5 | 53.5 | 92.0 | 55.7 | 51.8 | 1.719 | 1.040 | 0.969 |
| 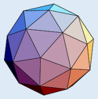 | Pentakis Dodecahedron | 7.20 | [5] 2.84 | 6.90 | 6.76 | 1378.7 | 81.1 | 92.0 | 81.1 | 76.2 | 1.134 | 1.000 | 0.940 |
| | | | [6] 2.77 | 6.73 | 6.59 | 1274.4 | 75.0 | 92.0 | 75.0 | 70.4 | 1.227 | 1.000 | 0.940 |
| 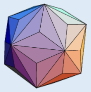 | Triakis Icosahedron | 7.20 | [3] 2.45 | 7.16 | 7.06 | 1630.2 | 95.9 | 92.0 | 90.6 | 86.8 | 0.959 | 0.945 | 0.905 |
| | | | [10] 2.09 | 6.12 | 6.04 | 1018.2 | 59.9 | 92.0 | 56.6 | 54.2 | 1.535 | 0.945 | 0.905 |
| 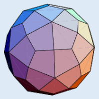 | Deltoidal Hexecontahedron | 7.20 | [3] 3.28 | 7.14 | 6.96 | 1490.1 | 87.7 | 92.0 | 89.6 | 82.9 | 1.049 | 1.022 | 0.946 |
| | | | [4] 3.22 | 7.01 | 6.83 | 1410.8 | 83.0 | 92.0 | 84.8 | 78.5 | 1.108 | 1.022 | 0.946 |
| | | | [5] 3.14 | 6.83 | 6.66 | 1306.6 | 76.9 | 92.0 | 78.5 | 72.7 | 1.197 | 1.022 | 0.946 |
| 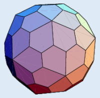 | Pentagonal Hexecontahedron | 7.20 | [3] 3.40 | 7.13 | 6.94 | 1478.4 | 87.0 | 92.0 | 89.4 | 82.3 | 1.058 | 1.028 | 0.946 |

| | | | | | | | | | | | | |
|---|---|---|---|---|---|---|---|---|---|---|---|---|
| | | [5] 3.27 | 6.86 | 6.68 | 1317.7 | 77.5 | 92.0 | 79.7 | 73.3 | 1.187 | 1.028 | 0.946 |
| 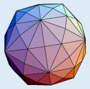 Disdyakis Triacontahedron | 7.20 | [4] 1.89 | 7.14 | 7.07 | 1548.4 | 91.1 | 92.0 | 89.6 | 87.2 | 1.010 | 0.983 | 0.958 |
| | | [6] 1.86 | 7.01 | 6.95 | 1466.0 | 86.2 | 92.0 | 84.8 | 82.6 | 1.066 | 0.983 | 0.958 |
| | | [10] 1.74 | 6.57 | 6.52 | 1209.8 | 71.2 | 92.0 | 70.0 | 68.2 | 1.292 | 0.983 | 0.958 |

**Section S3 – Data for Archimedean Icosahedra**

**Table S3.** List of Archimedean icosahedron clusters (for abbreviations see Table 4 in the main text).

| **Polyhedron** | ***G*** | **Cluster** | **Surface** | $D_c$ / nm | $D_m$ / nm | $D_i$ / nm | $N_v$ | $N_c$ | $N_m$ | $N_i$ | $N_v / N$ | $N_c / N$ | $N_m / N$ | $N_i / N$ |
|---|---|---|---|---|---|---|---|---|---|---|---|---|---|---|
| Icosahedron 13 | 1 | 13 | 12 | 0.864 | 0.735 | 0.687 | 12.0 | 19.9 | 12.2 | 10.0 | 0.93 | 1.53 | 0.94 | 0.77 |
| Dodecahedron 33 | 2 | 33 | 20 | 1.203 | 1.124 | 0.956 | 35.7 | 53.7 | 43.8 | 26.9 | 1.08 | 1.63 | 1.33 | 0.82 |
| Icosidodecahedron 43 | 2 | 43 | 30 | 1.268 | 1.206 | [3] 1.184 | 49.0 | 62.8 | 54.0 | 51.2 | 1.14 | 1.46 | 1.26 | 1.19 |
| | | | | | | [4] 1.079 | | | | 38.6 | | | | 0.90 |
| Icosahedron 55 | 2 | 55 | 42 | 1.440 | 1.225 | 1.144 | 55.7 | 92.0 | 56.6 | 46.2 | 1.01 | 1.67 | 1.03 | 0.84 |
| Dodecahedron 75 | 3 | 75 | 32 | 1.680 | 1.570 | 1.335 | 97.1 | 146.1 | 119.1 | 73.3 | 1.30 | 1.95 | 1.59 | 0.98 |
| Rhombicosido- | 3 | 115 | 60 | 1.717 | 1.674 | [3] 1.659 | 139.2 | 156.0 | 144.4 | 140.6 | 1.21 | 1.36 | 1.26 | 1.22 |

| | | | | | | | | | | | | | | |
|---|---|---|---|---|---|---|---|---|---|---|---|---|---|---|
| decahedron 115 | | | | | | | | | | | | | | |
| | | | | | | [4] 1.629 | | | | 133.1 | | | | 1.16 |
| | | | | | | [5] 1.588 | | | | 123.4 | | | | 1.07 |
| Truncated Icosahedron 135 | 3 | 135 | 80 | 1.758 | 1.722 | [5] 1.651 | 145.1 | 167.3 | 157.2 | 138.6 | 1.07 | 1.24 | 1.16 | 1.03 |
| | | | | | | [6] 1.608 | | | | 128.1 | | | | 0.95 |
| Icosidodecahedron 145 | 3 | 145 | 90 | 1.867 | 1.776 | [3] 1.744 | 156.4 | 200.5 | 172.5 | 163.5 | 1.08 | 1.38 | 1.19 | 1.13 |
| | | | | | | [5] 1.588 | | | | 123.4 | | | | 0.85 |
| Icosahedron 147 | 3 | 147 | 92 | 2.016 | 1.715 | 1.602 | 152.8 | 252.4 | 155.3 | 126.6 | 1.04 | 1.72 | 1.06 | 0.86 |
| Dodecahedron 165 | 3 | 165 | 110 | 2.175 | 2.031 | 1.728 | 210.6 | 316.7 | 258.2 | 158.9 | 1.28 | 1.92 | 1.56 | 0.96 |
| Truncated Dodecahedron 195 | 4 | 195 | 140 | 2.119 | 2.089 | [3] 2.078 | 227.2 | 293.0 | 280.6 | 276.5 | 1.17 | 1.50 | 1.44 | 1.42 |
| | | | | | | [10] 1.783 | | | | 174.6 | | | | 0.90 |
| Rhombicosido-decahedron 207 | 4 | 207 | 72 | 2.119 | 2.065 | [3] 2.047 | 261.5 | 293.0 | 271.2 | 264.1 | 1.26 | 1.42 | 1.31 | 1.28 |
| | | | | | | [4] 2.010 | | | | 250.0 | | | | 1.21 |
| | | | | | | [5] 1.960 | | | | 231.8 | | | | 1.12 |
| Icosidodecahedron 237 | 4 | 237 | 102 | 2.268 | 2.157 | [3] 2.119 | 280.3 | 359.4 | 309.2 | 293.0 | 1.18 | 1.52 | 1.30 | 1.24 |
| | | | | | | [5] 1.930 | | | | 221.3 | | | | 0.93 |
| Sub-Rhombicosi- | 4 | 267 | 120 | 2.182 | 2.126 | [3] 2.107 | 285.4 | 319.8 | 296.0 | 288.3 | 1.07 | 1.20 | 1.11 | 1.08 |

| | | | | | | | | | | | | | | |
|---|---|---|---|---|---|---|---|---|---|---|---|---|---|---|
| dodecahedron 267 | | | | | | | | | | | | | | |
| | | | | | | [4] 2.069 | | | | 272.9 | | | | 1.02 |
| | | | | | | [5] 2.018 | | | | 253.0 | | | | 0.95 |
| Icosahedron 309 | 4 | 309 | 162 | 2.592 | 2.205 | 2.060 | 324.7 | 536.4 | 330.1 | 269.2 | 1.05 | 1.74 | 1.07 | 1.01 |
| Sub-Truncated Icosahedron 327 | 4 | 327 | 180 | 2.453 | 2.403 | [5] 2.304 | 394.4 | 454.7 | 427.2 | 376.7 | 1.21 | 1.39 | 1.31 | 1.15 |
| | | | | | | [6] 2.244 | | | | 348.2 | | | | 1.06 |
| Truncated Dodecahedron 357 | 5 | 357 | 120 | 2.627 | 2.589 | [3] 2.577 | 432.8 | 558.2 | 534.7 | 526.9 | 1.21 | 1.56 | 1.50 | 1.48 |
| | | | | | | [10] 2.211 | | | | 332.7 | | | | 0.93 |
| Sub-Rhombico-sidodecahedron 387 | 5 | 387 | 120 | 2.493 | 2.430 | [3] 2.409 | 426.0 | 477.4 | 441.9 | 430.3 | 1.10 | 1.23 | 1.14 | 1.11 |
| | | | | | | [4] 2.365 | | | | 407.3 | | | | 1.05 |
| | | | | | | [5] 2.306 | | | | 377.7 | | | | 0.98 |
| Truncated Dodecahedron 447 | 5 | 417 | 180 | 2.738 | 2.699 | [3] 2.686 | 490.0 | 632.0 | 605.4 | 596.5 | 1.18 | 1.52 | 1.45 | 1.43 |
| | | | | | | [10] 2.304 | | | | 376.7 | | | | 0.90 |
| Sub-Rhombico-sidodecahedron 429 | 5 | 429 | 132 | 2.667 | 2.600 | [3] 2.577 | 521.6 | 584.5 | 541.1 | 526.9 | 1.22 | 1.36 | 1.26 | 1.23 |
| | | | | | | [4] 2.530 | | | | 498.7 | | | | 1.16 |

| | | | | | | | | | | | | | | |
|---|---|---|---|---|---|---|---|---|---|---|---|---|---|---|
| | | | | | | [5] 2.467 | | | | 462.4 | | | | 1.08 |
| Truncated Icosido-decahedron 437 | 5 | 437 | 140 | 2.671 | 2.647 | [4] 2.624 | 526.9 | 586.7 | 571.5 | 556.5 | 1.21 | 1.34 | 1.31 | 1.27 |
| | | | | | | [6] 2.577 | | | | 526.9 | | | | 1.21 |
| | | | | | | [12] 2.417 | | | | 434.8 | | | | 0.99 |
| Dodecahedron 467 | 5 | 437 | 210 | 3.086 | 2.883 | 2.452 | 602.0 | 905.4 | 738.0 | 454.3 | 1.38 | 2.07 | 1.69 | 1.04 |
| Sub-Truncated Icosahedron 489 | 5 | 489 | 192 | 2.738 | 2.681 | [5] 2.571 | 548.2 | 632.0 | 593.8 | 523.6 | 1.12 | 1.29 | 1.21 | 1.07 |
| | | | | | | [6] 2.505 | | | | 484.0 | | | | 0.99 |
| Sub-Icosahedron 509 | 5 | 509 | 200 | 3.028 | 2.576 | 2.407 | 517.9 | 855.4 | 526.4 | 429.3 | 1.02 | 1.68 | 1.03 | 0.84 |
| Icosahedron 561 | 5 | 561 | 252 | 3.168 | 2.695 | 2.517 | 592.9 | 979.3 | 602.7 | 491.4 | 1.06 | 1.75 | 1.07 | 0.88 |
| Sub-Truncated Icosahedron 599 | 5 | 599 | 290 | 2.918 | 2.858 | [5] 2.740 | 663.6 | 765.0 | 718.9 | 633.9 | 1.11 | 1.28 | 1.20 | 1.06 |
| | | | | | | [6] 2.670 | | | | 585.9 | | | | 0.98 |
| Snub Dodecahedron (enantiomer) 629 | 6 | 629 | 200 | 3.149 | 3.064 | [3] 3.034 | 862.5 | 962.2 | 885.7 | 860.6 | 1.37 | 1.53 | 1.41 | 1.37 |
| | | | | | | [5] 2.894 | | | | 746.5 | | | | 1.19 |

| | | | | | | | | | | | | | | |
|---|---|---|---|---|---|---|---|---|---|---|---|---|---|---|
| Truncated Dodecahedron 669 | 6 | 669 | 240 | 3.093 | 3.049 | [3] 3.034 | 706.9 | 911.7 | 873.2 | 860.5 | 1.06 | 1.36 | 1.31 | 1.29 |
| | | | | | | [10] 2.603 | | | | 543.4 | | | | 0.81 |
| Truncated Icosido-decahedron 689 | 6 | 689 | 260 | 3.064 | 3.037 | [4] 3.010 | 795.3 | 885.6 | 862.7 | 840.1 | 1.15 | 1.29 | 1.25 | 1.22 |
| | | | | | | [6] 2.956 | | | | 795.3 | | | | 1.15 |
| | | | | | | [12] 2.772 | | | | 656.3 | | | | 0.95 |
| Rhombicosido-decahedron 789 | 6 | 789 | 360 | 3.114 | 3.035 | [3] 3.008 | 829.8 | 929.9 | 860.9 | 838.3 | 1.05 | 1.18 | 1.09 | 1.06 |
| | | | | | | [4] 2.953 | | | | 793.5 | | | | 1.01 |
| | | | | | | [5] 2.880 | | | | 735.7 | | | | 0.93 |
| Sub-Truncated Icosidodecahedron 839 | 6 | 839 | 402 | 3.182 | 3.155 | [4] 3.127 | 891.5 | 992.8 | 967.1 | 941.7 | 1.06 | 1.18 | 1.15 | 1.12 |
| | | | | | | [6] 3.070 | | | | 891.5 | | | | 1.06 |
| | | | | | | [12] 2.880 | | | | 735.7 | | | | 0.88 |
| Truncated Icosahedron 851 | 6 | 851 | 302 | 3.228 | 3.161 | [5] 3.031 | 898.4 | 1034 | 973.1 | 858.1 | 1.06 | 1.22 | 1.14 | 1.01 |
| | | | | | | [6] 2.953 | | | | 793.2 | | | | 0.93 |
| Icosahedron 923 | 6 | 923 | 362 | 3.744 | 3.185 | 2.975 | 978.7 | 1616 | 994.8 | 811.1 | 1.06 | 1.75 | 1.08 | 0.88 |
| Dodecahedron 1019 | 6 | 1019 | 570 | 4.349 | 4.063 | 3.456 | 1685 | 2534 | 2066 | 1271 | 1.65 | 2.49 | 2.03 | 1.38 |

**Section S4 – Data for Archimedean Cubes**

**Table S4.** List of Archimedean cubes (for abbreviations see Table 5 in the main text).

| Polyhedron | $G$ | Cluster | Surface | $D_c$ / nm | $D_m$ / nm | $D_i$ / nm | $N_v$ | $N_c$ | $N_m$ | $N_i$ | $N_v / N$ | $N_c / N$ | $N_m / N$ | $N_i / N$ |
|---|---|---|---|---|---|---|---|---|---|---|---|---|---|---|
| Cuboctahedron 13 | 1 | 13 | 12 | 0.983 | 0.852 | [3] 0.803 | 16.5 | 29.3 | 19.0 | 15.9 | 1.27 | 2.25 | 1.46 | 1.23 |
| | | | | | | [4] 0.695 | | | | 10.4 | | | | 0.80 |
| Truncated Octahedron 38 | 2 | 38 | 32 | 1.233 | 1.169 | [4] 1.103 | 39.4 | 57.7 | 49.3 | 41.3 | 1.04 | 1.52 | 1.30 | 1.09 |
| | | | | | | [6] 0.955 | | | | 26.8 | | | | 0.71 |
| Rhombicuboctahedron 43 | 2 | 43 | 30 | 1.278 | 1.193 | [3] 1.164 | 48.8 | 64.3 | 52.4 | 48.6 | 1.14 | 1.49 | 1.22 | 1.13 |
| | | | | | | [4] 1.103 | | | | 41.3 | | | | 0.96 |
| Cuboctahedron 55 | 2 | 55 | 42 | 1.559 | 1.350 | [3] 1.273 | 65.7 | 116.8 | 75.8 | 63.6 | 1.19 | 2.12 | 1.38 | 1.16 |
| | | | | | | [4] 1.103 | | | | 41.3 | | | | 0.75 |
| Truncated Cube 62 | 2 | 62 | 48 | 1.625 | 1.559 | [3] 1.537 | 76.0 | 132.1 | 116.8 | 111.8 | 1.23 | 2.13 | 1.88 | 1.80 |
| | | | | | | [8] 1.103 | | | | 41.3 | | | | 0.67 |
| Sub-Truncated Octahedron 79 | 3 | 79 | 60 | 1.688 | 1.601 | [4] 1.510 | 101.2 | 148.2 | 126.5 | 106.0 | 1.28 | 1.88 | 1.60 | 1.34 |
| | | | | | | [6] 1.308 | | | | 68.9 | | | | 0.87 |
| Sub-Rhombicubocta-hedron 92 | 3 | 92 | 54 | 1.750 | 1.634 | [3] 1.594 | 125.4 | 165.0 | 134.5 | 124.7 | 1.36 | 1.79 | 1.46 | 1.36 |
| | | | | | | [4] 1.510 | | | | 106.0 | | | | 1.15 |

| | | | | | | | | | | | | | | |
|---|---|---|---|---|---|---|---|---|---|---|---|---|---|---|
| Snub Cube 111 | 3 | 111 | 56 | 1.776 | 1.648 | [3] 1.603 | 133.9 | 172.4 | 137.9 | 127.0 | 1.21 | 1.55 | 1.24 | 1.14 |
| | | | | | | [4] 1.510 | | | | 106.0 | | | | 0.96 |
| Sub-Truncated Octahedron 116 | 3 | 116 | 78 | 1.688 | 1.601 | [4] 1.510 | 101.2 | 148.2 | 126.5 | 106.0 | 0.87 | 1.28 | 1.09 | 0.91 |
| | | | | | | [6] 1.308 | | | | 68.9 | | | | 0.59 |
| Truncated Cuboctahedron 135 | 3 | 135 | 80 | 1.828 | 1.764 | [4] 1.741 | 150.8 | 188.2 | 169.0 | 162.5 | 1.12 | 1.39 | 1.25 | 1.20 |
| | | | | | | [6] 1.649 | | | | 138.2 | | | | 1.02 |
| | | | | | | [8] 1.510 | | | | 106.0 | | | | 0.79 |
| Sub-Rhombicubocta-hedron 140 | 3 | 140 | 78 | 1.750 | 1.634 | [3] 1.594 | 125.4 | 165.0 | 134.5 | 124.7 | 0.90 | 1.18 | 0.96 | 0.89 |
| | | | | | | [4] 1.510 | | | | 106.0 | | | | 0.76 |
| Cuboctahedron 147 | 3 | 147 | 92 | 2.135 | 1.849 | [3] 1.743 | 168.7 | 299.9 | 194.8 | 163.2 | 1.15 | 2.04 | 1.32 | 1.11 |
| | | | | | | [4] 1.510 | | | | 106.0 | | | | 0.72 |
| Truncated Cube 164 | 3 | 164 | 102 | 2.225 | 2.135 | [3] 2.105 | 195.1 | 339.3 | 299.9 | 287.1 | 1.19 | 2.07 | 1.83 | 1.75 |
| | | | | | | [8] 1.510 | | | | 106.0 | | | | 0.65 |
| Sub-Truncated Cube 171 | 3 | 171 | 108 | 2.225 | 2.135 | [3] 2.105 | 195.1 | 339.3 | 299.9 | 287.1 | 1.14 | 1.98 | 1.75 | 1.68 |
| | | | | | | [8] 1.510 | | | | 106.0 | | | | 0.62 |
| Sub-Truncated Octahedron 140 | 4 | 140 | 96 | 2.143 | 2.033 | [4] 1.917 | 207.2 | 303.3 | 259.0 | 217.0 | 1.48 | 2.17 | 1.85 | 1.55 |
| | | | | | | [6] 1.660 | | | | 141.0 | | | | 1.01 |

| | | | | | | | | | | | | | | |
|---|---|---|---|---|---|---|---|---|---|---|---|---|---|---|
| Sub-Rhombicubocta-hedron 165 | 4 | 165 | 86 | 2.222 | 2.075 | [3] 2.024 | 256.7 | 337.9 | 275.3 | 255.3 | 1.56 | 2.05 | 1.67 | 1.55 |
| | | | | | | [4] 1.917 | | | | 217.0 | | | | 1.32 |
| Truncated Octahedron 201 | 4 | 201 | 122 | 2.143 | 2.033 | [4] 1.917 | 207.2 | 303.3 | 259.0 | 217.0 | 1.03 | 1.51 | 1.29 | 1.08 |
| | | | | | | [6] 1.660 | | | | 141.0 | | | | 0.70 |
| Sub-Cuboctahedron 260 | 4 | 260 | 144 | 2.711 | 2.348 | [3] 2.214 | 345.4 | 613.9 | 398.7 | 334.2 | 1.33 | 2.36 | 1.53 | 1.29 |
| | | | | | | [4] 1.917 | | | | 217.0 | | | | 0.83 |
| Sub-Truncated Cuboctahedron 236 | 4 | 236 | 120 | 2.321 | 2.239 | [4] 2.211 | 308.7 | 385.2 | 345.9 | 332.7 | 1.31 | 1.63 | 1.47 | 1.41 |
| | | | | | | [6] 2.094 | | | | 282.8 | | | | 1.20 |
| | | | | | | [8] 1.917 | | | | 217.0 | | | | 0.92 |
| Sub-Rhombicubocta-hedron 249 | 4 | 249 | 114 | 2.222 | 2.075 | [3] 2.024 | 256.7 | 337.9 | 275.3 | 255.3 | 1.03 | 1.36 | 1.11 | 1.03 |
| | | | | | | [4] 1.917 | | | | 217.0 | | | | 0.87 |
| Sub-Snub Cube 249 | 4 | 249 | 114 | 2.255 | 2.093 | [3] 2.036 | 274.0 | 353.0 | 282.3 | 259.9 | 1.10 | 1.42 | 1.13 | 1.04 |
| | | | | | | [4] 1.917 | | | | 217.0 | | | | 0.87 |
| Sub-Snub Cube 268 | 4 | 268 | 128 | 2.255 | 2.093 | [3] 2.036 | 274.0 | 353.0 | 282.3 | 259.9 | 1.02 | 1.32 | 1.05 | 0.97 |
| | | | | | | [4] 1.917 | | | | 217.0 | | | | 0.81 |
| Sub-Truncated Cuboctahedron 297 | 4 | 297 | 162 | 2.321 | 2.239 | [4] 2.211 | 308.7 | 385.2 | 345.9 | 332.7 | 1.04 | 1.30 | 1.16 | 1.12 |

| | | | | | | | | | | | | | | |
|---|---|---|---|---|---|---|---|---|---|---|---|---|---|---|
| | | | | | | [6] 2.094 | | | | 282.8 | | | | 0.95 |
| | | | | | | [8] 1.917 | | | | 217.0 | | | | 0.73 |
| Cuboctahedron 309 | 4 | 309 | 162 | 2.711 | 2.348 | [3] 2.214 | 345.4 | 613.9 | 398.7 | 334.2 | 1.12 | 1.99 | 1.29 | 1.08 |
| | | | | | | [4] 1.917 | | | | 217.0 | | | | 0.70 |
| Sub-Truncated Cuboctahedron 316 | 4 | 316 | 152 | 2.321 | 2.239 | [4] 2.211 | 308.7 | 385.2 | 345.9 | 332.7 | 0.98 | 1.22 | 1.09 | 1.05 |
| | | | | | | [6] 2.094 | | | | 282.8 | | | | 0.90 |
| | | | | | | [8] 1.917 | | | | 217.0 | | | | 0.69 |
| Sub-Rhombicubocta-hedron 321 | 4 | 321 | 150 | 2.222 | 2.075 | [3] 2.024 | 256.7 | 337.9 | 275.3 | 255.3 | 0.80 | 1.05 | 0.86 | 0.80 |
| | | | | | | [4] 1.917 | | | | 217.0 | | | | 0.68 |
| Cuboctahedron 340 | 4 | 340 | 176 | 2.711 | 2.348 | [3] 2.214 | 345.4 | 613.9 | 398.7 | 334.2 | 1.02 | 1.81 | 1.17 | 0.98 |
| | | | | | | [4] 1.917 | | | | 217.0 | | | | 0.64 |
| Truncated Cube 357 | 4 | 357 | 186 | 2.825 | 2.711 | [3] 2.672 | 399.5 | 694.6 | 613.9 | 587.7 | 1.12 | 1.95 | 1.72 | 1.65 |
| | | | | | | [8] 1.917 | | | | 217.0 | | | | 0.61 |
| Sub-Cube 364 | 4 | 364 | 192 | 3.321 | 2.711 | 1.917 | 414.5 | 1127.8 | 613.9 | 217.0 | 1.14 | 3.10 | 1.69 | 0.60 |
| Sub-Octahedron 225 | 5 | 225 | 140 | 2.324 | 1.644 | 1.342 | 123.1 | 386.8 | 136.8 | 74.4 | 0.55 | 1.72 | 0.61 | 0.33 |
| Sub-Rhombicubocta-hedron 266 | 5 | 266 | 126 | 2.694 | 2.516 | [3] 2.454 | 457.5 | 602.2 | 490.5 | 455.1 | 1.72 | 2.26 | 1.84 | 1.71 |
| | | | | | | [4] 2.324 | | | | 386.8 | | | | 1.45 |
| Sub-Truncated Octahedron 314 | 5 | 314 | 174 | 2.599 | 2.465 | [4] 2.324 | 369.3 | 540.5 | 461.6 | 386.8 | 1.18 | 1.72 | 1.47 | 1.23 |

| | | | | | | | | | | | | | | |
|---|---|---|---|---|---|---|---|---|---|---|---|---|---|---|
| | | | | | | [6] 2.013 | | | | 251.2 | | | | 0.80 |
| Sub-Rhombicuboctahedron 369 | 5 | 369 | 168 | 2.694 | 2.516 | [3] 2.454 | 457.5 | 602.2 | 490.5 | 455.1 | 1.24 | 1.63 | 1.33 | 1.23 |
| | | | | | | [4] 2.324 | | | | 386.8 | | | | 1.05 |
| Rhombicuboctahedron 394 | 5 | 394 | 158 | 2.694 | 2.516 | [3] 2.454 | 457.5 | 602.2 | 490.5 | 455.1 | 1.16 | 1.53 | 1.25 | 1.15 |
| | | | | | | [4] 2.324 | | | | 386.8 | | | | 0.98 |
| Sub-Snub Cube 394 | 5 | 394 | 158 | 2.733 | 2.537 | [3] 2.468 | 488.4 | 629.1 | 503.0 | 463.2 | 1.24 | 1.60 | 1.28 | 1.18 |
| | | | | | | [4] 2.324 | | | | 386.8 | | | | 0.98 |
| Sub-Truncated Octahedron 405 | 5 | 405 | 204 | 2.599 | 2.465 | [4] 2.324 | 369.3 | 540.5 | 461.6 | 386.8 | 0.91 | 1.33 | 1.14 | 0.96 |
| | | | | | | [6] 2.013 | | | | 251.2 | | | | 0.62 |
| Snub Cube 429 | 5 | 429 | 132 | 2.733 | 2.537 | [3] 2.468 | 488.4 | 629.1 | 503.0 | 463.2 | 1.14 | 1.47 | 1.17 | 1.08 |
| | | | | | | [4] 2.324 | | | | 386.8 | | | | 0.90 |
| Sub-Truncated Cuboctahedron 466 | 5 | 466 | 230 | 2.814 | 2.715 | [4] 2.680 | 550.2 | 686.5 | 616.5 | 592.9 | 1.18 | 1.47 | 1.32 | 1.27 |
| | | | | | | [6] 2.539 | | | | 504.0 | | | | 1.08 |
| | | | | | | [8] 2.324 | | | | 386.8 | | | | 0.83 |
| Sub-Cuboctahedron 490 | 5 | 490 | 230 | 3.287 | 2.847 | [3] 2.684 | 615.6 | 1094.1 | 710.5 | 595.5 | 1.26 | 2.23 | 1.45 | 1.22 |
| | | | | | | [4] 2.324 | | | | 386.8 | | | | 0.79 |
| Sub-Truncated | 5 | 501 | 204 | 2.814 | 2.715 | [4] 2.680 | 550.2 | 686.5 | 616.5 | 592.9 | 1.10 | 1.37 | 1.23 | 1.18 |

| | | | | | | | | | | | | | | |
|---|---|---|---|---|---|---|---|---|---|---|---|---|---|---|
| Cuboctahedron 501 | | | | | | | | | | | | | | |
| | | | | | | [6] 2.539 | | | | 504.0 | | | | 1.01 |
| | | | | | | [8] 2.324 | | | | 386.8 | | | | 0.77 |
| Sub-Rhombicubocta-hedron 514 | 5 | 514 | 198 | 2.694 | 2.516 | [3] 2.454 | 457.5 | 602.2 | 490.5 | 455.1 | 0.89 | 1.17 | 0.95 | 0.89 |
| | | | | | | [4] 2.324 | | | | 386.8 | | | | 0.75 |
| Sub-Cuboctahedron 549 | 5 | 549 | 240 | 3.287 | 2.847 | [3] 2.684 | 615.6 | 1094.1 | 710.5 | 595.5 | 1.12 | 1.99 | 1.29 | 1.08 |
| | | | | | | [4] 2.324 | | | | 386.8 | | | | 0.70 |
| Cuboctahedron 561 | 5 | 561 | 252 | 3.287 | 2.847 | [3] 2.684 | 615.6 | 1094.1 | 710.5 | 595.5 | 1.10 | 1.95 | 1.27 | 1.06 |
| | | | | | | [4] 2.324 | | | | 386.8 | | | | 0.69 |
| Sub-Truncated Cuboctahedron 586 | 5 | 586 | 246 | 2.814 | 2.715 | [4] 2.680 | 550.2 | 686.5 | 616.5 | 592.9 | 0.94 | 1.18 | 1.06 | 1.02 |
| | | | | | | [6] 2.539 | | | | 504.0 | | | | 0.86 |
| | | | | | | [8] 2.324 | | | | 386.8 | | | | 0.66 |
| Sub-Truncated Cuboctahedron 605 | 5 | 605 | 248 | 2.814 | 2.715 | [4] 2.680 | 550.2 | 686.5 | 616.5 | 592.9 | 0.91 | 1.13 | 1.02 | 0.98 |
| | | | | | | [6] 2.539 | | | | 504.0 | | | | 0.83 |
| | | | | | | [8] 2.324 | | | | 386.8 | | | | 0.64 |
| Sub-Cuboctahedron 610 | 5 | 610 | 270 | 3.287 | 2.847 | [3] 2.684 | 615.6 | 1094.1 | 710.5 | 595.5 | 1.01 | 1.79 | 1.16 | 0.98 |
| | | | | | | [4] 2.324 | | | | 386.8 | | | | 0.63 |

| | | | | | | | | | | | | | | |
|---|---|---|---|---|---|---|---|---|---|---|---|---|---|---|
| Sub-Truncated Cube 641 | 5 | 641 | 284 | 3.425 | 3.287 | [3] 3.240 | 711.9 | 1238.0 | 1094.0 | 1047.4 | 1.11 | 1.93 | 1.71 | 1.63 |
| | | | | | | [8] 2.324 | | | | 386.8 | | | | 0.60 |
| Sub-Truncated Cube 658 | 5 | 658 | 294 | 3.425 | 3.287 | [3] 3.240 | 711.9 | 1238.0 | 1094.0 | 1047.4 | 1.08 | 1.88 | 1.66 | 1.59 |
| | | | | | | [8] 2.324 | | | | 386.8 | | | | 0.59 |
| Sub-Octahedron 338 | 6 | 338 | 192 | 2.732 | 1.932 | 1.577 | 199.9 | 627.9 | 222.0 | 120.8 | 0.59 | 1.86 | 0.66 | 0.36 |
| Sub-Octahedron 399 | 6 | 394 | 174 | 2.732 | 1.932 | 1.577 | 199.9 | 627.9 | 222.0 | 120.8 | 0.51 | 1.59 | 0.56 | 0.31 |
| Sub-Truncated Octahedron 459 | 6 | 459 | 234 | 3.054 | 2.898 | [4] 2.732 | 599.4 | 877.5 | 749.3 | 627.9 | 1.31 | 1.91 | 1.63 | 1.37 |
| | | | | | | [6] 2.366 | | | | 407.8 | | | | 0.89 |
| Rhombicuboctahedron 579 | 6 | 579 | 210 | 3.166 | 2.957 | [3] 2.884 | 742.7 | 977.5 | 796.3 | 738.7 | 1.28 | 1.69 | 1.38 | 1.28 |
| | | | | | | [4] 2.732 | | | | 627.9 | | | | 1.08 |
| Truncated Octahedron 586 | 6 | 586 | 272 | 3.054 | 2.898 | [4] 2.732 | 599.4 | 877.5 | 749.3 | 627.9 | 1.02 | 1.50 | 1.28 | 1.07 |
| | | | | | | [6] 2.366 | | | | 407.8 | | | | 0.70 |
| Sub-Truncated Cuboctahedron 675 | 6 | 675 | 270 | 3.308 | 3.191 | [4] 3.150 | 893.1 | 1114.4 | 1000.8 | 962.5 | 1.32 | 1.65 | 1.48 | 1.43 |
| | | | | | | [6] 2.984 | | | | 818.2 | | | | 1.21 |
| | | | | | | [8] 2.732 | | | | 627.9 | | | | 0.93 |
| Sub-Rhombicubocta- | 6 | 730 | 264 | 3.166 | 2.957 | [3] 2.884 | 742.7 | 977.5 | 796.3 | 738.7 | 1.02 | 1.34 | 1.09 | 1.01 |

| | | | | | | | | | | | | | | |
|---|---|---|---|---|---|---|---|---|---|---|---|---|---|---|
| hedron 730 | | | | | | | | | | | | | | |
| | | | | | | [4] 2.732 | | | | 627.9 | | | | 0.86 |
| Rhombicubocta-hedron 755 | 6 | 755 | 254 | 3.166 | 2.957 | [3] 2.884 | 742.7 | 977.5 | 796.3 | 738.7 | 0.98 | 1.29 | 1.05 | 0.98 |
| | | | | | | [4] 2.732 | | | | 627.9 | | | | 0.83 |
| Snub Cube 755 | 6 | 755 | 254 | 3.213 | 2.982 | [3] 2.901 | 792.8 | 1021.2 | 816.6 | 752.0 | 1.05 | 1.35 | 1.08 | 1.00 |
| | | | | | | [4] 2.732 | | | | 627.9 | | | | 0.83 |
| Sub-Truncated Octahedron 802 | 6 | 802 | 312 | 3.054 | 2.898 | [4] 2.732 | 599.4 | 877.5 | 749.3 | 627.9 | 0.75 | 1.09 | 0.93 | 0.78 |
| | | | | | | [6] 2.366 | | | | 407.8 | | | | 0.51 |
| Truncated Cuboctahedron 863 | 6 | 863 | 314 | 3.308 | 3.191 | [4] 3.150 | 893.1 | 1114.4 | 1000.8 | 962.5 | 1.03 | 1.29 | 1.16 | 1.12 |
| | | | | | | [6] 2.984 | | | | 818.2 | | | | 0.95 |
| | | | | | | [8] 2.732 | | | | 627.9 | | | | 0.73 |
| Sub-Truncated Octahedron 898 | 6 | 898 | 312 | 3.054 | 2.898 | [4] 2.732 | 599.4 | 877.5 | 749.3 | 627.9 | 0.67 | 0.98 | 0.84 | 0.70 |
| | | | | | | [6] 2.366 | | | | 407.8 | | | | 0.46 |
| Sub-Cuboctahedron 911 | 6 | 911 | 350 | 3.863 | 3.346 | [3] 3.154 | 999.4 | 1776.0 | 1153.5 | 966.8 | 1.10 | 1.95 | 1.27 | 1.06 |
| | | | | | | [4] 2.732 | | | | 627.9 | | | | 0.69 |
| Cuboctahedron 923 | 6 | 923 | 362 | 3.863 | 3.346 | [3] 3.154 | 999.4 | 1776.0 | 1153.5 | 966.8 | 1.08 | 1.92 | 1.25 | 1.05 |
| | | | | | | [4] 2.732 | | | | 627.9 | | | | 0.68 |

| | | | | | | | | | | | | | | |
|---|---|---|---|---|---|---|---|---|---|---|---|---|---|---|
| Sub-Truncated Cuboctahedron 970 | 6 | 970 | 360 | 3.308 | 3.191 | [4] 3.150 | 893.1 | 1114.4 | 1000.8 | 962.5 | 0.92 | 1.15 | 1.03 | 0.99 |
| | | | | | | [6] 2.984 | | | | 818.2 | | | | 0.84 |
| | | | | | | [8] 2.732 | | | | 627.9 | | | | 0.65 |
| Sub-Truncated Cube 1043 | 6 | 1043 | 402 | 4.026 | 3.863 | [3] 3.808 | 1155.7 | 2009.7 | 1776.0 | 1700.3 | 1.11 | 1.93 | 1.70 | 1.63 |
| | | | | | | [8] 2.732 | | | | 627.9 | | | | 0.60 |
| Truncated Cube 1074 | 6 | 1074 | 416 | 4.026 | 3.863 | [3] 3.808 | 1155.7 | 2009.7 | 1776.0 | 1700.3 | 1.08 | 1.87 | 1.65 | 1.58 |
| | | | | | | [8] 2.732 | | | | 627.9 | | | | 0.58 |
| Sub-Truncated Cube 1091 | 6 | 1091 | 426 | 4.026 | 3.863 | [3] 3.808 | 1155.7 | 2009.7 | 1776.0 | 1700.3 | 1.06 | 1.84 | 1.63 | 1.56 |
| | | | | | | [8] 2.732 | | | | 627.9 | | | | 0.58 |

## Section S5 – Example calculations

The expected structures (closest or best match) of several gold nanoparticles synthesized by our group[S2-S5] are summarized in Table S5 (high resolution TEM images are available free of charge in the electronic supporting information in most cases). The models used are listed and those highlighted in red font were considered the closest match to the gold nanoparticles experimentally observed in the TEM images. The gold nanoparticles Au1, Au2, Au4, Au5, Au10, and Au11 (No. 7, 8, 10, 11, 16, 17) had TEM images with sufficiently high resolution (see images in the original papers and their supporting information), making the choice for the most appropriate model easy (as outlined in the main text, a sufficiently high quality of TEM images is important). The structures and compositions of the other gold nanoparticles were estimated from their size distribution histograms.

The estimated core structures of the gold nanoparticles reported by Negishi *et al.*[S6] are shown in Table S6. Their structures have previously been estimated *via* DFT calculations. Our models and tables help analyze them and select the best match for the core structure.

**Table S5.** Lists of gold nanoparticles and their model structures.

| No. | Ref. | Au-NP | *D* / nm | Model | $D_c$ / nm | $D_m$ / nm | $D_i$ / nm | *h* / nm | *w* / nm |
|---|---|---|---|---|---|---|---|---|---|
| 1 | [S2] | Au-*R***1** | 2.47 ± 0.18 | Truncated Icosidodecahedron 437 | 2.671 | 2.647 | [4] 2.624<br>[6] 2.577<br>[12] 2.417 | | |
| | | | | Sub-Truncated Cuboctahedron 466 | 2.814 | 2.715 | [4] 2.680<br>[6] 2.539<br>[8] 2.324 | | |
| | | | | Sub-Truncated Icosahedron 489 | 2.738 | 2.681 | [5] 2.571<br>[6] 2.505 | | |
| 2 | [S2] | Au-*R***2** | 2.13 ± 0.18 | Sub-Rhombicosidodecahedron 267 | 2.182 | 2.126 | [3] 2.107<br>[4] 2.069<br>[5] 2.018 | | |
| | | | | Sub-Truncated Cuboctahedron 297 | 2.321 | 2.239 | [4] 2.211<br>[6] 2.094 | | |

| | | | | | | | | | |
|---|---|---|---|---|---|---|---|---|---|
| | | | | | | | [8] 1.917 | | |
| | | | | Icosahedron 309 | 2.592 | 2.205 | 2.060 | | |
| 3 | [S2] | Au-*R***3** | 1.24 ± 0.28 | Icosahedron 55 | 1.440 | 1.225 | 1.144 | | |
| | | | | Ino's Decahedron 55 | | | | 1.44 | 1.32 |
| | | | | Cuboctahedron 55 | 1.559 | 1.350 | [3] 1.273 [4] 1.103 | | |
| 4 | | Au-*S***1** | 1.73 ± 0.20 | Icosidodecahedron 145 | 1.867 | 1.776 | [3] 1.744 [5] 1.588 | | |
| | | | | Icosahedron 147 | 2.016 | 1.715 | 1.602 | | |
| | | | | Cuboctahedron 147 | 2.135 | 1.849 | [3] 1.743 [4] 1.510 | | |
| | | | | Dodecahedron 165 | 2.175 | 2.031 | 1.728 | | |
| 5 | [S2] | Au-*S***2** | 1.08 ± 0.24 | Marks' Decahedron 29 | | | | 1.15 | 1.09 |
| 6 | [S2] | Au-*S***3** | 1.09 ± 0.23 | Dodecahedron 33 | 1.203 | 1.124 | 0.956 | | |
| | | | | Truncated Octahedron 38 | 1.233 | 1.169 | [4] 1.103 [6] 0.955 | | |
| | | | | Ino's Decahedron 39 | | | | 1.15 | 1.32 |
| 7 | [S3] | Au1 | 1.65 ± 0.39 | Ino's Decahedron 116 | | | | 1.73 | 1.83 |
| | | | | Marks' Decahedron 146 | | | | 1.73 | 2.12 |
| | | | | Truncated Icosahedron 135 | 1.758 | 1.722 | [5] 1.651 [6] 1.608 | | |
| | | | | Sub-Truncated Octahedron 116 | 1.688 | 1.601 | [4] 1.510 [6] 1.308 | | |
| | | | | Cuboctahedron 147 | 2.135 | 1.849 | [3] 1.743 [4] 1.510 | | |
| 8 | [S3] | Au2 | 1.54 ± 0.38 | Ino's Decahedron 71 | | | | 1.73 | 1.32 |

| | | | | | | | | | |
|---|---|---|---|---|---|---|---|---|---|
| | | | | Marks' Decahedron 75 | | | | 1.44 | 1.61 |
| | | | | Dodecahedron 75 | 1.680 | 1.570 | 1.335 | | |
| | | | | Sub-Truncated Octahedron 116 | 1.688 | 1.601 | [4] 1.510 [6] 1.308 | | |
| 9 | [S3, S4] | Au3 | 3.50 ± 0.81 | —[a] | | | | | |
| 10 | | Au4 | 1.20 ± 0.23 | Marks' Decahedron 29 | | | | 1.15 | 1.09 |
| | | | | Ino's Decahedron 39 | | | | 1.15 | 1.32 |
| | | | | Icosidodecahedron 43 | 1.268 | 1.206 | [3] 1.184 [4] 1.079 | | |
| | | | | Truncated Octahedron 38 | 1.233 | 1.169 | [4] 1.103 [6] 0.955 | | |
| 11 | [S3] | Au5 | 1.75 ± 0.40 | Marks' Decahedron 146 | | | | 1.73 | 2.12 |
| | | | | Ino's Decahedron 147 | | | | 2.02 | 1.83 |
| | | | | Icosahedron 147 | 2.016 | 1.715 | 1.602 | | |
| | | | | Cuboctahedron 147 | 2.135 | 1.849 | [3] 1.743 [4] 1.510 | | |
| | | | | Truncated Octahedron 201 | 2.143 | 2.033 | [4] 1.917 [6] 1.660 | | |
| 12 | [S3] | Au6 | —[a] | | | | | | |
| 13 | [S3] | Au7 | 1.58 ± 0.44 | Marks' Decahedron 101 | | | | 1.44 | 1.83 |
| | | | | Rhombicosidodecahedron 115 | 1.717 | 1.674 | [3] 1.659 [4] 1.629 [5] 1.588 | | |
| | | | | Sub-Truncated Octahedron 116 | 1.688 | 1.601 | [4] 1.510 [6] 1.308 | | |
| 14 | [S3] | Au8 | 1.93 ± 0.47 | Marks' Decahedron 238 | | | | 2.30 | 2.12 |

| | | | | | | | | | |
|---|---|---|---|---|---|---|---|---|---|
| | | | | Icosidodecahedron 237 | 2.268 | 2.157 | [3] 2.119 [5] 1.930 | | |
| | | | | Truncated Octahedron 201 | 2.143 | 2.033 | [4] 1.917 [6] 1.660 | | |
| | | | | Sub-Rhombicuboctahedron 249 | 2.222 | 2.075 | [3] 2.024 [4] 1.917 | | |
| 15 | [S3] | Au9 | 1.31 ± 0.29 | Ino's Decahedron 71 | | | | 1.73 | 1.32 |
| | | | | Icosahedron 55 | 1.440 | 1.225 | 1.144 | | |
| | | | | Cuboctahedron 55 | 1.559 | 1.350 | [3] 1.273 [4] 1.103 | | |
| 16 | [S3] | Au10 | 1.57 ± 0.43 | Marks' Decahedron 101 | | | | 1.73 | 1.61 |
| | | | | Rhombicosidodecahedron 115 | 1.717 | 1.674 | [3] 1.659 [4] 1.629 [5] 1.588 | | |
| | | | | Sub-Truncated Octahedron 116 | 1.688 | 1.601 | [4] 1.510 [6] 1.308 | | |
| 17 | [S3] | Au11 | 1.61 ± 0.42 | Marks' Decahedron 101 | | | | 1.73 | 1.61 |
| | | | | Truncated Icosahedron 135 | 1.758 | 1.722 | [5] 1.651 [6] 1.608 | | |
| | | | | Icosahedron 147 | 2.016 | 1.715 | 1.602 | | |
| | | | | Sub-Truncated Octahedron 116 | 1.688 | 1.601 | [4] 1.510 [6] 1.308 | | |
| | | | | Cuboctahedron 147 | 2.135 | 1.849 | [3] 1.743 [4] | | |

| | | | | | | | |
|---|---|---|---|---|---|---|---|
| | | | | | | | 1.510 |
| 18 | [S5] | AuNP1 | 4.09 | Cuboctahedron 1415 | 4.439 | 3.844 | [3] 3.625<br>[4] 3.139 |
| | | | | Sub-Truncated Cuboctahedron 1416 | 3.801 | 3.667 | [4] 3.619<br>[6] 3.429<br>[8] 3.139 |

[a] Could not be isolated in pure form (and analyzed here) due to amphiphilicity of the resulting mixed-monolayer-protected Au NPs.

**Table S6.** Lists of gold nanoparticles and their core structures.

| **No.** | **Ref.** | **Au-NP** | ***D* / nm** | **Model** | **$D_c$ / nm** | **$D_m$ / nm** | **$D_i$ / nm** |
|---|---|---|---|---|---|---|---|
| 19 | [S6] | $Au_{\sim 226}(SC_{12})_{\sim 76}$ | 2.0 ± 0.5 | Truncated Octahedron 201 | 2.143 | 2.033 | [4] 1.917<br>[6] 1.660 |
| | | | | Sub-Truncated Cuboctahedron 236 | 2.321 | 2.239 | [4] 2.211<br>[6] 2.094<br>[8] 1.917 |
| 20 | [S6] | $Au_{\sim 253}(SC_{12})_{\sim 90}$ | 2.1 ± 0.4 | Sub-Truncated Cuboctahedron 236 | 2.321 | 2.239 | [4] 2.211<br>[6] 2.094<br>[8] 1.917 |
| | | | | Sub-Rhombicuboctahedron 249 | 2.222 | 2.075 | [3] 2.024<br>[4] 1.917 |
| 21 | [S6] | $Au_{329}(SC_{12})_{84}$ | 2.2 ± 0.4 | Sub-Truncated Cuboctahedron 297 | 2.321 | 2.239 | [4] 2.211<br>[6] 2.094<br>[8] 1.917 |
| | | | | Cuboctahedron 309 | 2.711 | 2.348 | [3] 2.214<br>[4] |

| | | | | | | | |
|---|---|---|---|---|---|---|---|
| | | | | Sub-Truncated Cuboctahedron 316 | 2.321 | 2.239 | 1.917 [4] 2.211 [6] 2.094 [8] 1.917 |
| 22 | [S6] | $Au_{\text{-}356}(SC_{12})_{\text{-}112}$ | 2.2 ± 0.3 | Cuboctahedron 340 | 2.711 | 2.348 | [3] 2.214 [4] 1.917 |
| | | | | Sub-Truncated Octahedron 314 | 2.599 | 2.465 | [4] 2.324 [6] 2.013 |
| 23 | [S6] | $Au_{\text{-}520}(SC_{12})_{\text{-}130}$ | 2.4 ± 0.2 | Sub-Truncated Cuboctahedron 466 | 2.814 | 2.715 | [4] 2.680 [6] 2.539 [8] 2.324 |
| | | | | Sub-Cuboctahedron 490 | 3.287 | 2.847 | [3] 2.684 [4] 2.324 |
| | | | | Sub-Truncated Cuboctahedron 501 | 2.814 | 2.715 | [4] 2.680 [6] 2.539 [8] 2.324 |

**Section S6 – *xyz* Coordinates**

The xzy-coordinates for all gold nanoclusters are provided as separate electronic supplementary materials. These xyz-coordinate files are provided in standard format that can be analyzed just like crystal structures using CIF format.

**References**

[S1] Models and equations from: Wolfram MathWorld, http://mathworld.wolfram.com/ and Visual Polyhedra, http://dmccooey.com/polyhedra/index.html

[S2] Mori, T.; Sharma, A.; Hegmann, T. Significant Enhancement of the Chiral Correlation Lengths in Nematic Liquid Crystals by Gold Nanoparticle Surfaces Featuring Axially Chiral Binaphthyl Ligands. *ACS Nano* **10**, 1552-1564 (2016).

[S3] Qi, H.; Hegmann, T. Post-Synthesis Racemization and Place Exchange Reactions. Another Step to Unravel The Origin of Chirality for Chiral Ligand-Capped Gold Nanoparticles. *J. Am. Chem. Soc.* **130**, 14201-14206 (2008).

[S4] Qi, H.; Hegmann, T. Formation of Periodic Stripe Patterns in Nematic Liquid Crystals Doped with Functionalized Gold Nanoparticles. *J. Mater. Chem.* **16**, 4197-4205 (2006).

[S5] Mirzaei, J.; Urbanski, M.; Kitzerow, H.-S.; Hegmann, T. Synthesis of Liquid Crystal Silane Functionalized Gold Nanoparticles and Their Effects on Optical and Electro-Optic Properties of a Structurally Related Nematic Liquid Crystal. *ChemPhysChem* **15**, 1381-1394 (2014).

[S6] Negishi, Y.; Nakazaki, T.; Malola, S.; Takano, S.; Niihori, Y.; Kurashige, W.; Yamazoe, S.; Tsukuda, T.; Häkkinen, H. A Critical Size for Emergence of Nonbulk Electronic and Geometric Structures in Dodecanethiolate-Protected Au Clusters. *J. Am. Chem. Soc.* **137**, 1206-1212 (2015).
